# Correction: A comprehensive analysis of coregulator recruitment, androgen receptor function and gene expression in prostate cancer

**DOI:** 10.7554/eLife.33738

**Published:** 2017-11-22

**Authors:** Song Liu, Sangeeta Kumari, Qiang Hu, Dhirodatta Senapati, Varadha Balaji Venkadakrishnan, Dan Wang, Adam D DePriest, Simon E Schlanger, Salma Ben-Salem, Malyn May Valenzuela, Belinda Willard, Shaila Mudambi, Wendy M Swetzig, Gokul M Das, Mojgan Shourideh, Shahriah Koochekpour, Sara Moscovita Falzarano, Cristina Magi-Galluzzi, Neelu Yadav, Xiwei Chen, Changshi Lao, Jianmin Wang, Jean-Noel Billaud, Hannelore V Heemers

Liu S, Kumari S, Hu Q, Senapati D, Venkadakrishnan VB, Wang D, DePriest AD, Schlanger SE, Ben-Salem S, Valenzuela MM, Willard B, Mudambi S, Swetzig WM, Das GM, Shourideh M, Koochekpour S, Falzarano SM, Magi-Galluzzi C, Yadav N, Chen X, Lao C, Wang J, Billaud JN, Heemers HV. 2017. A comprehensive analysis of coregulator recruitment, androgen receptor function and gene expression in prostate cancer. *eLife* **6**:e28482. doi: 10.7554/eLife.28482.

Published 18, August 2017

In the Results section, there was a typo in Figure 3F, where it should read 62 instead of 40 in the WDR77-siRNA only side of the Venn diagram. We have corrected this error. Please note that this correction does not affect the results and conclusions of the original paper.

The corrected version of Figure 3F is shown here:

**Figure 1:**
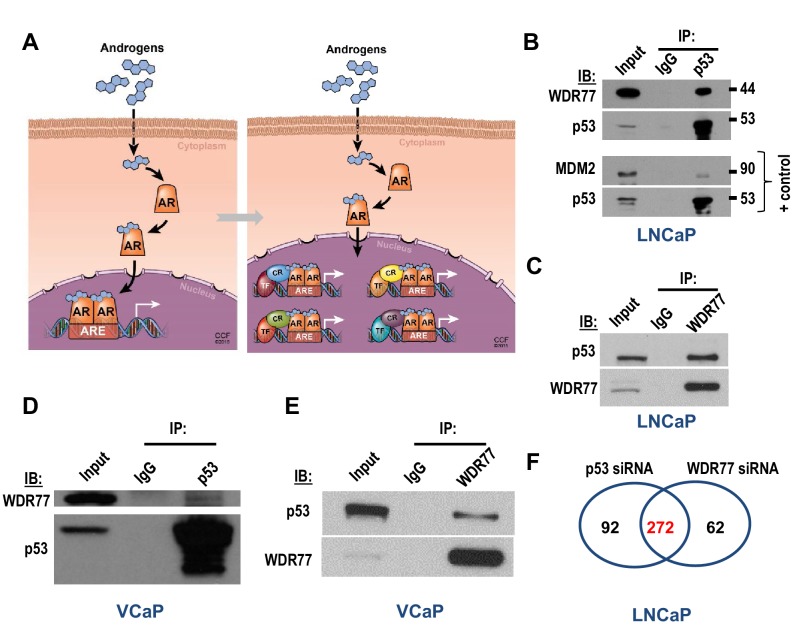


The originally published version of Figure 3F is also shown for reference:

**Figure 2:**
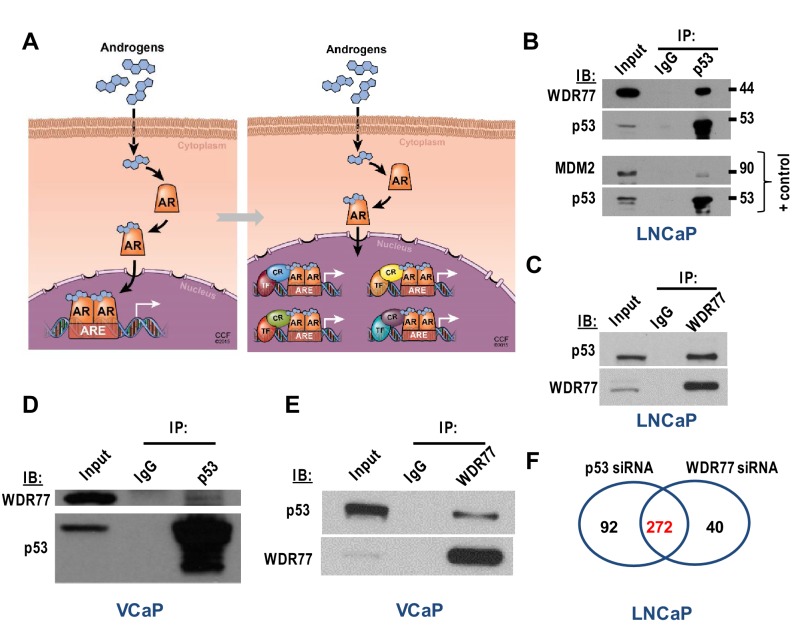


The article has been corrected accordingly.

