# Supplementary material for: A comprehensive analysis of coregulator recruitment, androgen receptor function and gene expression in prostate cancer
Source: eLife. 2017 Aug 18;6:e28482. doi: 10.7554/eLife.28482 (PMC5608510; doi:10.7554/eLife.28482)

**Figure 1 – Source Data 1.**  **Gene specificity and context-dependency of coregulator contribution to androgen regulation of AR target gene expression.** co+, androgen regulation is increased (+) after loss of coregulator and direction of regulation remains consistent (co); co-, androgen regulation is decreased (-) after loss of coregulator and direction of regulation remains consistent (co); op+, androgen regulation is increased (+) after loss of coregulator but direction of regulation is opposite (op); op-, androgen regulation is decreased (-) after loss of coregulator and direction of regulation is opposite (op). Results reflect the effect of 48-hour treatment of LNCaP cells with 5nM R1881. R1881 or vehicle treatment was administered 42 hours after siRNA transfection. Treatment groups contained 3 biological replicates.


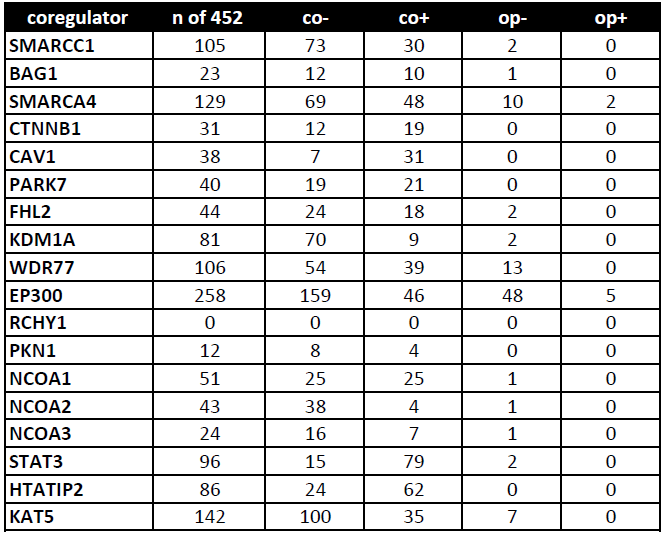

Supplement: Figure 1—source data 1. — co+, androgen regulation is increased (+) after loss of coregulator and direction of regulation remains consistent (co); co-, androgen regulation is decreased (-) after loss of coregulator and direction of regulation remains consistent (co); op+, androgen regulation is increased (+) after loss of coregulator but direction of regulation is opposite (op); op-, androgen regulation is decreased (-) after loss of coregulator and direction of regulation is opposite (op). Results reflect the effect of 48 hr treatment of LNCaP cells with 5nM R1881. R1881 or vehicle treatment was administered 42 hr after siRNA transfection. Treatment groups contained 3 biological replicates. [file elife-28482-fig1-data1.docx]
