# Supplementary material for: A comprehensive analysis of coregulator recruitment, androgen receptor function and gene expression in prostate cancer
Source: eLife. 2017 Aug 18;6:e28482. doi: 10.7554/eLife.28482 (PMC5608510; doi:10.7554/eLife.28482)
Supplement: Figure 1—source data 2. — Hypergeometric test was used to derive the statistical significance of the overlap between lists of genes. [file elife-28482-fig1-data2.docx]

**Figure 1 – Source Data 2. Summary of p-values for data presented in Figure 1C.**

Hypergeometric test was used to derive the statistical significance of the overlap between lists of genes.
