# Supplementary material for: A comprehensive analysis of coregulator recruitment, androgen receptor function and gene expression in prostate cancer
Source: eLife. 2017 Aug 18;6:e28482. doi: 10.7554/eLife.28482 (PMC5608510; doi:10.7554/eLife.28482)

**Figure 2 – Source Data 1. Overview of number of TF binding sites (TFBSs), and AR or GR binding sites (BSs) that are identified in ARBSs of coregulator-dependent AR target gene sets using Cistrome project tools.**


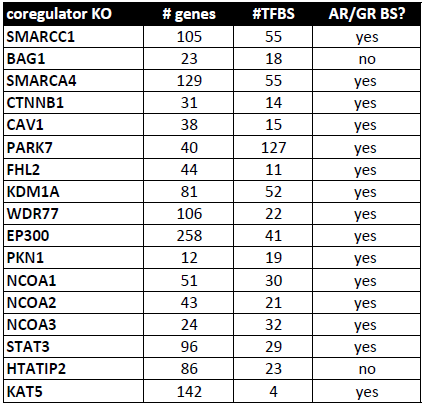

Supplement: Figure 2—source data 1. [file elife-28482-fig2-data1.docx]
