# Supplementary material for: A comprehensive analysis of coregulator recruitment, androgen receptor function and gene expression in prostate cancer
Source: eLife. 2017 Aug 18;6:e28482. doi: 10.7554/eLife.28482 (PMC5608510; doi:10.7554/eLife.28482)

**Figure 2 – Source Data 2. Overview of the number of Ingenuity Pathway Analysis categories that associate with individual coregulator-dependent AR target gene signatures.**


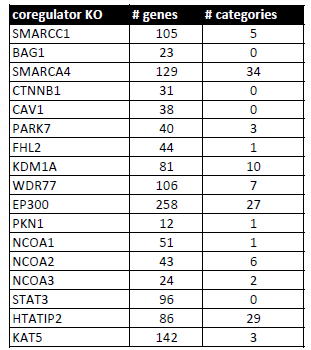

Supplement: Figure 2—source data 2. [file elife-28482-fig2-data2.docx]
