# Supplementary material for: A comprehensive analysis of coregulator recruitment, androgen receptor function and gene expression in prostate cancer
Source: eLife. 2017 Aug 18;6:e28482. doi: 10.7554/eLife.28482 (PMC5608510; doi:10.7554/eLife.28482)
Supplement: Figure 5—source data 1. [file elife-28482-fig5-data1.docx]

**Figure 5 – Source Data 1.**  PGAM5 peptides identified after IP-mass spectrometry

| Actual Mass | Observed | Charge | Delta PPM | Residues | Sequence |
| --- | --- | --- | --- | --- | --- |
| 1,117.60 | 559.8058 | 2 | -0.939 | 153-162 | (R )AIETTDIISR(H) |
| 1,015.53 | 508.7717 | 2 | -1.168 | 126-134 | (R )EQAELTGLR(L) |
| 1,398.65 | 467.2245 | 3 | -0.884 | 105-116 | (R )HSQYHVDGSLEK (D) |
| 705.3805 | 353.6975 | 2 | -0.6258 | 204-209 | (R )IEAAFR(N) |
| 700.4474 | 351.231 | 2 | -1.58 | 135-141 | (R )LASLGLK(F) |
| 1,290.59 | 646.3041 | 2 | -0.2852 | 77-88 | (R )NVESGEEELASK(L) |
| 809.4209 | 405.7177 | 2 | -1.312 | 163-169 | (R )HLPGVcK(V) |
