# Supplementary material for: A comprehensive analysis of coregulator recruitment, androgen receptor function and gene expression in prostate cancer
Source: eLife. 2017 Aug 18;6:e28482. doi: 10.7554/eLife.28482 (PMC5608510; doi:10.7554/eLife.28482)
Supplement: Figure 6—source data 1. — For panels A, C, D, and E, p-values were derived using welch two sample t-test. Values are compared to those obtained from the control siRNA group with changes considered significant at p<0.05. For panel B, p-values are derived using paired t-test. The fold change in values obtained after R1881 treatment is calculated for each siRNA group and values for specific siRNA groups are compared to those derived from the control siRNA group. Changes are considered significant at p<0.05. [file elife-28482-fig6-data1.docx]

**Figure 6 – Source Data 1. Summary of p-values for data presented in Figure 6.**

For panels A, C, D, and E, p-values were derived using welch two sample t-test. Values are compared to those obtained from the control siRNA group with changes considered significant at p<0.05. For panel B, p-values are derived using paired t-test. The fold change in values obtained after R1881 treatment is calculated for each siRNA group and values for specific siRNA groups are compared to those derived from the control siRNA group. Changes are considered significant at p<0.05.

**Panel A**

| siRNA | p-value |
| --- | --- |
| AR | 0.000961 |
| WDR77 | 0.047881 |
| PGAM5 | 0.000600 |
| HES6 | 0.002965 |
| AGR2 | 0.002617 |
| GNB4 | 0.666376 |
| RAB27A | 0.001308 |

**Panel B**

| siRNA | p-value |
| --- | --- |
| AR | 0.005092 |
| WDR77 | 0.030428 |
| PGAM5 | 0.004541 |
| HES6 | 0.002531 |
| AGR2 | 0.003461 |
| GNB4 | 0.178950 |
| RAB27A | 0.003207 |

**Panel C - C135Y**

| siRNA | p-value |
| --- | --- |
| AR | 0.000703 |
| WDR77 | 0.034056 |
| PGAM5 | 0.000922 |
| HES6 | 0.009110 |
| AGR2 | 0.027978 |
| GNB4 | 0.008411 |
| RAB27A | 0.005748 |

**Panel C – N239T**

| siRNA | p-value |
| --- | --- |
| AR | 1.15164E-05 |
| WDR77 | 0.000871 |
| PGAM5 | 9.39E-05 |
| HES6 | 0.004487 |
| AGR2 | 0.136339 |
| GNB4 | 0.004757 |
| RAB27A | 0.014016 |

**Panel D**

| siRNA | p-value |
| --- | --- |
| AR | 0.003178 |
| WDR77 | 0.017838 |
| PGAM5 | 0.002087 |
| HES6 | 0.009171 |
| AGR2 | 0.020509 |
| GNB4 | 0.006313 |
| RAB27A | 0.002024 |

**Panel E – G1**

| siRNA | p-value |
| --- | --- |
| AR | 0.001285 |
| WDR77 | 0.011233 |
| PGAM5 | 0.003881 |
| HES6 | 0.004128 |
| AGR2 | 0.117856 |
| GNB4 | 0.126004 |
| RAB27A | 5.28925E-06 |

**Panel E - S**

| siRNA | p-value |
| --- | --- |
| AR | 6.74394E-07 |
| WDR77 | 0.000474 |
| PGAM5 | 0.008579 |
| HES6 | 0.006334 |
| AGR2 | 0.039579 |
| GNB4 | 0.912667 |
| RAB27A | 0.009598 |

**Panel E – G2**

| siRNA | p-value |
| --- | --- |
| AR | 9.70848E-05 |
| WDR77 | 0.483058 |
| PGAM5 | 0.020841 |
| HES6 | 0.000991 |
| AGR2 | 0.000107 |
| GNB4 | 0.063685 |
| RAB27A | 0.000823 |
