# Supplementary material for: A comprehensive analysis of coregulator recruitment, androgen receptor function and gene expression in prostate cancer
Source: eLife. 2017 Aug 18;6:e28482. doi: 10.7554/eLife.28482 (PMC5608510; doi:10.7554/eLife.28482)
Supplement: Supplementary file 1. — (A) Overview of genes included in custom Agilent oligoarray Rows, categories of genes included on 8 × 15K custom Agilent oligoarray. Columns, Number of genes identified for inclusion on the array, and number of genes for which Agilent catalogue probes were available for inclusion. (B) Overview of 452 AR target gene signature Gene name, HUGO gene symbol; FC, fold change (C) Overview of coregulators considered, prioritized and withheld for analysis A PudMed search for papers that contain the terms ‘AR’ and ‘CaP’ in their title and/or abstract was performed. Abstracts fulfilling these criteria were screened for reference to coregulator function, and if so, full-length papers were reviewed individually to verify description of a bona fide AR-associated coregulator. Left to right: Column 1: 181 coregulators for which literature search was done. Column 2: 51 coregulators for which differential protein expression has been reported in CaP when compared to benign prostate (yes entries). Column 3: 22 coregulators for which differential expression in CaP correlated with aggressive disease, and were analyzed in Figures 4–6 (yes entries). Column 4: 18 coregulators for which siRNA-mediated silencing did not affect AR expression, CaP cell morphology or CaP cell survival and were included in final analyses (yes entries). [file elife-28482-supp1.docx]

**Supplementary File 1. Design of oligoarray, overview of AR target genes studied, and overview of coregulators considered for analysis.**

|  | | |
| --- | --- | --- |
|  | | |
|  | | |
|  | | |
|  | | |
|  | | |
| Supplementary File 1A. 8 x 15K Agilent oligoarray | | |
| Category | **Genes identified**  **for inclusion**  **(n)** | **Genes with available probe**  **(n)** |
| AR target genes | 900 | 898 |
| Coregulators | 181 | 180 |
| Housekeeping genes | 58 | 58 |
| Assay controls | NA | >50 |

NA = not applicable

| Supplementary File 1B. Genes identified with ≥ 2-fold change in expression in response to androgen exposure. | | | |
| --- | --- | --- | --- |
| Gene Name | **RefSeq ID** | **Log2FC** | **P value** |
| ABCC1 | NM_019862 | 1.016771013 | 0.00235921 |
| ABCC4 | NM_005845 | 2.919594802 | 4.24E-05 |
| ABCG1 | NM_207627 | -1.490900381 | 0.000135091 |
| ABHD2 | NM_007011 | 2.616504929 | 2.30E-06 |
| ABI2 | NM_005759 | -1.318505954 | 0.00036807 |
| ACAD8 | NM_014384 | 1.344714382 | 6.51E-06 |
| ACAT1 | NM_000019 | -1.52402911 | 9.37E-05 |
| ACAT2 | NM_005891 | 1.154517734 | 1.09E-05 |
| ACHE | NM_015831 | 2.393966166 | 0.000107549 |
| ACSL3 | NM_004457 | 3.602790679 | 1.24E-07 |
| ACTG2 | NM_001615 | 5.25234073 | 8.21E-09 |
| ADAM9 | NM_003816 | 1.035439167 | 0.000364828 |
| ADARB1 | NR_027673 | 1.311169308 | 1.96E-05 |
| ADD3 | NM_016824 | -1.018824456 | 0.001483774 |
| AES | NM_198969 | -1.127586637 | 0.008292396 |
| AFF3 | NM_002285 | 1.978001674 | 0.000424155 |
| AGPAT2 | NM_006412 | -1.247558665 | 0.003825014 |
| AGR2 | NM_006408 | 3.328740809 | 2.31E-06 |
| AIF1 | NM_004847 | -2.042825292 | 0.006280008 |
| AKAP12 | NM_144497 | 3.232665292 | 9.89E-06 |
| ALCAM | NM_001627 | -1.273898036 | 0.001613331 |
| ALDH1A3 | NM_000693 | 3.01761785 | 2.77E-07 |
| ALDH2 | NM_000690 | -1.426590861 | 5.38E-06 |
| ALDH4A1 | NM_003748 | 1.562184892 | 0.000384249 |
| AMD1 | NM_001634 | 1.084297245 | 1.69E-05 |
| AMIGO2 | NM_181847 | -5.432142437 | 6.69E-06 |
| ANKH | NM_054027 | 1.801090084 | 1.56E-05 |
| ANTXR1 | NM_053034 | -3.240012073 | 4.14E-05 |
| ANXA2 | NM_001002857 | 3.145816156 | 5.39E-07 |
| ANXA4 | NM_001153 | 1.035159758 | 2.42E-05 |
| AP1B1 | NM_001127 | 1.081664797 | 0.031463897 |
| AP2S1 | NM_004069 | 1.07803876 | 7.03E-05 |
| APOD | NM_001647 | 1.098055121 | 0.001259376 |
| APP | NM_000484 | 1.376649718 | 0.008933555 |
| APPBP2 | NM_006380 | 1.607816576 | 3.44E-05 |
| AQP5 | NM_001651 | 1.17199052 | 0.000727603 |
| AR | ENST00000374690 | -2.500234858 | 2.92E-06 |
|  | | | |
| Gene Name | **RefSeq ID** | **Log2FC** | **P value** |
| ARF4 | NM_001660 | 1.073005673 | 6.80E-05 |
| ARHGAP11A | NM_199357 | -1.561172205 | 0.016201067 |
| ARL4D | NM_001661 | -1.179179087 | 0.000516582 |
| ASRGL1 | NM_025080 | 2.295704978 | 6.57E-07 |
| ATAD2 | NM_014109 | 1.208525799 | 5.21E-05 |
| ATF3 | NM_001674 | 1.385994224 | 0.0001179 |
| ATF7 | BC042363 | 1.070931006 | 0.001839963 |
| ATP11A | NM_032189 | -1.320208793 | 0.004160926 |
| ATP1B1 | NM_001677 | -2.439327518 | 3.39E-05 |
| ATP2B4 | NM_001684 | -1.246989672 | 0.022274009 |
| AZGP1 | NM_001185 | 3.886566541 | 4.47E-09 |
| B2M | NM_004048 | 1.122482937 | 2.87E-05 |
| B4GALT1 | NM_001497 | 1.369747139 | 0.000689531 |
| BARD1 | NM_000465 | -2.606232352 | 4.45E-06 |
| BCHE | NM_000055 | -6.290401574 | 1.42E-08 |
| BMPR1A | NM_004329 | 1.582994287 | 1.94E-06 |
| BMPR1B | NM_001203 | 1.028568781 | 2.67E-05 |
| BPTF | NM_182641 | -1.307772745 | 0.001324046 |
| BTD | NM_000060 | -1.372606696 | 1.91E-05 |
| BTG2 | NM_006763 | -1.616303274 | 4.74E-06 |
| C13orf1 | NM_020456 | 1.748266115 | 0.000656314 |
| C14orf143 | NM_145231 | -1.744782211 | 2.76E-05 |
| C16orf45 | NM_033201 | -2.474554295 | 9.67E-07 |
| C1orf116 | NM_023938 | 1.806120041 | 3.91E-05 |
| C1orf21 | NM_030806 | 1.952463821 | 8.20E-06 |
| C21orf56 | NM_032261 | 1.024979966 | 0.001885504 |
| C22orf9 | NM_015264 | -1.035368088 | 0.039056272 |
| C4B | NM_001002029 | -1.837953266 | 0.000346797 |
| C9 | NM_001737 | -1.510456033 | 0.000650997 |
| CA12 | ENST00000178638 | 1.089255537 | 0.019732488 |
| CACNB3 | NM_000725 | -1.087604753 | 5.78E-05 |
| CALCRL | NM_005795 | -1.115363183 | 0.000491769 |
| CALD1 | NM_033138 | -1.747344916 | 1.61E-06 |
| CALU | NM_001219 | 1.358072939 | 0.000307975 |
| CAMKK2 | NM_172215 | 1.028239229 | 4.59E-05 |
| CBLB | NM_170662 | -2.027032354 | 0.002255199 |
| CBX1 | NM_006807 | -1.173767731 | 7.67E-06 |
| CCDC85B | NM_006848 | -1.094602552 | 0.000104487 |
| CCL5 | NM_002985 | 1.058939513 | 0.029990472 |

|  | | | |
| --- | --- | --- | --- |
| Gene Name | **RefSeq ID** | **Log2FC** | **P value** |
| CCNF | NM_001761 | -2.870810516 | 0.006268602 |
| CCNG2 | NM_004354 | -1.680999342 | 3.07E-05 |
| CD302 | NM_014880 | -1.838593293 | 8.65E-06 |
| CD4 | NM_000616 | -1.041423865 | 0.00020104 |
| CD59 | NM_203330 | -1.142359456 | 0.000560948 |
| CDC6 | NM_001254 | -1.105873053 | 4.31E-05 |
| CDH3 | NM_001793 | -2.61033525 | 2.48E-05 |
| CDK8 | NM_001260 | -2.274973763 | 4.51E-06 |
| CDKL5 | NM_003159 | -1.194232604 | 0.00638491 |
| CFHR4 | NM_006684 | -1.144350588 | 9.85E-06 |
| CIRBP | NM_001280 | -1.965016302 | 0.000140862 |
| CITED1 | NM_004143 | 1.303395977 | 3.72E-05 |
| CLDN12 | NM_012129 | 1.176067296 | 0.000134163 |
| CLDN8 | NM_199328 | 1.187112941 | 4.59E-05 |
| CLGN | NM_004362 | 1.390615449 | 0.000236918 |
| CLK2 | NM_003993 | -1.046598567 | 0.018411049 |
| CNP | NM_033133 | -1.203911695 | 0.003143943 |
| COL5A2 | NM_000393 | -5.587789996 | 3.92E-08 |
| COLEC12 | NM_130386 | -2.994205644 | 1.18E-06 |
| COPS7A | NM_016319 | -1.448124298 | 0.031394953 |
| COX17 | NM_005694 | 1.369019487 | 0.000347641 |
| CPS1 | NM_001875 | -1.260495957 | 5.60E-06 |
| CRAT | NM_000755 | -1.917782457 | 0.001978449 |
| CREB3L2 | BC063666 | 1.094191966 | 3.24E-05 |
| CREBBP | NM_004380 | -1.014085506 | 0.000322754 |
| CREM | NM_001881 | 1.021017063 | 7.69E-05 |
| CROT | NR_026585 | 1.072960657 | 0.00011498 |
| CRY1 | NM_004075 | 1.077137729 | 6.67E-05 |
| CSH1 | NM_022640 | 1.041982416 | 0.01770198 |
| CTBP1 | NM_001012614 | -2.176501485 | 0.001392986 |
| CTH | NM_001902 | 1.361990823 | 8.60E-06 |
| CTNND1 | NM_001331 | 1.07430315 | 0.001829412 |
| CTSB | NM_147780 | -1.195986039 | 0.02955374 |
| CUX1 | NM_181552 | -1.099388587 | 0.00025977 |
| CXADR | NM_001338 | 1.279675837 | 1.43E-05 |
| CXCR4 | NM_001008540 | 2.957023645 | 2.65E-06 |
| CXCR7 | NM_020311 | -4.946354463 | 5.83E-08 |
| CYP1A2 | NM_000761 | 1.218052936 | 0.000175062 |
| CYP3A7 | NM_000765 | 2.569629249 | 1.25E-06 |

|  | | | |
| --- | --- | --- | --- |
| Gene Name | **RefSeq ID** | **Log2FC** | **P value** |
| CYR61 | NM_001554 | 1.106081942 | 0.00067565 |
| DBI | NM_020548 | 1.341872572 | 1.28E-05 |
| DDB2 | NM_000107 | -1.500695989 | 6.89E-06 |
| DDC | NM_000790 | -5.222559483 | 9.32E-09 |
| DDR1 | NM_013993 | -1.085233661 | 0.000395434 |
| DDX6 | NM_004397 | -1.17784158 | 7.93E-05 |
| DHCR24 | NM_014762 | 1.94376033 | 3.21E-05 |
| DHRS2 | NM_182908 | 1.074303348 | 1.01E-05 |
| DLG4 | AF028825 | -1.257942533 | 0.033289521 |
| DLX1 | NM_178120 | 1.58536687 | 0.000514829 |
| DNAJB9 | NM_012328 | 1.420998048 | 4.67E-05 |
| DNAJC3 | NM_006260 | 1.128887601 | 0.000127201 |
| DNM1L | NM_012062 | 1.12840116 | 0.000126398 |
| DPYSL2 | NM_001386 | -2.242202295 | 0.000257638 |
| EAF2 | NM_018456 | 3.306778673 | 3.29E-06 |
| EDA | NM_001005612 | -1.521823474 | 0.040240839 |
| EFNA5 | NM_001962 | 1.056921914 | 0.003606887 |
| EIF3I | AK289882 | -1.016381244 | 0.038574744 |
| ELF3 | NM_001114309 | 1.396426247 | 3.00E-05 |
| ELL2 | NM_012081 | 2.037637754 | 3.45E-06 |
| ENO2 | NM_001975 | -1.971894407 | 0.001431769 |
| ENPP5 | BX647968 | -1.081864785 | 5.18E-05 |
| EPHA3 | NM_005233 | 1.953356688 | 0.026597607 |
| EPS8 | NM_004447 | -1.777744287 | 0.003629934 |
| ERBB2 | NM_001005862 | -1.074460901 | 7.30E-05 |
| ERO1L | ENST00000395686 | 2.034014671 | 0.000403842 |
| ESR2 | NM_001040276 | -1.357282526 | 0.03881569 |
| ETV1 | NM_004956 | 1.41335381 | 0.002115821 |
| EXT1 | NM_000127 | -1.047304355 | 0.007216163 |
| F2RL1 | NM_005242 | 2.193368717 | 7.02E-06 |
| FABP3 | BG336702 | 1.047210581 | 0.004270832 |
| FADS1 | NM_013402 | 1.391594756 | 7.05E-05 |
| FAS | NM_000043 | 1.133749543 | 0.000103664 |
| FASN | NM_004104 | 1.2672806 | 0.00363344 |
| FBXO38 | NM_205836 | 1.089195004 | 5.83E-05 |
| FERMT2 | NM_001135000 | 1.526265769 | 0.025403175 |
| FGL2 | NM_006682 | 3.584893412 | 8.84E-07 |
| FKBP5 | NM_004117 | 4.702702257 | 2.25E-07 |
| FLOT2 | NM_004475 | -1.056143029 | 2.13E-05 |

|  | | | |
| --- | --- | --- | --- |
| Gene Name | **RefSeq ID** | **Log2FC** | **P value** |
| FMO5 | NM_001144829 | 1.357476407 | 0.000537926 |
| FMOD | NM_002023 | 1.148945703 | 0.049169595 |
| FN1 | NM_212482 | -2.980177262 | 4.01E-07 |
| FOLH1 | NM_001014986 | -1.530688393 | 0.001371794 |
| FSTL1 | NM_007085 | -1.156666179 | 0.000112017 |
| FUT8 | NM_178154 | -1.411896486 | 4.59E-06 |
| GALK2 | NM_001001556 | 1.045688574 | 5.00E-05 |
| GALNT3 | NM_004482 | -2.228204501 | 4.77E-05 |
| GATA2 | NM_001145661 | -1.631580998 | 0.001321495 |
| GCG | NM_002054 | -1.201853548 | 5.44E-06 |
| GCLM | NM_002061 | 1.238357563 | 3.04E-05 |
| GCSH | NM_004483 | 1.049153765 | 0.008006035 |
| GLIPR1 | NM_006851 | 2.620220635 | 0.000264122 |
| GLRX2 | NM_016066 | 1.744343618 | 1.44E-05 |
| GLUD1 | NM_005271 | 1.093250328 | 1.03E-05 |
| GNB4 | NM_021629 | 2.042293501 | 3.34E-06 |
| GNL1 | NM_005275 | -1.244108225 | 0.011298984 |
| GOLIM4 | NM_014498 | 1.503291469 | 7.36E-05 |
| GOLPH3 | NM_022130 | 1.156898078 | 7.71E-05 |
| GPC1 | NM_002081 | -1.17470185 | 0.000146525 |
| GPI | NM_000175 | -1.268594804 | 0.023012518 |
| GRB10 | NM_001001555 | -3.584618361 | 4.59E-08 |
| GRB14 | NM_004490 | -1.139969822 | 3.04E-05 |
| GRB2 | NM_002086 | -1.066962122 | 0.015970174 |
| GREB1 | NM_014668 | 1.257541845 | 0.020905052 |
| GSR | NM_000637 | 1.169910958 | 8.92E-05 |
| GSTM3 | NM_000849 | -2.809410617 | 0.002783944 |
| GSTT2 | NM_000854 | 1.330416617 | 0.000109796 |
| GTF2E1 | NM_005513 | -1.012113672 | 3.64E-05 |
| GTF2I | NM_001518 | -1.025093644 | 0.000579723 |
| GUCY1A3 | NM_001130683 | 1.004436099 | 0.002619931 |
| GUSB | NM_000181 | -1.029144043 | 0.00044627 |
| H2AFZ | NM_002106 | 1.112149145 | 0.004276549 |
| HBG1 | NM_000559 | -1.104738818 | 0.009678499 |
| HERC1 | NM_003922 | -1.134408901 | 0.000421143 |
| HERC3 | NM_014606 | 2.81659075 | 1.27E-07 |
| HES1 | NM_005524 | 1.998996688 | 0.000337156 |
| HES6 | NM_018645 | 3.057048953 | 1.92E-08 |
| HIBADH | NM_152740 | -1.948350912 | 5.25E-07 |

|  | | | |
| --- | --- | --- | --- |
| Gene Name | **RefSeq ID** | **Log2FC** | **P value** |
| HIVEP3 | ENST00000372583 | -2.973391124 | 0.001341334 |
| HLA-E | NM_005516 | -1.005554976 | 0.019122451 |
| HMGCR | NM_000859 | 1.266418181 | 9.06E-06 |
| HMGN3 | NM_004242 | -1.104305458 | 6.09E-05 |
| HOMER2 | NM_199330 | 2.816025557 | 9.91E-07 |
| HOXB13 | NM_006361 | -1.024955898 | 2.69E-05 |
| HOXC13 | NM_017410 | -2.044032688 | 7.75E-07 |
| HOXC9 | NM_006897 | -2.188311498 | 1.36E-06 |
| HPGD | NM_000860 | 5.607654853 | 2.42E-07 |
| HSD17B14 | NM_016246 | -1.994898983 | 3.31E-06 |
| HSPA2 | NM_021979 | 2.652040931 | 0.000154896 |
| HSPA8 | NM_006597 | 1.285511336 | 0.00013957 |
| ID1 | NM_002165 | -1.906810419 | 2.80E-06 |
| ID2 | NM_002166 | 1.361849021 | 0.000166964 |
| ID3 | NM_002167 | -1.700222564 | 1.93E-05 |
| IDE | NM_004969 | 1.1578191 | 0.000210335 |
| IDH1 | NM_005896 | 1.106757797 | 1.47E-05 |
| IDI1 | NM_004508 | 1.380804796 | 4.78E-05 |
| IGF1 | NM_000618 | 2.226535851 | 8.39E-05 |
| IGF2 | NM_000612 | 2.229174667 | 0.015339367 |
| IL1RN | BC068441 | -2.34524523 | 0.005235956 |
| INHBB | NM_002193 | -1.047040396 | 4.37E-05 |
| INPP4B | NM_003866 | 1.184714185 | 0.019847561 |
| INPP5A | NM_005539 | -1.051061726 | 0.001338889 |
| INPP5D | NM_001017915 | -2.139945738 | 0.000903771 |
| INSIG1 | NM_198336 | 2.372551837 | 6.80E-07 |
| IQGAP2 | NM_006633 | 1.130907946 | 0.015014475 |
| ITGAV | NM_002210 | 2.816053716 | 0.001602242 |
| JUP | NM_021991 | -1.294706797 | 0.021174046 |
| KCNJ2 | NM_000891 | -1.160065787 | 0.000145206 |
| KDELR2 | NM_006854 | 1.370475865 | 3.74E-05 |
| KDELR3 | NM_016657 | 1.98938687 | 9.31E-06 |
| KIAA0247 | NM_014734 | -1.615746805 | 7.69E-06 |
| KLF4 | NM_004235 | 1.805720719 | 1.27E-05 |
| KLF5 | NM_001730 | 1.934009731 | 1.47E-05 |
| KLK2 | NM_005551 | 6.118632434 | 1.26E-06 |
| KLK3 | NM_001030047 | 2.320310125 | 0.001065503 |
| KLK4 | NM_004917 | 1.767580055 | 1.45E-05 |
| KRT18 | NM_000224 | 1.012413234 | 0.000395054 |
| KRT19 | NM_002276 | 4.291577706 | 6.59E-08 |
|  | | | |
| Gene Name | **RefSeq ID** | **Log2FC** | **P value** |
| KRT6C | NM_173086 | 4.053386108 | 5.34E-05 |
| KRT8 | NM_002273 | 2.182815823 | 0.002754729 |
| LAMC1 | NM_002293 | 1.474425165 | 0.000576322 |
| LAT2 | NM_032464 | 3.176031122 | 0.000115541 |
| LGALS8 | L78132 | -1.373270919 | 0.006575802 |
| LIFR | NM_002310 | 2.992510409 | 5.23E-06 |
| LIG1 | NM_000234 | -1.553169352 | 0.000292004 |
| LMAN1 | NM_005570 | 1.530646173 | 0.004525011 |
| LONRF1 | NM_152271 | 2.240574604 | 4.52E-07 |
| LRBA | NM_006726 | -1.071278601 | 0.00043697 |
| LRRC31 | NM_024727 | -2.316398705 | 0.011969322 |
| LRRFIP2 | NM_006309 | 1.274071644 | 1.33E-05 |
| LRRN1 | NM_020873 | -4.007470179 | 3.64E-09 |
| LSS | NM_002340 | 1.073930326 | 0.000431496 |
| MAF | AF055376 | 4.781947608 | 1.87E-05 |
| MAK | NM_005906 | 3.280973762 | 1.01E-07 |
| MALT1 | NM_006785 | 2.307733081 | 2.71E-07 |
| MAN2B1 | NM_000528 | -1.424534231 | 0.004793775 |
| MANEA | NM_024641 | -3.838437618 | 8.84E-08 |
| MAP2K4 | NM_003010 | 1.363629486 | 3.90E-06 |
| MAP4K1 | NM_001042600 | -2.024461832 | 0.013233613 |
| MAPK6 | NM_002748 | 1.2206895 | 4.26E-05 |
| MAPK8 | NM_139047 | -1.417439361 | 0.033929144 |
| MAPKAPK3 | NM_004635 | -1.001347818 | 0.002604426 |
| MAPRE2 | ENST00000300249 | -3.500806982 | 5.86E-08 |
| MAPT | NM_016835 | -1.786529427 | 0.001401063 |
| MARS | NM_004990 | 1.425726522 | 0.002982872 |
| MATN2 | NM_030583 | -2.601201904 | 6.99E-07 |
| MCM7 | NM_005916 | -1.0401582 | 5.64E-05 |
| MDK | NM_001012334 | -1.205007349 | 3.06E-05 |
| MEF2A | NM_005587 | -1.000000000 | 0.000254661 |
| MERTK | NM_006343 | 2.772433947 | 2.79E-06 |
| MICAL1 | NM_022765 | 3.110932711 | 0.000313361 |
| MID1 | NM_000381 | -4.180144832 | 0.000204981 |
| MMP13 | NM_002427 | 3.126377014 | 0.004019329 |
| MPHOSPH9 | NM_022782 | 1.698590247 | 2.20E-05 |
| MPRIP | NM_015134 | -1.259722825 | 0.00248918 |
| MPZL1 | NM_024569 | 1.582200389 | 1.88E-06 |
| MRPS27 | NM_015084 | -1.010630993 | 0.000152656 |
| MTMR9 | NM_015458 | 2.023520052 | 7.89E-07 |
|  | | | |
| Gene Name | **RefSeq ID** | **Log2FC** | **P value** |
| MUC20 | NM_152673 | 1.146688524 | 0.000238612 |
| MXI1 | NM_005962 | -1.38832143 | 0.00053386 |
| MYB | NM_005375 | -3.996060691 | 2.42E-08 |
| MYC | NM_002467 | -1.034011624 | 0.00087796 |
| MYLK | NM_053027 | -3.67446696 | 1.88E-07 |
| MYO1B | NM_012223 | 1.224264255 | 6.64E-05 |
| MYOZ1 | NM_021245 | -2.725372014 | 1.34E-07 |
| NAIP | AK311046 | -1.989515065 | 0.002034244 |
| NAT1 | NM_000662 | 1.041931648 | 1.88E-05 |
| NBL1 | NM_182744 | 3.635434598 | 2.54E-06 |
| NCKAP1L | NM_005337 | 1.442216843 | 0.003681318 |
| NCOA2 | NM_006540 | -1.630946448 | 0.011968447 |
| NDRG1 | NM_006096 | 4.24579824 | 3.54E-09 |
| NEFL | NM_006158 | -1.125366415 | 0.000183698 |
| NET1 | NM_001047160 | -1.371365177 | 3.79E-06 |
| NFIA | NM_001134673 | -1.239835322 | 9.24E-05 |
| NFIX | NM_002501 | -1.101269107 | 0.005454665 |
| NFKB1 | NM_003998 | -1.307971854 | 7.26E-06 |
| NFKBIA | NM_020529 | 1.291024853 | 2.71E-06 |
| NIPSNAP3A | NM_015469 | -2.655251587 | 6.94E-06 |
| NKX3-1 | NM_006167 | 1.796300184 | 0.000197207 |
| NPAT | NM_002519 | -1.02781547 | 0.000202342 |
| NPC1 | NM_000271 | 1.973297395 | 5.24E-05 |
| NPPC | ENST00000409852 | 5.324052105 | 3.01E-09 |
| NR4A1 | D85245 | -1.338962813 | 0.015620518 |
| NUCB2 | AK097398 | -1.14572359 | 5.37E-06 |
| NUDT4 | NM_199040 | -1.016133115 | 0.002166384 |
| ODC1 | NM_002539 | 1.665031972 | 2.30E-06 |
| OPRK1 | NM_000912 | -6.573824477 | 1.48E-09 |
| ORM1 | NM_000607 | 5.967768888 | 4.89E-08 |
| OSR2 | NM_053001 | -2.258735997 | 1.18E-07 |
| OTUD4 | NM_199324 | -1.00052631 | 0.042661713 |
| PAK1IP1 | NM_017906 | 3.348011217 | 1.00E-06 |
| PAK2 | NM_002577 | -1.116487001 | 0.001181779 |
| PAM | NM_000919 | -1.211152531 | 0.000163725 |
| PCDH1 | NM_032420 | 1.601268834 | 0.001146567 |
| PCDH11X | NM_014522 | -4.129536673 | 0.000263895 |
| PCSK6 | NM_002570 | -1.999639175 | 0.003852617 |
| PCTP | NM_021213 | 1.03800735 | 0.001028057 |
| PDE4A | NM_006202 | -1.183568067 | 0.003585985 |
|  | | | |
| Gene Name | **RefSeq ID** | **Log2FC** | **P value** |
| PDE6A | NM_000440 | 3.118193293 | 2.71E-06 |
| PDE7A | NM_002604 | -1.849221484 | 0.002389911 |
| PDIA5 | NM_006810 | 1.544568313 | 1.61E-06 |
| PECI | NM_206836 | 1.646708944 | 3.62E-05 |
| PEG3 | NM_006210 | -3.387554166 | 2.40E-05 |
| PEPD | NM_000285 | -1.07620818 | 0.011195249 |
| PGC | NM_002630 | 8.179368455 | 4.04E-09 |
| PGM3 | NM_015599 | 1.887101088 | 9.18E-07 |
| PHF15 | AK025001 | -2.016728112 | 0.005237951 |
| PHF16 | NM_014735 | -1.570530297 | 0.000115708 |
| PHLDA2 | NM_003311 | 2.02860468 | 6.56E-07 |
| PHLDB2 | NM_145753 | 2.519768917 | 4.86E-05 |
| PIK3R1 | NM_181523 | -1.036612389 | 9.58E-05 |
| PKIB | NM_181795 | -1.745734044 | 1.92E-05 |
| PLCB4 | NM_001172646 | -1.977629547 | 8.16E-05 |
| PMAIP1 | NM_021127 | 1.330733937 | 0.003614499 |
| PMM2 | NM_000303 | 1.151830913 | 3.27E-05 |
| PNRC1 | NM_006813 | -1.061604188 | 0.022050398 |
| PPAP2A | NM_176895 | 1.891096037 | 1.60E-06 |
| PPFIBP1 | NM_003622 | 2.297990679 | 0.00016353 |
| PPIC | NM_000943 | 1.295139898 | 0.000112767 |
| PPM1K | NM_152542 | 1.049197589 | 0.000886114 |
| PPP1CB | NM_002709 | 1.034264176 | 6.60E-05 |
| PPP2CB | NM_001009552 | 1.024890969 | 0.000161842 |
| PRAME | NM_206956 | -1.557492788 | 0.011378159 |
| PRKAA1 | NM_206907 | 1.055789571 | 0.007679937 |
| PRKCA | NM_002737 | 1.048402861 | 0.000411411 |
| PRKD1 | NM_002742 | -1.768539993 | 1.97E-05 |
| PSMA6 | NM_002791 | 1.000099579 | 2.49E-05 |
| PSMD8 | NM_002812 | 1.04844738 | 1.54E-05 |
| PTCH1 | NM_000264 | -1.066116599 | 0.032680092 |
| PTEN | NM_000314 | -1.190595123 | 0.007818231 |
| PTPN11 | NM_002834 | 1.181168673 | 0.019308229 |
| PTPN21 | NM_007039 | 2.862588758 | 5.05E-08 |
| PTPRM | NM_002845 | 2.769376818 | 0.001489229 |
| PTPRR | NM_002849 | -2.506458666 | 0.000231438 |
| PURA | NM_005859 | -1.400042108 | 8.67E-05 |
| RAB27A | NM_004580 | 2.201882535 | 1.12E-06 |
| RAB4A | NM_004578 | 1.236048228 | 4.21E-06 |
| RAB6A | BC044241 | 2.18284584 | 3.87E-07 |
|  | | | |
| Gene Name | **RefSeq ID** | **Log2FC** | **P value** |
| RAD9A | NM_004584 | -1.425442751 | 0.044235906 |
| RALB | NM_002881 | -1.057718811 | 0.000131306 |
| RBM6 | NM_005777 | -1.002217654 | 7.05E-05 |
| RCAN1 | NM_004414 | 1.389577043 | 3.89E-06 |
| RELN | NM_005045 | -2.329987476 | 0.002201479 |
| RHOU | NM_021205 | 2.56431912 | 1.75E-06 |
| RLN2 | NM_005059 | -2.217478829 | 2.25E-06 |
| RNASE4 | NM_002937 | 1.390870467 | 2.31E-05 |
| ROR1 | ENST00000371079 | 1.859613208 | 2.13E-05 |
| RPS6KA1 | NM_002953 | -1.040260779 | 0.030198237 |
| RPS6KA3 | NM_004586 | 1.245190436 | 0.000110762 |
| RUNX2 | NM_004348 | 2.471438096 | 5.90E-05 |
| S100A11 | NM_005620 | 1.196033674 | 3.65E-05 |
| SAP18 | NM_005870 | -1.127904536 | 0.000653415 |
| SASH1 | NM_015278 | 1.657827243 | 0.049529458 |
| SCN1B | NM_199037 | 2.059040982 | 0.007178679 |
| SCNN1A | NM_001038 | -1.487811278 | 0.040905824 |
| SDC4 | NM_002999 | -1.601322138 | 0.000391347 |
| SDHA | NM_004168 | -1.254381876 | 0.021648136 |
| SDK1 | NM_152744 | 3.36191812 | 0.011245931 |
| SEC24A | BC019341 | 1.153927142 | 0.0038223 |
| SEC24D | NM_014822 | 1.841360757 | 0.000143376 |
| SEC61B | NM_006808 | 1.042916443 | 5.94E-05 |
| SELENBP1 | NM_003944 | -2.607288857 | 3.50E-06 |
| SEMA5A | NM_003966 | -2.53121744 | 0.000583286 |
| SEPP1 | NM_005410 | 1.338926967 | 8.12E-06 |
| SERP1 | NM_014445 | 1.084997232 | 4.04E-05 |
| SERPINB10 | NM_005024 | 1.293613685 | 0.042516088 |
| SERPINB5 | NM_002639 | -1.717528718 | 3.28E-05 |
| SERPINI1 | NM_005025 | -3.703004865 | 3.07E-07 |
| SGK1 | NM_005627 | 1.471824887 | 1.17E-05 |
| SI | NM_001041 | -6.033603824 | 2.15E-06 |
| SLC12A1 | NM_000338 | 1.18698921 | 0.011572959 |
| SLC15A2 | NM_021082 | 3.68815419 | 0.000869645 |
| SLC16A6 | NM_004694 | 3.042867402 | 6.45E-08 |
| SLC22A1 | NM_153187 | 1.213023611 | 9.50E-05 |
| SLC26A2 | NM_000112 | 1.1138732 | 0.008130955 |
| SLC31A2 | NM_001860 | 1.396377947 | 9.20E-06 |
| SLC38A2 | NM_018976 | 1.029259168 | 0.000234869 |
| SLCO1B3 | NM_019844 | 1.293270757 | 0.025508542 |
|  | | | |
| Gene Name | **RefSeq ID** | **Log2FC** | **P value** |
| SMAD6 | NM_005585 | -1.896779959 | 0.003075481 |
| SMAD7 | NM_005904 | -1.457380769 | 2.86E-06 |
| SMARCC2 | NM_139067 | -1.291139109 | 0.006539983 |
| SMARCD3 | NM_003078 | -2.81924027 | 1.71E-07 |
| SMS | NM_004595 | 2.454788777 | 4.56E-07 |
| SNAP25 | NM_003081 | -2.391316102 | 0.01016724 |
| SNRK | NM_017719 | -1.413351229 | 4.63E-05 |
| SNTA1 | NM_003098 | -1.321403179 | 0.006989342 |
| SNX25 | NM_031953 | 1.440699814 | 1.59E-06 |
| SOCS2 | NM_003877 | 2.790084682 | 9.83E-07 |
| SORD | NM_003104 | 1.681422027 | 6.32E-05 |
| SOX4 | NM_003107 | -1.133258637 | 1.96E-05 |
| SPDEF | NM_012391 | 1.204458996 | 0.003653186 |
| SREBF1 | NM_001005291 | -1.085077096 | 0.009967103 |
| SRP19 | NM_003135 | 1.532528736 | 0.000327893 |
| SSBP2 | ENST00000320672 | -2.93587417 | 1.36E-07 |
| SSR1 | NM_003144 | 1.268576242 | 9.16E-06 |
| SSR3 | NM_007107 | 1.844351289 | 5.11E-05 |
| ST13 | NM_003932 | 1.046656524 | 0.000778757 |
| ST3GAL1 | NM_003033 | -1.042296428 | 0.000140729 |
| ST6GALNAC1 | NM_018414 | 6.153320289 | 8.75E-09 |
| ST7 | NM_018412 | -3.60121681 | 3.08E-07 |
| STK17B | ENST00000263955 | 2.450044492 | 8.60E-07 |
| STK39 | NM_013233 | 3.048350447 | 3.61E-06 |
| STXBP1 | NM_003165 | -1.306348349 | 0.005217897 |
| SYNJ1 | NM_203446 | 1.342421447 | 0.003548156 |
| SYTL2 | NM_032943 | -2.037397797 | 0.005323263 |
| TARP | NM_001003799 | 2.276292899 | 0.0006213 |
| TBC1D1 | NM_015173 | 1.186376436 | 7.24E-05 |
| TBRG1 | NM_032811 | 1.322944628 | 1.08E-05 |
| TCL1A | NM_021966 | -1.48365262 | 0.00817251 |
| TFPI | NM_006287 | -1.022641236 | 0.00052928 |
| THRA | NM_003250 | -2.482719819 | 3.80E-06 |
| TLE1 | CR612105 | 1.026692517 | 0.003240275 |
| TMPRSS2 | NM_005656 | 2.763944983 | 1.28E-06 |
| TNFAIP3 | NM_006290 | 3.135577346 | 1.64E-06 |
| TNFAIP8 | NM_014350 | 1.634536169 | 7.20E-06 |
| TNRC6B | NM_015088 | -1.287027334 | 0.00026497 |
| TPD52 | NM_001025252 | 1.046253429 | 0.000282122 |
| TRIM35 | NM_171982 | -1.667580485 | 0.026954093 |
|  | | | |
| Gene Name | **RefSeq ID** | **Log2FC** | **P value** |
| TRIM36 | NM_018700 | 1.185113219 | 1.44E-05 |
| TRIM45 | NM_025188 | -2.449267061 | 1.45E-07 |
| TRPS1 | NM_014112 | -2.790446992 | 6.69E-08 |
| TWIST1 | NM_000474 | 1.057628538 | 5.95E-05 |
| TXNIP | NM_006472 | -2.016017087 | 1.08E-05 |
| UAP1 | NM_003115 | 1.571964099 | 1.30E-05 |
| UBE2B | BC001694 | -1.522456144 | 0.013902659 |
| UBE2V1 | NM_001032288 | 1.059950238 | 0.000941137 |
| UBE2V2 | NM_003350 | 1.16169537 | 0.009466369 |
| UGT2B15 | NM_001076 | -6.182288324 | 2.50E-08 |
| VCL | NM_014000 | 1.043092908 | 0.001033197 |
| WASF3 | NM_006646 | -1.139624727 | 0.020889696 |
| WWTR1 | NM_015472 | 3.490604954 | 1.68E-07 |
| XPA | NM_000380 | -1.125545059 | 0.000117956 |
| XPC | NM_004628 | -1.245031085 | 0.003188688 |
| ZIC2 | NM_007129 | 1.215944097 | 0.042301954 |
| ZNF133 | NM_003434 | -1.077957523 | 6.78E-05 |
| ZNF174 | AF542096 | -1.722461681 | 0.029286019 |
| ZNF76 | NM_003427 | -1.187623113 | 0.033223379 |

**Supplementary File 1C.** Coregulators considered, prioritized and withheld for analysis

|  | | | |
| --- | --- | --- | --- |
| Coregulator | **Differential CaP protein expression?** | **Δ expression relevant to  CaP progression?** | **Withheld for oligoarray analysis?** |
| AATF | no | no | no |
| ACTN2 | no | no | no |
| ACTN4 | yes | no | no |
| AES | no | no | no |
| ANP32A | no | no | no |
| APPBP2 | no | no | no |
| APPL1 | no | no | no |
| ARHGDIA | no | no | no |
| ARID1A | no | no | no |
| ARID1B | no | no | no |
| ARID5A | no | no | no |
| ARRB2 | no | no | no |
| ATAD2 | no | no | no |
| BAG1 | yes | yes | yes |
| BRCA1 | no | no | no |
| BRCA2 | no | no | no |
| CALCOCO1 | no | no | no |
| CARM1 | yes | no | no |
| CASP8 | no | no | no |
| CAV1 | yes | yes | yes |
| CBX1 | no | no | no |
| CCND1 | yes | no | no |
| CCNE1 | no | no | no |
| CDC25A | no | no | no |
| CDC25B | yes | no | no |
| CDC37 | yes | no | no |
| CDK6 | no | no | no |
| CHD8 | no | no | no |
| COPS2 | no | no | no |
| CREBBP | yes | no | no |
| CTDSP2 | no | no | no |
| CTNNB1 | yes | yes | yes |
| DCAF6 | no | no | no |
| DDC | yes | no | no |
| DDX17 | no | no | no |
| DDX5 | no | no | no |
| DNAJA1 | no | no | no |
| DNAJB1 | no | no | no |
|  |  |  |  |
| Coregulator | **Differential CaP protein expression?** | **Δ expression relevant to  CaP progression?** | **Withheld for oligoarray analysis?** |
| DYRK1A | no | no | no |
| EFCAB6 | no | no | no |
| EHMT2 | no | no | no |
| EP300 | yes | yes | yes |
| ETV1 | no | no | no |
| FHL2 | yes | yes | yes |
| FKBP4 | no | no | no |
| FKBP5 | no | no | no |
| FKBPL | no | no | no |
| FLNA | no | no | no |
| GAK | yes | yes | no |
| GLI2 | no | no | no |
| GNB2L1 | no | no | no |
| GSK3B | no | no | no |
| GSN | yes | no | no |
| HDAC7 | no | no | no |
| HEY1 | yes | no | no |
| HEY2 | no | no | no |
| HIP1 | yes | yes | no |
| HIPK3 | no | no | no |
| HNRNPA1 | no | no | no |
| HSP90AA1 | yes | no | no |
| HSPA4 | no | no | no |
| HTATIP2 | yes | yes | yes |
| JUND | no | no | no |
| KAT2B | no | no | no |
| KAT5 | yes | yes | yes |
| KDM1A | yes | yes | yes |
| KDM3A | no | no | no |
| KDM4C | no | no | no |
| KDM5B | yes | no | no |
| KHDRBS1 | yes | no | no |
| LATS2 | yes | no | no |
| MACROD1 | no | no | no |
| MAGEA11 | no | no | no |
| MAK | no | no | no |
| MAPK15 | no | no | no |
| MDM2 | no | no | no |
| MED1 | yes | no | no |
| Coregulator | **Differential CaP protein expression?** | **Δ expression relevant to  CaP progression?** | **Withheld for oligoarray analysis?** |
| MKRN1 | no | no | no |
| MYST2 | no | no | no |
| NCOA1 | yes | yes | yes |
| NCOA2 | yes | yes | yes |
| NCOA3 | yes | yes | yes |
| NCOA4 | yes | no | no |
| NCOA6 | no | no | no |
| NCOR1 | no | no | no |
| NCOR2 | no | no | no |
| NELFCD | no | no | no |
| NONO | no | no | no |
| NRIP1 | no | no | no |
| NSD1 | no | no | no |
| PA2G4 | no | no | no |
| PAK6 | yes | no | no |
| PARK7 | yes | yes | yes |
| PATZ1 | no | no | no |
| PAWR | yes | no | no |
| PELP1 | yes | no | no |
| PIAS1 | no | no | no |
| PIAS2 | no | no | no |
| PIAS3 | yes | no | no |
| PIAS4 | no | no | no |
| PKN1 | yes | yes | yes |
| PLAGL1 | no | no | no |
| PNRC1 | no | no | no |
| POU4F1 | no | no | no |
| PPARGC1A | no | no | no |
| PPP1CC | no | no | no |
| PPP2R4 | no | no | no |
| PRDX1 | no | no | no |
| PRIC285 | no | no | no |
| PRKDC | no | no | no |
| PRMT1 | no | no | no |
| PRPF6 | no | no | no |
| PSIP1 | no | no | no |
| PSMC3 | no | no | no |
| PSMC3IP | no | no | no |
| PSPC1 | no | no | no |
| PTEN | no | no | no |
| Coregulator | **Differential CaP protein expression?** | **Δ expression relevant to  CaP progression?** | **Withheld for oligoarray analysis?** |
| PXN | no | no | no |
| RAD54L2 | no | no | no |
| RAD9A | yes | yes | no |
| RAN | no | no | no |
| RANBP9 | no | no | no |
| RB1 | no | no | no |
| RBAK | no | no | no |
| RBM14 | no | no | no |
| RCHY1 | yes | yes | yes |
| RNASEL | no | no | no |
| RNF14 | no | no | no |
| RNF4 | no | no | no |
| RNF6 | no | no | no |
| RPS6KA1 | no | no | no |
| SART3 | no | no | no |
| SENP1 | yes | no | no |
| SFPQ | no | no | no |
| SFRP1 | no | no | no |
| SGTA | yes | no | no |
| SIRT1 | yes | no | no |
| SMAD3 | yes | yes | no |
| SMARCA2 | no | no | no |
| SMARCA4 | yes | yes | yes |
| SMARCC1 | yes | yes | yes |
| SMARCD1 | no | no | no |
| SMARCE1 | yes | no | no |
| SORBS3 | no | no | no |
| SRA1 | no | no | no |
| SRCAP | no | no | no |
| STAT3 | yes | yes | yes |
| STUB1 | no | no | no |
| SUMO1 | no | no | no |
| SUMO2 | no | no | no |
| SUMO3 | no | no | no |
| SVIL | no | no | no |
| TAF1 | no | no | no |
| TAGLN | no | no | no |
| TCF4 | no | no | no |
| TGFB1I1 | yes | no | no |
| TGIF1 | no | no | no |
| Coregulator | **Differential CaP protein expression?** | **Δ expression relevant to  CaP progression?** | **Withheld for oligoarray analysis?** |
| TMF1 | no | no | no |
| TNK2 | no | no | no |
| TOB1 | no | no | no |
| TOB2 | no | no | no |
| TRIM24 | no | no | no |
| TRIM68 | yes | no | no |
| TRIP4 | no | no | no |
| TSG101 | no | no | no |
| UBA3 | no | no | no |
| UBE2I | no | no | no |
| UBE2L3 | no | no | no |
| UBE3A | yes | no | no |
| USP10 | no | no | no |
| USP26 | no | no | no |
| UXT | yes | no | no |
| VAV3 | yes | no | no |
| WDR77 | yes | yes | yes |
| WHSC1 | no | no | no |
| XRCC5 | no | no | no |
| XRCC6 | no | no | no |
| YY1 | no | no | no |
| ZMIZ1 | no | no | no |
| ZMIZ2 | no | no | no |
| ZNF318 | no | no | no |

**Supplementary File 1. Design of oligoarray, overview of AR target genes studied, and overview of coregulators considered for analysis.**

**A. Overview of genes included in custom Agilent oligoarray**

Rows, categories of genes included on 8x15K custom Agilent oligoarray. Columns, Number of genes identified for inclusion on the array, and number of genes for which Agilent catalogue probes were available for inclusion.

**B. Overview of 452 AR target gene signature**

Gene name, HUGO gene symbol ; FC, fold change

**C. Overview of coregulators considered, prioritized and withheld for analysis**

A PudMed search for papers that contain the terms “AR” and “CaP” in their title and/or abstract was performed. Abstracts fulfilling these criteria were screened for reference to coregulator function, and if so, full-length papers were reviewed individually to verify description of a *bona fide* AR-associated coregulator.

Left to right: **Column 1**: 181 coregulators for which literature search was done. **Column 2**: 51 coregulators for which differential protein expression has been reported in CaP when compared to benign prostate (yes entries). **Column 3**: 22 coregulators for which differential expression in CaP correlated with aggressive disease, and were analyzed in Figures 4-6 (yes entries). **Column 4**: 18 coregulators for which siRNA-mediated silencing did not affect AR expression, CaP cell morphology or CaP cell survival and were included in final analyses (yes entries).
