# Supplementary material for: A comprehensive analysis of coregulator recruitment, androgen receptor function and gene expression in prostate cancer
Source: eLife. 2017 Aug 18;6:e28482. doi: 10.7554/eLife.28482 (PMC5608510; doi:10.7554/eLife.28482)
Supplement: Supplementary file 2. — (A) Androgen regulation of AR target gene expression in VCaP cells VCaP cells were seeded in medium supplemented with charcoal-stripped FBS (CSS). 2 days later, medium was changed and cells were treated with 5 nm R1881 or ethanol vehicle for 48 hr. Cells were harvested and AR target gene expression was evaluated using real-time RT-PCR. Target gene mRNA levels were normalized with the values obtained from GAPDH expression and are expressed as relative expression values, taking the value obtained from one of the vehicle-treated samples as 1. Columns, means of values obtained from three independent biological replicates; bars, sem. Note the consistency of androgen regulation of majority (7/8) of AR target genes obtained from LNCaP cells also in VCaP cells. (B) Kinetics of androgen regulation of AR target gene expression LNCaP cells were seeded in medium supplemented with charcoal-stripped FBS (CSS). 2 days later, medium was changed and cells were with 5 nm R1881 or ethanol for 4 or 8 hr. Timepoints were chosen as androgen regulation of androgen-induced AR target genes is detectable at 4 hr, and androgen regulation of androgen-repressed AR target genes becomes apparent at 8 hr. Target gene mRNA levels were normalized with the values obtained from GAPDH expression and are expressed as relative expression values, taking the value obtained from one of the vehicle-treated samples at the 4 hr time point as 1. Columns, means of values obtained from three independent biological replicates; bars, sem values. Note the consistency in kinetics of androgen regulation of newly recognized AR target genes (bottom 2 rows) with that of the well-known ARE-driven genes PSA, SCAP, FN1 and SERPINB5 (top row). (C) Representative real-time RT-PCR validation of Agilent oligoarray data Side-by-side comparison of Agilent oligoarray data and real-time RT-PCR data using same RNA samples. Expression data shown are derived from genes for which Agilent oligoarray expression was low (Y axis values, to [file elife-28482-supp2.pdf]

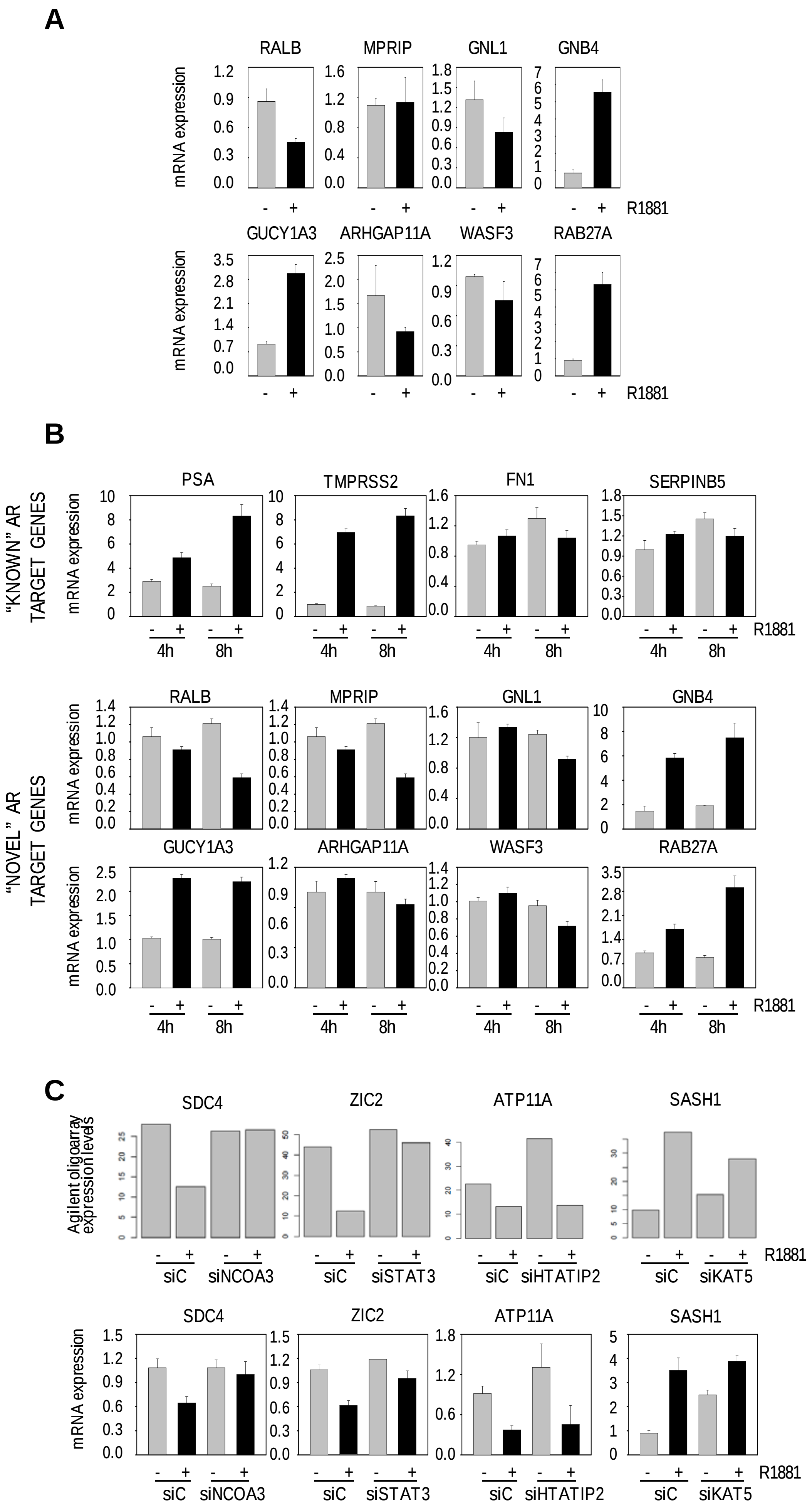

Supplementary File 2

Supplementary File 2. Characterization of 452 AR target gene signature

A.Androgen regulation of AR target gene expression in VCaP cells

VCaP cells were seeded in medium supplemented with charcoal-stripped FBS (CSS). 2 days later, medium was changed and cells were treated with 5 nm R1881 or ethanol vehicle for 48 h. Cells were harvested and AR target gene expression was evaluated using real-time RT-PCR. Target gene mRNA levels were normalized with the values obtained from GAPDH expression and are expressed as relative expression values, taking the value obtained from one of the vehicle-treated samples as 1. *Columns*, means of values obtained from three independent biological replicates; *bars*, sem.

Note the consistency of androgen regulation of majority (7/8) of AR target genes obtained from LNCaP cells also in VCaP cells.

B. Kinetics of androgen regulation of AR target gene expression

LNCaP cells were seeded in medium supplemented with charcoal-stripped FBS (CSS). 2 days later, medium was changed and cells were with 5 nm R1881 or ethanol for 4 or 8 h. Timepoints were chosen as androgen regulation of androgen-induced AR target genes is detectable at 4h, and androgen regulation of androgen-repressed AR target genes becomes apparent at 8h. Target gene mRNA levels were normalized with the values obtained from GAPDH expression and are expressed as relative expression values, taking the value obtained from one of the vehicle-treated samples at the 4h time point as 1. *Columns*, means of values obtained from three independent biological replicates; *bars*, sem values.

Note the consistency in kinetics of androgen regulation of newly recognized AR target genes (bottom 2 rows) with that of the well-known ARE-driven genes PSA, SCAP, FN1 and SERPINB5 (top row).

C. Representative real-time RT-PCR validation of Agilent oligoarray data

Side-by-side comparison of Agilent oligoarray data and real-time RT-PCR data using same RNA samples. Expression data shown are derived from genes for which Agilent oligoarray expression was low (Y axis values, top row), and thus likely less reliable.
