# Supplementary material for: A comprehensive analysis of coregulator recruitment, androgen receptor function and gene expression in prostate cancer
Source: eLife. 2017 Aug 18;6:e28482. doi: 10.7554/eLife.28482 (PMC5608510; doi:10.7554/eLife.28482)
Supplement: Supplementary file 3. [file elife-28482-supp3.docx]

**Supplementary File 3. Ingenuity Pathway Analyses on diverse AR-dependent gene sets**

| **Supplementary File 3A. Ingenuity Pathway Analysis for cell functions associated with the 452 AR target gene signature** | | | | |
| --- | --- | --- | --- | --- |
| **Function Category** | **Function Annotation** | **P-value** | **Predicted Activation State** | **Regulation**  **z-score** |
| Cancer | Cancer | 8.68E-26 | Increased | 3.101 |
| Cancer | Tumorigenesis | 1.24E-25 | Increased | 2.472 |
| Cancer | Neoplasia | 1.76E-25 | Increased | 2.997 |
| Cancer | solid tumor | 9.62E-20 | Increased | 2.409 |
| Cancer | Carcinoma | 3.74E-19 | Increased | 2.120 |
| Cellular growth and proliferation | proliferation of prostate cancer cell lines | 9.87E-08 | Increased | 2.019 |
| Cellular development | proliferation of prostate cancer cell lines | 9.87E-08 | Increased | 2.019 |
| Cancer | tumorigenesis of squamous-cell carcinoma | 9.87E-07 | Increased | 2.449 |
| Cancer | tumorigenesis of cells | 8.77E-06 | Increased | 2.139 |
| Cellular development | differentiation of lymphocytes | 1.68E-05 | Decreased | -2.023 |
| Hematological system development   and function | differentiation of lymphocytes | 1.68E-05 | Decreased | -2.023 |
| Hematopoiesis | differentiation of lymphocytes | 1.68E-05 | Decreased | -2.023 |
| Cancer | Hyperplasia | 1.74E-05 | Increased | 2.125 |
| Cellular movement | Infiltration | 1.77E-05 | Increased | 2.015 |
| Cell death | cell death of hematopoietic cell lines | 2.67E-05 | Increased | 2.571 |
| Cell morphology | reorganization of cytoskeleton | 3.07E-05 | Increased | 2.105 |
| Cellular function and maintenance | reorganization of cytoskeleton | 3.07E-05 | Increased | 2.105 |
| Cellular assembly and organization | reorganization of cytoskeleton | 3.07E-05 | Increased | 2.105 |
| Cell Death | apoptosis of hematopoietic cell lines | 3.14E-05 | Increased | 2.164 |
| Cellular movement | infiltration of leukocytes | 3.47E-05 | Increased | 2.039 |
| Hematological system development   and function | infiltration of leukocytes | 3.47E-05 | Increased | 2.039 |
| Immune cell trafficking | infiltration of leukocytes | 3.47E-05 | Increased | 2.039 |
| Cancer | tumorigenesis of malignant tumor | 3.69E-05 | Increased | 2.536 |
| Cellular movement | infiltration of cells | 3.69E-05 | Increased | 2.015 |
| Molecular transport | transport of molecule | 3.83E-05 | Increased | 2.051 |
| Cell death | cell death of leukocyte cell lines | 5.12E-05 | Increased | 2.415 |
| **Supplementary File 3A, con’t** |  |  |  |  |
| **Function Category** | **Function Annotation** | **P-value** | **Predicted Activation State** | **Regulation**  **z-score** |
| Lipid metabolism | transport of fatty acid | 6.08E-05 | Increased | 2.177 |
| Small molecule biochemistry | transport of fatty acid | 6.08E-05 | Increased | 2.177 |
| Molecular transport | transport of fatty acid | 6.08E-05 | Increased | 2.177 |
| Cell death | apoptosis of ovarian cancer cell lines | 6.90E-05 | Decreased | -2.216 |
| Cancer | tumorigenesis of carcinoma | 7.60E-05 | Increased | 2.038 |
| Cell death | cell death of B-lymphocyte derived cell lines | 7.73E-05 | Increased | 2.060 |
| Cell death | cell death of liver cells | 8.60E-05 | Decreased | -2.497 |
| Cell death | apoptosis of liver cells | 2.54E-04 | Decreased | -3.032 |
| Cancer | tumorigenesis of skin | 2.65E-04 | Increased | 2.059 |
| Dermatological diseases and conditions | tumorigenesis of skin | 2.65E-04 | Increased | 2.059 |
|  |  |  |  |  |
|  |  |  |  |  |
| **Ingenuity Canonical Pathways** | **-log(p-value)** | **Ratio** |  |  |
| TGF-β Signaling | 5.49E00 | 1.35E-01 |  |  |
| BMP signaling pathway | 5.32E00 | 1.38E-01 |  |  |
| NRF2-mediated oxidative stress response | 4.93E00 | 8.9E-02 |  |  |
| Aryl hydrocarbon receptor signaling | 4.51E00 | 8.81E-02 |  |  |
| Inositol phosphate metabolism | 4.51E00 | 7.58E-02 |  |  |
| Molecular mechanisms of cancer | 4.05E00 | 6.08E-02 |  |  |
| Role of IL-17A in arthritis | 3.92E00 | 1.27E-01 |  |  |
| TNFR2 signaling | 3.89E00 | 1.76E-01 |  |  |
| Propanoate metabolism | 3.86E00 | 6.61E-02 |  |  |

| **Supplementary File 3B.** Ingenuity Pathway Analysis categories associated with coregulator-dependent AR target gene sets | | | | | | | | |
| --- | --- | --- | --- | --- | --- | --- | --- | --- |
| **Gene** | **Category** | **Functions Annotation** | **p-Value** | **Predicted Activation State** | **Regulation z-score** | **Genes** | **Number of genes** |  |
| **SMARCC1** | Cell Death | cell viability | 1.27E-05 | Increased | 2.052 | ANTXR1,CYR61,FN1,GCG,GUCY1A3,ITGAV,KLF5,KRT19 (human),MALT1,MYC,NDRG1,NFKBIA,PPAP2A,PRKCA,PTPRM, RCAN1,SERPINI1,SGK1,SOCS2,TNFAIP3,TWIST1,TXNIP | 22 |  |
|  | Organismal Survival | organismal death | 1.96E-05 | Decreased | -2.608 | AKAP12,ANKH,ATP1B1,CREB3L2,CYR61,DNAJC3,FN1,GCG, GUCY1A3,INHBB,INSIG1,IQGAP2,ITGAV,KLF4,MAF,MERTK,MXI1,MYC,NDRG1,NFKBIA,NPC1,PRKCA,RUNX2,SEPP1,SSBP2,TNFAIP3,TXNIP | 27 |  |
|  | Cell Death | cell death of tumor cell lines | 4.14E-05 | Decreased | -2.045 | AKAP12,ANXA2,ATF3,BMPR1B,BTG2,CDC6 (includes EG:23834),CREB3L2,CTBP1,CYR61,FN1,ITGAV,KLF4,KLF5,KLK3, MYC,NDRG1,NFKBIA,PRKCA,SERPINI1,SGK1,TNFAIP3,TRPS1, TWIST1 | 23 |  |
|  | Gene Expression | binding of DNA | 3.79E-04 | Decreased | -2.080 | BMPR1B,BTG2,FN1,GCG,MYC,NFKBIA,PRKCA,RPS6KA3,RUNX2,SOCS2,SSBP2,TNFAIP3 | 12 |  |
|  | Cell Death | apoptosis of liver cells | 2.50E-03 | Decreased | -2.000 | IQGAP2,ITGAV,MYC,NPC1,SEPP1 | 5 |  |
|  |  |  |  |  |  |  |  |  |
| **SMARCA4** | Cancer | cancer | 1.08E-11 | Increased | 2.294 | ABCC4 (includes EG:10257),ABI2,ACTG2,ADARB1,AGR2,ALDH1A3,ANTXR1,ANXA2,AR,ASRGL1,ATF3,ATP1B1,B2M,C16orf45,CALD1,CDC6 (includes EG:23834),CLDN12,COLEC12,CTBP1,CXCR7,CYP3A7,DDB2,DNAJB9,DNM1L,EFCAB11,EPS8,F2RL1,FN1,GALK2,GREB1,GTF2I,HOXB13,IGF1,IL1RN,IQGAP2,ITGAV,KLF4,KRT18,KRT19 (human),LIG1,MAF,MATN2,MCM7,MYLK,NFKBIA,NPC1,ODC1,OPRK1,OSR2,PAK1IP1,PHLDA2,PIK3R1,PLCB4,PRAME,PRKCA,PSMA6,PTEN,RELN,ROR1,RUNX2,SELENBP1,SGK1,SMS,SSBP2,STK17B,TARP,TFPI,TNFAIP3,TPD52,TWIST1 | 70 |  |
|  | Cancer | tumorigenesis | 1.59E-10 | Increased | 2.435 | ABCC4 (includes EG:10257),ABI2,ACTG2,ADARB1,AGR2,ALDH1A3 ,ANTXR1,ANXA2,AR,ASRGL1,ATF3,ATP11A,ATP1B1,B2M,C16orf45,CALD1,CDC6 (includes EG:23834),CLDN12,COLEC12,CTBP1, CXADR,CXCR7,CYP3A7,DDB2,DNAJB9,DNM1L,EFCAB11,EPS8,F2RL1,FN1,GALK2,GCG,GREB1,GTF2I,HOXB13,IGF1,IL1RN,IQGAP2,ITGAV,KLF4,KRT18,KRT19 (human),LIG1,MAF,MATN2,MCM7, MYLK,NFKBIA,NPC1,ODC1,OPRK1,OSR2,PAK1IP1,PHLDA2,PIK3R1,PLCB4,PRAME,PRKCA,PSMA6,PTEN,RELN,ROR1,RUNX2,SELENBP1,SGK1,SMS,SSBP2,STK17B,TARP,TFPI,TNFAIP3,TPD52,TWIST1 | 73 |  |
|  | Cancer | metastasis | 3.01E-06 | Increased | 2.273 | ATF3,COLEC12,CXCR7,DDB2,FN1,GREB1,IGF1,IL1RN,ITGAV,KRT18,NFKBIA,PIK3R1,PTEN,RELN,RUNX2,TFPI,TWIST1 | 17 |  |
|  | Cellular Assembly, Organization | organization of cytoplasm | 6.87E-06 | Increased | 2.103 | ABI2,ANTXR1,AR,ATF3,CTBP1,CXADR,DNM1L,EPS8,F2RL1,FN1,GALK2,IGF1,ITGAV,KRT18,LMAN1,MATN2,MID1 (includes EG:100330952),NBL1 (includes EG:17965),NFKBIA,NPC1,PIK3R1, PRKCA,PTEN,RELN,RHOU,ROR1,SGK1,TWIST1 | 28 |  |
|  | Cellular Function, Maintenance | organization of cytoplasm | 6.87E-06 | Increased | 2.103 | ABI2,ANTXR1,AR,ATF3,CTBP1,CXADR,DNM1L,EPS8,F2RL1,FN1,GALK2,IGF1,ITGAV,KRT18,LMAN1,MATN2,MID1 (includes EG:100330952),NBL1 (includes EG:17965),NFKBIA,NPC1,PIK3R1, PRKCA,PTEN,RELN,RHOU,ROR1,SGK1,TWIST1 | 28 |  |
|  | Cellular Assembly, Organization | organization of cytoskeleton | 8.59E-06 | Increased | 2.103 | ABI2,ANTXR1,AR,ATF3,CXADR,DNM1L,EPS8,F2RL1,FN1,GALK2,IGF1,ITGAV,KRT18,MATN2,MID1 (includes EG:100330952),NBL1 (includes EG:17965),NFKBIA,NPC1,PIK3R1,PRKCA,PTEN,RELN, RHOU,ROR1,SGK1,TWIST1 | 26 |  |
|  | Cellular Function, Maintenance | organization of cytoskeleton | 8.59E-06 | Increased | 2.103 | ABI2,ANTXR1,AR,ATF3,CXADR,DNM1L,EPS8,F2RL1,FN1,GALK2, IGF1,ITGAV,KRT18,MATN2,MID1 (includes EG:100330952),NBL1 (includes EG:17965),NFKBIA,NPC1,PIK3R1,PRKCA,PTEN,RELN, RHOU,ROR1,SGK1,TWIST1 | 26 |  |
|  | Cancer | tumorigenesis, of cells | 1.93E-05 | Increased | 2.122 | ATF3,CXADR,FN1,GCG,IGF1,ITGAV,NFKBIA,ODC1,PTEN,RELN,ROR1,RUNX2,TPD52,TWIST1 | 14 |  |
|  | Cancer | tumorigenesis of carcinoma | 3.85E-05 | Increased | 2.195 | B2M,DDB2,IGF1,IQGAP2,NFKBIA,ODC1,PIK3R1,PTEN, SSBP2 | 9 |  |
|  | Cancer | tumorigenesis of malignant tumor | 2.23E-04 | Increased | 2.432 | B2M,DDB2,IGF1,IL1RN,IQGAP2,NFKBIA,ODC1,PIK3R1,PTEN, SSBP2 | 10 |  |
|  | Organismal Development | development of blood vessel | 1.03E-03 | Increased | 2.115 | ANXA2,ATF3,F2RL1,FN1,GTF2I,IGF1,ITGAV,NPPC,ODC1,PIK3R1, PRKCA,PTEN,ROR1,RUNX2,TFPI,TNFAIP3 | 16 |  |
|  | Cardiovascular Development, Function | development of blood vessel | 1.03E-03 | Increased | 2.115 | ANXA2,ATF3,F2RL1,FN1,GTF2I,IGF1,ITGAV,NPPC,ODC1,PIK3R1, PRKCA,PTEN,ROR1,RUNX2,TFPI,TNFAIP3 | 16 |  |
|  | Cellular Development | endothelial cell development | 1.17E-03 | Increased | 2.317 | F2RL1,FN1,IGF1,NPPC,ODC1,PRKCA,PTEN,RUNX2,TFPI | 9 |  |
|  | Organismal Development | endothelial cell development | 1.17E-03 | Increased | 2.317 | F2RL1,FN1,IGF1,NPPC,ODC1,PRKCA,PTEN,RUNX2,TFPI | 9 |  |
|  | Tissue Development | endothelial cell development | 1.17E-03 | Increased | 2.317 | F2RL1,FN1,IGF1,NPPC,ODC1,PRKCA,PTEN,RUNX2,TFPI | 9 |  |
|  | Cardiovascular Development, Function | endothelial cell development | 1.17E-03 | Increased | 2.317 | F2RL1,FN1,IGF1,NPPC,ODC1,PRKCA,PTEN,RUNX2,TFPI | 9 |  |
|  | Hematological Disease | hypoglycemia | 1.72E-03 | Increased | 2.147 | ATF3,GCG,IGF1,IL1RN,PIK3R1 | 5 |  |
|  | Endocrine System Disorders | hypoglycemia | 1.72E-03 | Increased | 2.147 | ATF3,GCG,IGF1,IL1RN,PIK3R1 | 5 |  |
|  | Metabolic Disease | hypoglycemia | 1.72E-03 | Increased | 2.147 | ATF3,GCG,IGF1,IL1RN,PIK3R1 | 5 |  |
|  | Cellular Development | proliferation of endothelial cells | 1.74E-03 | Increased | 2.040 | F2RL1,FN1,IGF1,NPPC,PRKCA,PTEN,RUNX2,TFPI | 8 |  |
|  | Cellular Growth and Proliferation | proliferation of endothelial cells | 1.74E-03 | Increased | 2.040 | F2RL1,FN1,IGF1,NPPC,PRKCA,PTEN,RUNX2,TFPI | 8 |  |
|  | Organismal Development | proliferation of endothelial cells | 1.74E-03 | Increased | 2.040 | F2RL1,FN1,IGF1,NPPC,PRKCA,PTEN,RUNX2,TFPI | 8 |  |
|  | Tissue Development | proliferation of endothelial cells | 1.74E-03 | Increased | 2.040 | F2RL1,FN1,IGF1,NPPC,PRKCA,PTEN,RUNX2,TFPI | 8 |  |
|  | Cardiovascular Development, Function | proliferation of endothelial cells | 1.74E-03 | Increased | 2.040 | F2RL1,FN1,IGF1,NPPC,PRKCA,PTEN,RUNX2,TFPI | 8 |  |
|  | Cellular Function and Maintenance | exocytosis | 2.10E-03 | Increased | 2.195 | F2RL1,IGF1,PRKCA,RAB27A,SGK1,SYTL2 | 6 |  |
|  | Molecular Transport | exocytosis | 2.10E-03 | Increased | 2.195 | F2RL1,IGF1,PRKCA,RAB27A,SGK1,SYTL2 | 6 |  |
|  | Organismal Development | vasculogenesis | 2.25E-03 | Increased | 2.326 | ANXA2,ATF3,F2RL1,FN1,IGF1,ITGAV,NPPC,ODC1,PRKCA,PTEN,ROR1,RUNX2,TFPI,TNFAIP3 | 14 |  |
|  | Cardiovascular Development, Function | vasculogenesis | 2.25E-03 | Increased | 2.326 | ANXA2,ATF3,F2RL1,FN1,IGF1,ITGAV,NPPC,ODC1,PRKCA,PTEN,ROR1,RUNX2,TFPI,TNFAIP3 | 14 |  |
|  | Small Molecule Biochemistry | synthesis of terpenoid | 4.48E-03 | Increased | 2.373 | ACAT2,ALDH1A3,AR,IGF1,NPC1,ODC1,RUNX2 | 7 |  |
|  | Lipid Metabolism | synthesis of terpenoid | 4.48E-03 | Increased | 2.373 | ACAT2,ALDH1A3,AR,IGF1,NPC1,ODC1,RUNX2 | 7 |  |
|  | Vitamin and Mineral Metabolism | synthesis of terpenoid | 4.48E-03 | Increased | 2.373 | ACAT2,ALDH1A3,AR,IGF1,NPC1,ODC1,RUNX2 | 7 |  |
|  | Cellular Development | proliferation of tumor cells | 4.51E-03 | Increased | 2.739 | ANXA2,ATF3,IGF1,IL1RN,ODC1,PRKCA,PTEN,TPD52,TWIST1 | 9 |  |
|  | Cellular Growth and Proliferation | proliferation of tumor cells | 4.51E-03 | Increased | 2.739 | ANXA2,ATF3,IGF1,IL1RN,ODC1,PRKCA,PTEN,TPD52,TWIST1 | 9 |  |
|  | Tumor Morphology | proliferation of tumor cells | 4.51E-03 | Increased | 2.739 | ANXA2,ATF3,IGF1,IL1RN,ODC1,PRKCA,PTEN,TPD52,TWIST1 | 9 |  |
|  |  |  |  |  |  |  |  |  |
| **PARK7** | Cellular Growth and Proliferation | proliferation of tumor cells | 3.06E-03 | Increased | 2.143 | ANXA2,ATF3,ID2,IGF1,IL1RN | 5 |  |
|  | Cellular Development | proliferation of tumor cells | 3.06E-03 | Increased | 2.143 | ANXA2,ATF3,ID2,IGF1,IL1RN | 5 |  |
|  | Tumor Morphology | proliferation of tumor cells | 3.06E-03 | Increased | 2.143 | ANXA2,ATF3,ID2,IGF1,IL1RN | 5 |  |
|  |  |  |  |  |  |  |  |  |
| **FHL2** | Cellular Development | differentiation of connective tissue cells | 7.67E-05 | Increased | 2.219 | CCL5,CTBP1,ERO1L,IGF1,INPP4B,MMP13,NPPC,SELENBP1 | 8 |  |
|  |  |  |  |  |  |  |  |  |
| **KDM1A** | Cardiovascular Development, Function | vasculogenesis | 2.38E-04 | Increased | 2.440 | ANXA2,CXCR4,F2RL1,KLF5,KLK3,MMP13,ODC1,ORM1/ORM2,PRKCA,PTPRM,RUNX2,TFPI | 12 |  |
|  | Organismal Development | vasculogenesis | 2.38E-04 | Increased | 2.440 | ANXA2,CXCR4,F2RL1,KLF5,KLK3,MMP13,ODC1,ORM1/ORM2,PRKCA,PTPRM,RUNX2,TFPI | 12 |  |
|  | Cellular Development | differentiation of cells | 1.72E-04 | Increased | 2.307 | ANXA2,CXCR4,DPYSL2,ELF3,HES1 (includes EG:15205),KLF4,KLF5,KLK3,KRT8,MAF,MMP13,NBL1 (includes EG:17965),NDRG1,ODC1,PDE4A,PRKCA,RAB27A,RELN, RLN1/RLN2,RUNX2,SOCS2,SSBP2,TRPS1 | 23 |  |
|  | Cell-To-Cell Signaling and Interaction | activation of cells | 3.11E-05 | Increased | 2.298 | ANXA2,CXCR4,F2RL1,KLF4,KRT18,KRT8,LAT2,MAF,MMP13,NDRG1,NPC1,PPAP2A,PRKCA,RAB27A,RLN1/RLN2,SOCS2 | 16 |  |
|  | Cardiovascular Development, Function | vascularization | 2.96E-03 | Increased | 2.236 | ANXA2,CXCR4,KLF5,MMP13,ODC1 | 5 |  |
|  | Developmental Disorder | hypertrophy | 8.27E-03 | Increased | 2.200 | ACTG2,HES1 (includes EG:15205),KLF5,KLK3,ODC1,PRKCA,RUNX2 | 7 |  |
|  | Hematological System Development, Function | activation of blood cells | 1.53E-03 | Increased | 2.135 | ANXA2,F2RL1,KLF4,LAT2,MAF,NDRG1,NPC1,PPAP2A,PRKCA,RAB27A,SOCS2 | 11 |  |
|  | Cell-To-Cell Signaling and Interaction | activation of blood cells | 1.53E-03 | Increased | 2.135 | ANXA2,F2RL1,KLF4,LAT2,MAF,NDRG1,NPC1,PPAP2A,PRKCA,RAB27A,SOCS2 | 11 |  |
|  | Cardiovascular Development, Function | angiogenesis | 1.97E-03 | Increased | 2.129 | ANXA2,CXCR4,F2RL1,KLF5,MMP13,ORM1/ORM2,PRKCA,PTPRM,RUNX2,TFPI | 10 |  |
|  | Cell Death | cell death of connective tissue cells | 3.86E-03 | Decreased | -2.371 | KRT18,KRT8,MICAL1,NPC1,PDE4A,PEG3,RUNX2,TNFAIP8,TRPS1 | 9 |  |
|  |  |  |  |  |  |  |  |  |
| **WDR77** | Cell Death | necrosis | 2.99E-08 | Decreased | -2.244 | ABCC1,ABCG1,AGR2,AKAP12,ANXA2,ATF3,BARD1,BCHE,CDC6 (includes EG:23834),CREB3L2,CTBP1,CXADR,DDB2,DLX1,F2RL1,GSR,ID2,IGF1,IL1RN,KLF4,KLF5,KLK3,LIG1,MALT1,MDK,MERTK,MICAL1,MYB,MYC,MYLK,NFKBIA,NR4A1,PDE4A,RCAN1,S100A11,SMAD7, STK17B,TNFAIP8,TRPS1,TWIST1 | 40 |  |
|  | Cell Death | cell death of tumor cell lines | 4.78E-07 | Decreased | -2.259 | ABCC1,AGR2,AKAP12,ANXA2,ATF3,BARD1,BCHE,CDC6 (includes EG:23834),CREB3L2,CTBP1,IGF1,IL1RN,KLF4,KLF5,KLK3,LIG1, MDK,MYB,MYC,NFKBIA,NR4A1,S100A11,SMAD7,TNFAIP8,TRPS1,TWIST1 | 26 |  |
|  | Cell Death | apoptosis of tumor cell lines | 1.57E-05 | Decreased | -2.434 | ABCC1,AKAP12,ANXA2,ATF3,BARD1,CDC6 (includes EG:23834), CTBP1,IGF1,KLF4,KLF5,KLK3,LIG1,MYB,MYC,NFKBIA,NR4A1, S100A11,SMAD7,TNFAIP8,TRPS1,TWIST1 | 21 |  |
|  | Cell Death | cell death of vascular endothelial cells | 2.34E-03 | Decreased | -2.000 | ATF3,F2RL1,IGF1,KLF5 | 4 |  |
|  | Skeletal and Muscular Development, Function | size of bone | 5.30E-03 | Increased | 2.164 | CREB3L2,IGF1,IL1RN,LIFR,MYB | 5 |  |
|  | Organ Morphology | size of bone | 5.30E-03 | Increased | 2.164 | CREB3L2,IGF1,IL1RN,LIFR,MYB | 5 |  |
|  | DNA Replication, Recombination, Repair | metabolism of DNA | 5.97E-03 | Decreased | -2.285 | BARD1,CDC6 (includes EG:23834),IGF1,LIG1,MCM7,MYC,NPPC,S100A11 | 8 |  |
|  |  |  |  |  |  |  |  |  |
| **EP300** | Cancer | cancer | 1.27E-13 | Increased | 2.323 | ABCC1,ABCC4 (includes EG:10257),ACTG2,ADARB1,AKAP12,ALDH1A3,ANTXR1,ANXA2,AR,ASRGL1,ATF3,ATP1B1,AZGP1,B4GALT1,BARD1,BMPR1A,BTG2,C16orf45,CALD1,CCL5,CDC6 (includes EG:23834),CDH3,CLGN, COLEC12,CREBBP,CTBP1,CUX1,CXCR4,CXCR7,CYP3A7,CYR61,DBI,DDB2,DDC,DHCR24,DNAJB9,DPYSL2,EAF2,EFCAB11,EPS8,ERBB2,F2RL1,FASN,FMOD,GALK2,GALNT3,GATA2,GLIPR1,GREB1,HES1 (includes EG:15205),HIBADH,HMGCR,HPGD,ID2,IDI1,IGF1,IL1RN, INSIG1,IQGAP2,ITGAV,KLF5,KLK2,KLK3,KRT18,KRT19 (human),KRT8,LGALS8,LIFR,LIG1,MAP2K4,MCM7,MDK,MXI1,MYB,MYC,MYLK,NDRG1,NFKBIA,NPAT,NPC1,OPRK1,ORM1/ORM2,PCSK6,PEG3,PGC,PHLDA2,PIK3R1,PPAP2A,PPP2CB,PRKCA,PRKD1,PURA,RAB4A,RELN,RNASE4,ROR1,RPS6KA3,RUNX2,SELENBP1,SGK1,SLC26A2,SMARCD3,SOCS2,SOX4,SSBP2,STK17B,TARP,TFPI,THRA (includes EG:21833),TNFAIP3,TPD52,TRPS1,TWIST1, WWTR1,XPA | 115 |  |
|  | Cancer | neoplasia | 3.16E-13 | Increased | 2.625 | ABCC1,ABCC4 (includes EG:10257),ACTG2,ADARB1,AKAP12,ALDH1A3,ANTXR1,ANXA2,AR,ASRGL1,ATF3,ATP1B1,AZGP1,B4GALT1,BARD1,BMPR1A,BTG2,C16orf45,CALD1,CCL5,CDC6 (includes EG:23834),CDH3,CLGN,COLEC12,CREBBP,CTBP1,CUX1,CXCR4,CXCR7,CYP3A7,CYR61,DBI,DDB2,DDC,DHCR24,DNAJB9,DPYSL2,EAF2,EFCAB11,EPS8,ERBB2,F2RL1,FASN,FMOD,FSTL1,GALK2,GALNT3,GATA2,GLIPR1,GREB1,HES1 (includes EG:15205),HIBADH,HMGCR,HPGD,ID2,IDI1,IGF1,IL1RN,INSIG1,IQGAP2,ITGAV,KLF5,KLK2,KLK3,KRT18,KRT19 (human),KRT8,LGALS8,LIFR,LIG1,MAP2K4,MCM7,MDK,MXI1,MYB,MYC,MYLK,NDRG1,NFKBIA,NPAT,NPC1,OPRK1,ORM1/ORM2,PCSK6,PEG3,PGC,PHLDA2,PIK3R1,PPAP2A,PPP2CB,PRKCA,PRKD1,PURA,RAB4A,RELN,RNASE4,ROR1,RPS6KA3,RUNX2,SELENBP1,SGK1,SLC26A2,SMARCD3,SOCS2,SOX4,SSBP2,STK17B,TARP,TFPI,THRA (includes EG:21833),TNFAIP3,TPD52,TRPS1,TWIST1,WWTR1,XPA | 116 |  |
|  | Cell Death | cell survival | 2.67E-10 | Increased | 2.060 | ABCC1,AMIGO2,ANTXR1,APP,AR,CACNB3,CCL5,CDK8,CTH,CXCR4,CXCR7,CYR61,ELF3,ERBB2,F2RL1,GALK2,GALNT3,GATA2,GSR,HES1 (includes EG:15205),ID2,IGF1,ITGAV,KLF5,KRT19 (human),LIG1,MALT1,MAP2K4,MDK,MYB,MYC,NAIP,NDRG1,NEFL,NFKBIA,PCSK6,PEG3,PIK3R1,PPAP2A,PRKCA,PTPRM,RCAN1,ROR1,SERPINI1,SGK1,SOCS2,TNFAIP3,TNFAIP8,TWIST1,VCL,XPA | 51 |  |
|  | Cellular Movement | migration of cells | 1.38E-08 | Increased | 2.326 | ABCC1,ANXA2,APP,AR,ATF3,B4GALT1,BMPR1A,BTG2,CCL5,CDH3,CTBP1,CXADR,CXCR4,CXCR7,CYR61,DPYSL2,ERBB2,F2RL1,FMOD,GATA2,HMGCR,HOXC9,ID2,ID3 (includes EG:15903),IGF1,IL1RN,ITGAV,KLF5,KLK3,KRT8,LAMC1,MAP2K4,MCM7,MDK,MYC,MYLK,NBL1 (includes EG:17965),NDRG1,NFIA,NFKBIA,NPC1,NPPC,ORM1/ORM2,PIK3R1,PPAP2A,PPIC,PRKCA,PRKD1,PTPRM,RCAN1,RELN,RLN1/RLN2,RUNX2,TFPI,TNFAIP3,TNFAIP8,TWIST1,VCL,WWTR1 | 59 |  |
|  | Cell Death | apoptosis of tumor cell lines | 1.85E-07 | Decreased | -2.015 | ABCC1,AKAP12,ANXA2,APP,AR,ATF3,BARD1,BTG2,CDC6 (includes EG:23834),CDK8,CTBP1,CTH,CYR61,DHCR24,ERBB2,FASN,GLIPR1,HES1 (includes EG:15205),ID3 (includes EG:15903),IGF1,ITGAV,KLF5,KLK3,KRT18,LGALS8,LIG1,MAP2K4,MTMR9,MYB,MYC,NAIP,NDRG1,NFKBIA,PRKCA,PRKD1,SGK1,SOX4,TNFAIP3,TNFAIP8,TPD52,TRPS1,TWIST1 | 42 |  |
|  | Cellular Movement | migration of tumor cell lines | 3.18E-07 | Increased | 2.777 | ANXA2,APP,AR,CCL5,CTBP1,CXCR4,CXCR7,CYR61,DPYSL2,ERBB2,F2RL1,IGF1,ITGAV,KRT8,MDK,NDRG1,PPIC,PRKCA,PRKD1,PTPRM,RLN1/RLN2,RUNX2,TNFAIP8,TWIST1,WWTR1 | 25 |  |
|  | Cellular Movement | cell movement of breast cell lines | 3.90E-07 | Increased | 2.056 | APP,BTG2,ELF3,ERBB2,IGF1,PRKCA,RELN,WWTR1 | 8 |  |
|  | Reproductive Development, Function | cell movement of breast cell lines | 3.90E-07 | Increased | 2.056 | APP,BTG2,ELF3,ERBB2,IGF1,PRKCA,RELN,WWTR1 | 8 |  |
|  | Cancer | cancer of cells | 3.66E-06 | Increased | 2.611 | ATF3,BMPR1A,CXCR4,ERBB2,IGF1,ITGAV,MYC,NFKBIA,RELN,RUNX2,TWIST1 | 11 |  |
|  | Cell Death | apoptosis of prostate cancer cell lines | 1.69E-05 | Decreased | -2.517 | AKAP12,AR,FASN,GLIPR1,IGF1,KLK3,MYC,PRKCA,TPD52,TRPS1, TWIST1 | 11 |  |
|  | Cancer | cancer of tumor cell lines | 2.20E-05 | Increased | 2.433 | ATF3,BMPR1A,CXCR4,ERBB2,IGF1,MYC,NFKBIA,TWIST1 | 8 |  |
|  | Cancer | metastasis of cells | 2.21E-05 | Increased | 2.611 | ATF3,BMPR1A,CXCR4,ERBB2,IGF1,ITGAV,NFKBIA,RELN,RUNX2,TWIST1 | 10 |  |
|  | Cellular Movement | migration of breast cancer cell lines | 2.70E-05 | Increased | 2.147 | CCL5,CXCR4,ERBB2,IGF1,ITGAV,NDRG1,PPIC,PRKD1,RLN1/RLN2,TNFAIP8,WWTR1 | 11 |  |
|  | Cellular Growth and Proliferation | proliferation of epithelial cells | 6.82E-05 | Increased | 2.512 | AR,B4GALT1,BMPR1A,CCNG2,ERBB2,GATA2,ID2,IGF1,KLF5,LAMC1,MAP2K4,MYB,MYC,NFKBIA,PRKCA,THRA (includes EG:21833),TWIST1 | 17 |  |
|  | Cell Morphology | reorganization of cytoskeleton | 8.70E-05 | Increased | 2.621 | AKAP12,ANTXR1,APP,AR,CXADR,EPS8,F2RL1,IGF1,ITGAV,PRKCA | 10 |  |
|  | Cellular Assembly and Organization | reorganization of cytoskeleton | 8.70E-05 | Increased | 2.621 | AKAP12,ANTXR1,APP,AR,CXADR,EPS8,F2RL1,IGF1,ITGAV,PRKCA | 10 |  |
|  | Cellular Function and Maintenance | reorganization of cytoskeleton | 8.70E-05 | Increased | 2.621 | AKAP12,ANTXR1,APP,AR,CXADR,EPS8,F2RL1,IGF1,ITGAV,PRKCA | 10 |  |
|  | Cancer | metastasis of tumor cell lines | 1.45E-04 | Increased | 2.433 | ATF3,BMPR1A,CXCR4,ERBB2,IGF1,NFKBIA,TWIST1 | 7 |  |
|  | Cell Death | cell death of liver cells | 4.36E-04 | Decreased | -2.209 | IGF1,IL1RN,IQGAP2,ITGAV,KRT8,MAP2K4,MYC,NPC1,PIK3R1,SEPP1 | 10 |  |
|  | Immunological Disease | hypersensitive reaction | 8.34E-04 | Increased | 2.412 | ATP1B1,B4GALT1,CCL5,CXCR4,DPYSL2,F2RL1,FSTL1,IDI1,IL1RN,KRT6C,MALT1,MXI1,NFKBIA,OPRK1,PGM3,TNFAIP3,VCL | 17 |  |
|  | Cell-To-Cell Signaling and Interaction | binding of tumor cell lines | 9.46E-04 | Increased | 2.131 | ANXA2,APP,AR,CXADR,CXCR4,IGF1,ITGAV,PIK3R1,PTPRM | 9 |  |
|  | Cell Death | apoptosis of liver cells | 1.35E-03 | Decreased | -2.608 | IGF1,IQGAP2,ITGAV,KRT8,MAP2K4,MYC,NPC1,SEPP1 | 8 |  |
|  | Skeletal and Muscular Development, Function | size of bone | 1.40E-03 | Increased | 2.190 | AR,GALNT3,HIVEP3,IGF1,IL1RN,LIFR,MYB,RPS6KA3,RUNX2 | 9 |  |
|  | Organ Morphology | size of bone | 1.40E-03 | Increased | 2.190 | AR,GALNT3,HIVEP3,IGF1,IL1RN,LIFR,MYB,RPS6KA3,RUNX2 | 9 |  |
|  | Cellular Development | differentiation of lymphocytes | 1.90E-03 | Decreased | -2.240 | CUX1,CXCR4,ELF3,GATA2,ID2,ID3 (includes EG:15903),IGF1,LGALS8,MALT1,MYB,MYC,NFKBIA,PIK3R1,PRKD1,RUNX2,SOX4,TNFAIP3 | 17 |  |
|  | Hematological System Development, Function | differentiation of lymphocytes | 1.90E-03 | Decreased | -2.240 | CUX1,CXCR4,ELF3,GATA2,ID2,ID3 (includes EG:15903),IGF1,LGALS8,MALT1,MYB,MYC,NFKBIA,PIK3R1,PRKD1,RUNX2,SOX4,TNFAIP3 | 17 |  |
|  | Hematopoiesis | differentiation of lymphocytes | 1.90E-03 | Decreased | -2.240 | CUX1,CXCR4,ELF3,GATA2,ID2,ID3 (includes EG:15903),IGF1,LGALS8,MALT1,MYB,MYC,NFKBIA,PIK3R1,PRKD1,RUNX2,SOX4,TNFAIP3 | 17 |  |
|  |  |  |  |  |  |  |  |  |
| **PKN1** | Cellular Growth and Proliferation | proliferation of cells | 1.97E-02 | Decreased | -2.252 | ATF3,CXADR,ELF3,KLF4,KLK3,NPPC,TNFAIP3 | 7 |  |
|  |  |  |  |  |  |  |  |  |
| **NCOA1** | Cellular Growth and Proliferation | colony formation of tumor cell lines | 7.26E-03 | Decreased | -2.000 | ATF3,HES1 (includes EG:15205),HPGD,MYC | 4 |  |
|  |  |  |  |  |  |  |  |  |
| **NCOA2** | Cell Death | cell death of tumor cell lines | 4.71E-06 | Decreased | -2.092 | ABCC1,ABCC4 (includes EG:10257),AKAP12,APP,BARD1,BMPR1B,CDC6 (includes EG:23834),FASN,IL1RN,KLK3,LIG1,MTMR9,NDRG1,PRKCA,SERPINI1 | 15 |  |
|  | Cell Death | necrosis | 5.47E-05 | Decreased | -2.738 | ABCC1,ABCC4 (includes EG:10257),AKAP12,APP,BARD1,BMPR1B,CDC6 (includes EG:23834),CXADR,CXCR4,FASN,IL1RN,KLK3,LIG1,MERTK,MTMR9,NDRG1,PRKCA,SEPP1,SERPINI1 | 19 |  |
|  | Cell Death | apoptosis of tumor cell lines | 9.53E-05 | Decreased | -2.048 | ABCC1,AKAP12,APP,BARD1,BMPR1B,CDC6 (includes EG:23834),FASN,KLK3,LIG1,MTMR9,NDRG1,PRKCA | 12 |  |
|  | Cellular Development | differentiation of cells | 1.19E-04 | Increased | 2.551 | ABCC4 (includes EG:10257),APP,BMPR1B,CXCR4,HES6,IL1RN,INSIG1,KLK3,LIFR,MAF,MERTK,NBL1 (includes EG:17965),NDRG1,PRKCA,RLN1/RLN2,SOCS2 | 16 |  |
|  | Inflammatory Response | inflammatory response | 5.41E-03 | Increased | 2.357 | ABCC1,APP,CXADR,CXCR4,IL1RN,MAF,PRKCA | 7 |  |
|  | Cellular Function and Maintenance | cellular homeostasis | 7.30E-03 | Increased | 2.540 | ABCC1,APP,BARD1,CXCR4,IL1RN,LAT2,MAF,MERTK,NDRG1,PRKCA | 10 |  |
|  |  |  |  |  |  |  |  |  |
| **NCOA3** | Cellular Development | differentiation of cells | 1.01E-03 | Increased | 2.445 | BMPR1B,KLF4,MMP13,NBL1 (includes EG:17965),NDRG1,RAB27A,RELN,SOCS2,SOX4,TXNIP | 10 |  |
|  | Cellular Growth and Proliferation | colony formation of cells | 6.14E-03 | Decreased | -2.000 | AKAP12,KLF4,NDRG1,SOCS2 | 4 |  |
|  |  |  |  |  |  |  |  |  |
| **HTATIP2** | Cellular Movement | cell movement | 4.48E-07 | Increased | 2.022 | ABCC1,ABHD2,ADAM9,APP,B4GALT1,BMPR1A,CCL5,CTBP1,CXCR4,ELF3,EPS8,F2RL1,ID2,IL1RN,KLK3,KRT19 (human),LIFR,MMP13,MYLK,NBL1 (includes EG:17965),NFIX,ORM1/ORM2,PAK2,PPIC,PRKCA,RELN,RPS6KA3,SOCS2,SORD | 29 |  |
|  | Cell-To-Cell Signaling, Interaction | activation of cells | 6.15E-05 | Increased | 2.638 | ADAM9,APP,B4GALT1,CCL5,CXCR4,F2RL1,IL1RN,KRT18,LAT2,LIFR,MAF,MERTK,MMP13,PRKCA,RAB27A,SOCS2 | 16 |  |
|  | Cell-To-Cell Signaling, Interaction | activation of myeloid cells | 3.15E-04 | Increased | 2.367 | bias | ADAM9,APP,CCL5,F2RL1,IL1RN,MAF,RAB27A | 7 |
|  | Hematological System Development, Function | activation of myeloid cells | 3.15E-04 | Increased | 2.367 | bias | ADAM9,APP,CCL5,F2RL1,IL1RN,MAF,RAB27A | 7 |
|  | Immune Cell Trafficking | activation of myeloid cells | 3.15E-04 | Increased | 2.367 | bias | ADAM9,APP,CCL5,F2RL1,IL1RN,MAF,RAB27A | 7 |
|  | Inflammatory Response | activation of myeloid cells | 3.15E-04 | Increased | 2.367 | bias | ADAM9,APP,CCL5,F2RL1,IL1RN,MAF,RAB27A | 7 |
|  | Cellular Movement | cell movement of phagocytes | 5.55E-04 | Increased | 2.025 | ABCC1,APP,B4GALT1,CCL5,CXCR4,F2RL1,IL1RN,MYLK,NBL1 (includes EG:17965),PRKCA | 10 |  |
|  | Hematological System Development, Function | cell movement of phagocytes | 5.55E-04 | Increased | 2.025 | ABCC1,APP,B4GALT1,CCL5,CXCR4,F2RL1,IL1RN,MYLK,NBL1 (includes EG:17965),PRKCA | 10 |  |
|  | Immune Cell Trafficking | cell movement of phagocytes | 5.55E-04 | Increased | 2.025 | ABCC1,APP,B4GALT1,CCL5,CXCR4,F2RL1,IL1RN,MYLK,NBL1 (includes EG:17965),PRKCA | 10 |  |
|  | Inflammatory Response | cell movement of phagocytes | 5.55E-04 | Increased | 2.025 | ABCC1,APP,B4GALT1,CCL5,CXCR4,F2RL1,IL1RN,MYLK,NBL1 (includes EG:17965),PRKCA | 10 |  |
|  | Molecular Transport | release of lipid | 5.94E-04 | Increased | 2.382 | bias | ABCC1,APP,CCL5,F2RL1,IL1RN,PRKCA | 6 |
|  | Lipid Metabolism | release of lipid | 5.94E-04 | Increased | 2.382 | bias | ABCC1,APP,CCL5,F2RL1,IL1RN,PRKCA | 6 |
|  | Small Molecule Biochemistry | release of lipid | 5.94E-04 | Increased | 2.382 | bias | ABCC1,APP,CCL5,F2RL1,IL1RN,PRKCA | 6 |
|  | Molecular Transport | release of eicosanoid | 6.03E-04 | Increased | 2.183 | bias | ABCC1,CCL5,F2RL1,IL1RN,PRKCA | 5 |
|  | Lipid Metabolism | release of eicosanoid | 6.03E-04 | Increased | 2.183 | bias | ABCC1,CCL5,F2RL1,IL1RN,PRKCA | 5 |
|  | Small Molecule Biochemistry | release of eicosanoid | 6.03E-04 | Increased | 2.183 | bias | ABCC1,CCL5,F2RL1,IL1RN,PRKCA | 5 |
|  | Inflammatory Response | inflammatory response | 6.43E-04 | Increased | 2.737 | ABCC1,APP,B4GALT1,CCL5,CXCR4,F2RL1,IL1RN,MAF,MYLK,ORM1/ORM2,PRKCA,RAB27A | 12 |  |
|  | Organismal Survival | organismal death | 7.27E-04 | Decreased | -2.617 | bias | ABCC1,ABCC4 (includes EG:10257),ADAM9,ANKH,APP,ATP1B1,B4GALT1,BMPR1A,CITED1,CREB3L2,CXCR4,EAF2,F2RL1,IL1RN,LIFR,MAF,MERTK,NFIX,PRKCA,SSBP2 | 20 |
|  | Cell-To-Cell Signaling and Interaction | activation of leukocytes | 9.14E-04 | Increased | 2.147 | ADAM9,APP,CCL5,F2RL1,IL1RN,LAT2,MAF,MERTK,PRKCA,RAB27A,SOCS2 | 11 |  |
|  | Hematological System Development, Function | activation of leukocytes | 9.14E-04 | Increased | 2.147 | ADAM9,APP,CCL5,F2RL1,IL1RN,LAT2,MAF,MERTK,PRKCA,RAB27A,SOCS2 | 11 |  |
|  | Immune Cell Trafficking | activation of leukocytes | 9.14E-04 | Increased | 2.147 | ADAM9,APP,CCL5,F2RL1,IL1RN,LAT2,MAF,MERTK,PRKCA,RAB27A,SOCS2 | 11 |  |
|  | Inflammatory Response | activation of leukocytes | 9.14E-04 | Increased | 2.147 | ADAM9,APP,CCL5,F2RL1,IL1RN,LAT2,MAF,MERTK,PRKCA,RAB27A,SOCS2 | 11 |  |
|  | Cellular Movement | infiltration of granulocytes | 2.32E-03 | Increased | 2.159 | B4GALT1,CCL5,F2RL1,IL1RN,PRKCA | 5 |  |
|  | Hematological System Development, Function | infiltration of granulocytes | 2.32E-03 | Increased | 2.159 | B4GALT1,CCL5,F2RL1,IL1RN,PRKCA | 5 |  |
|  | Immune Cell Trafficking | infiltration of granulocytes | 2.32E-03 | Increased | 2.159 | B4GALT1,CCL5,F2RL1,IL1RN,PRKCA | 5 |  |
|  | Cellular Movement | T cell migration | 5.55E-03 | Increased | 2.186 | bias | ABCC1,APP,CCL5,CXCR4,F2RL1 | 5 |
|  | Hematological System Development, Function | T cell migration | 5.55E-03 | Increased | 2.186 | bias | ABCC1,APP,CCL5,CXCR4,F2RL1 | 5 |
|  | Immune Cell Trafficking | T cell migration | 5.55E-03 | Increased | 2.186 | bias | ABCC1,APP,CCL5,CXCR4,F2RL1 | 5 |
|  | Cell-mediated Immune Response | T cell migration | 5.55E-03 | Increased | 2.186 | bias | ABCC1,APP,CCL5,CXCR4,F2RL1 | 5 |
|  |  |  |  |  |  |  |  |  |
| **KAT5** | Cancer | cancer | 4.14E-09 | Increased | 2.182 | ABCC4 (includes EG:10257),AGR2,AKAP12,ALDH1A3,ANTXR1,ATF3,ATP1B1,BMPR1A,CCL5,CDH3,CIRBP,CRAT,CTBP1,CXCR4,CXCR7,CYP3A7,DDB2,DNAJB9,DPYSL2,EFCAB11,EPS8,GALNT3,GREB1,GUCY1A3,HES1 (includes EG:15205),HOXB13,HPGD,ID2,IDH1,IL1RN,KLF4,KLF5,KLK2,KLK3,KRT19 (human),MAF,MATN2,MMP13,MYB,MYLK,NAT1 (includes EG:116632),NDRG1,NPAT,ODC1,OPRK1,OSR2,PAK1IP1,PGC,PLCB4,PPAP2A,PRKCA,PTPN21,RELN,RNASE4,RPS6KA3,RUNX2,SELENBP1,SGK1,SOCS2,SOX4,SPDEF,STK17B,TARP,TMPRSS2,TNFAIP3,TRPS1,TWIST1 | 67 |  |
|  | Cellular Development | differentiation of cells | 1.20E-07 | Increased | 2.065 | ABCC4 (includes EG:10257),APP,ATF3,BMPR1A,CAMKK2,CBX1,CCL5,CTBP1,CXCR4,DPYSL2,HES1 (includes EG:15205),HES6,HOXB13,ID2,IL1RN,INPP4B,KLF4,KLF5,KLK3,LAMC1,MAF,MALT1,MMP13,MYB,NBL1 (includes EG:17965),NDRG1,NPPC,ODC1,PDE4A,PRKCA,RELN,RLN1/RLN2,RPS6KA3,RUNX2,SELENBP1,SNRK,SOCS2,SOX4,TNFAIP3,TRPS1,TWIST1 | 41 |  |
|  | Organismal Injury and Abnormalities | cytosis | 1.60E-03 | Increased | 2.210 | ADD3,APP,CXCR4,IDH1,IL1RN,MYB,PDE4A,PRKCA | 8 |  |

| **Supplementary File 3C. Ingenuity Pathway Analysis canonical pathways associated with coregulator-dependent AR target gene sets** | | | | |
| --- | --- | --- | --- | --- |
| **Gene set** | **Ingenuity Canonical Pathways** | **-log (p-value)** | **Ratio** | **Molecules** |
|  |  |  |  |  |
| **SMARCC1** | Role of Tissue Factor in Cancer | 2.28 | 0.0351 | ITGAV,RPS6KA3,CYR61,PRKCA |
|  | NRF2-mediated Oxidative Stress Response | 2.24 | 0.0262 | GSTT2/GSTT2B,MAF,DNAJC3,ACTG2,PRKCA |
|  | ERK5 Signaling | 2.14 | 0.0469 | MYC,SGK1,RPS6KA3 |
|  | Xenobiotic Metabolism Signaling | 2.03 | 0.0203 | GSTT2/GSTT2B,CYP3A7,MAF,UGT2B15,FMO5,PRKCA |
|  | Growth Hormone Signaling | 2.03 | 0.04 | SOCS2,RPS6KA3,PRKCA |
|  | PI3K Signaling in B Lymphocytes | 2.01 | 0.028 | PLCB4,ATF3,NFKBIA,MALT1 |
|  | Cellular Effects of Sildenafil (Viagra) | 1.96 | 0.0258 | MYLK,PLCB4,GUCY1A3,ACTG2 |
|  | NF-κB Activation by Viruses | 1.94 | 0.0366 | NFKBIA,ITGAV,PRKCA |
|  | Prolactin Signaling | 1.94 | 0.0375 | MYC,SOCS2,PRKCA |
|  | Caveolar-mediated Endocytosis Signaling | 1.93 | 0.0353 | ITGAV,ACTG2,PRKCA |
|  |  |  |  |  |
| **BAG1** | Regulation of Actin-based Motility by Rho | 3.63 | 0.033 | MYLK,RHOU,ACTG2 |
|  | Integrin Signaling | 2.55 | 0.0143 | MYLK,RHOU,ACTG2 |
|  | Signaling by Rho Family GTPases | 2.37 | 0.0119 | MYLK,RHOU,ACTG2 |
|  | Virus Entry via Endocytic Pathways | 2.13 | 0.02 | ACTG2,CXADR |
|  | RhoA Signaling | 1.95 | 0.0175 | MYLK,ACTG2 |
|  | Metabolism of Xenobiotics by Cytochrome P450 | 1.93 | 0.0102 | GSTT2/GSTT2B,UGT2B15 |
|  | Atherosclerosis Signaling | 1.9 | 0.0153 | ORM1/ORM2,CXCR4 |
|  | Cellular Effects of Sildenafil (Viagra) | 1.79 | 0.0129 | MYLK,ACTG2 |
|  | CXCR4 Signaling | 1.7 | 0.0119 | CXCR4,RHOU |
|  | Tight Junction Signaling | 1.68 | 0.0123 | MYLK,ACTG2 |
|  |  |  |  |  |
| **SMARCA4** | NRF2-mediated Oxidative Stress Response | 4.06 | 0.0419 | PIK3R1,MAF,DNAJC3,ACTG2,DNAJB9,FKBP5,ABCC4 (includes EG:10257), PRKCA |
|  | Aldosterone Signaling in Epithelial Cells | 3.63 | 0.0405 | PLCB4,SGK1,PIK3R1,DNAJC3,DNAJB9,HSPA2,PRKCA |
|  | PI3K Signaling in B Lymphocytes | 3.22 | 0.042 | PLCB4,ATF3,NFKBIA,PIK3R1,MALT1,PTEN |
|  | Virus Entry via Endocytic Pathways | 3.14 | 0.05 | B2M,PIK3R1,ACTG2,CXADR,PRKCA |
|  | Glucocorticoid Receptor Signaling | 2.96 | 0.0272 | AR,NFKBIA,NCOA2,IL1RN,SGK1,PIK3R1,FKBP5,HSPA2 |
|  | Role of Tissue Factor in Cancer | 2.77 | 0.0439 | F2RL1,PIK3R1,ITGAV,PTEN,PRKCA |
| **Supplementary File 3C, con’t.** | | | | |
| **Gene set** | **Ingenuity Canonical Pathways** | **-log (p-value)** | **Ratio** | **Molecules** |
| **SMARCA4** | Xenobiotic Metabolism Signaling | 2.75 | 0.0271 | ALDH4A1,ALDH1A3,CYP3A7,PIK3R1,MAF,UGT2B15,FMO5,PRKCA |
|  | NF-κB Activation by Viruses | 2.58 | 0.0488 | NFKBIA,PIK3R1,ITGAV,PRKCA |
|  | Arginine and Proline Metabolism | 2.58 | 0.0226 | ALDH4A1,SMS,ALDH1A3,ODC1 |
|  | Caveolar-mediated Endocytosis Signaling | 2.56 | 0.0471 | B2M,ITGAV,ACTG2,PRKCA |
|  |  |  |  |  |
| **CTNNB1** | OX40 Signaling Pathway | 1.89E00 | 2.13E-02 | B2M,NFKBIA |
|  | Gα12/13 Signaling | 1.62E00 | 1.57E-02 | NFKBIA,CDH3 |
|  | Relaxin Signaling | 1.52E00 | 1.27E-02 | NFKBIA,PDE7A |
|  | Molecular Mechanisms of Cancer | 1.51E00 | 7.94E-03 | BMPR1B,NFKBIA,RHOU |
|  | Cardiomyocyte Differentiation via BMP Receptors | 1.44E00 | 5E-02 | BMPR1B |
|  | Lipid Antigen Presentation by CD1 | 1.4E00 | 4.35E-02 | B2M |
|  | Acute Phase Response Signaling | 1.35E00 | 1.13E-02 | FN1,NFKBIA |
|  | NF-κB Signaling | 1.34E00 | 1.14E-02 | BMPR1B,NFKBIA |
|  | RhoGDI Signaling | 1.31E00 | 1.01E-02 | CDH3,RHOU |
|  | Production of Nitric Oxide and Reactive Oxygen Species in Macrophages | 1.29E00 | 9.52E-03 | NFKBIA,RHOU |
|  |  |  |  |  |
| **CAV1** | Cell Cycle Control of Chromosomal Replication | 2.76 | 0.0645 | CDC6 (includes EG:23834),MCM7 |
|  | Aldosterone Signaling in Epithelial Cells | 2.26 | 0.0173 | HSPA8,DNAJC3,DNAJB9 |
|  | NRF2-mediated Oxidative Stress Response | 2.1 | 0.0157 | GSTT2/GSTT2B,DNAJC3,DNAJB9 |
|  | Protein Ubiquitination Pathway | 1.69 | 0.0112 | HSPA8,DNAJC3,DNAJB9 |
|  | Aryl Hydrocarbon Receptor Signaling | 1.4 | 0.0126 | GSTT2/GSTT2B,MCM7 |
|  | Endoplasmic Reticulum Stress Pathway | 1.39 | 0.0556 | DNAJC3 |
|  | Notch Signaling | 1.08 | 0.0233 | HES1 (includes EG:15205) |
|  | Actin Cytoskeleton Signaling | 1.05 | 0.0083 | IQGAP2,FN1 |
|  | LPS/IL-1 Mediated Inhibition of RXR Function | 1.05 | 0.00847 | GSTT2/GSTT2B,FMO5 |
|  | β-alanine Metabolism | 1.02 | 0.0112 | ACAD8 |
|  |  |  |  |  |
| **PARK7** | Role of Osteoblasts, Osteoclasts and Chondrocytes in Rheumatoid Arthritis | 3.7E00 | 2.09E-02 | BMPR1B,NFKBIA,IGF1,IL1RN,SMAD6 |
|  | Cardiomyocyte Differentiation via BMP Receptors | 3.04E00 | 1E-01 | BMPR1B,SMAD6 |
| **Supplementary File 3C, con’t.** | | | | |
| **Gene set** | **Ingenuity Canonical Pathways** | **-log (p-value)** | **Ratio** | **Molecules** |
| **PARK7** | Huntington's Disease Signaling | 2.7E00 | 1.69E-02 | PLCB4,IGF1,SGK1,HSPA2 |
|  | Glucocorticoid Receptor Signaling | 2.4E00 | 1.36E-02 | NFKBIA,IL1RN,SGK1,HSPA2 |
|  | PI3K Signaling in B Lymphocytes | 2.37E00 | 2.1E-02 | PLCB4,ATF3,NFKBIA |
|  | Role of Macrophages, Fibroblasts and Endothelial Cells in Rheumatoid Arthritis | 2.17E00 | 1.2E-02 | PLCB4,FN1,NFKBIA,IL1RN |
|  | Inositol Phosphate Metabolism | 2.17E00 | 1.52E-02 | PLCB4,SGK1,MAK |
|  | Aldosterone Signaling in Epithelial Cells | 2.16E00 | 1.73E-02 | PLCB4,SGK1,HSPA2 |
|  | Acute Phase Response Signaling | 2.09E00 | 1.69E-02 | FN1,NFKBIA,IL1RN |
|  | NF-κB Signaling | 2.08E00 | 1.71E-02 | BMPR1B,NFKBIA,IL1RN |
|  |  |  |  |  |
| **FHL2** | O-Glycan Biosynthesis | 2.45E00 | 4.26E-02 | ST6GALNAC1,GALNT3 |
|  | Role of IL-17F in Allergic Inflammatory Airway Diseases | 2.22E00 | 4.35E-02 | IGF1,MMP13 |
|  | Hepatic Fibrosis / Hepatic Stellate Cell Activation | 2.2E00 | 2.04E-02 | IGF1,MMP13,CCL5 |
|  | Role of IL-17A in Arthritis | 2.03E00 | 3.17E-02 | MMP13,CCL5 |
|  | Pathogenesis of Multiple Sclerosis | 1.62E00 | 1.11E-01 | CCL5 |
|  | Differential Regulation of Cytokine Production in Macrophages and T Helper Cells by IL-17A and IL-17F | 1.32E00 | 5.56E-02 | CCL5 |
|  | PI3K Signaling in B Lymphocytes | 1.31E00 | 1.4E-02 | ATF3,MALT1 |
|  | Differential Regulation of Cytokine Production in Intestinal Epithelial Cells by IL-17A and IL-17F | 1.22E00 | 4.35E-02 | CCL5 |
|  | IL-17A Signaling in Gastric Cells | 1.18E00 | 4E-02 | CCL5 |
|  | Axonal Guidance Signaling | 1.06E00 | 6.98E-03 | MICAL1,IGF1,MMP13 |
|  |  |  |  |  |
| **KDM1A** | VDR/RXR Activation | 3.25E00 | 4.94E-02 | RUNX2,HES1 (includes EG:15205),KLF4,PRKCA |
|  | Chemokine Signaling | 2.34E00 | 4.11E-02 | PLCB4,CXCR4,PRKCA |
|  | CXCR4 Signaling | 2.19E00 | 2.38E-02 | PLCB4,CXCR4,RHOU,PRKCA |
|  | N-Glycan Degradation | 2.12E00 | 7.41E-02 | MANEA,SI |
|  | O-Glycan Biosynthesis | 1.95E00 | 4.26E-02 | ST6GALNAC1,GALNT3 |
|  | Axonal Guidance Signaling | 1.93E00 | 1.4E-02 | DPYSL2,PLCB4,MICAL1,CXCR4,MMP13,PRKCA |
|  | Xenobiotic Metabolism Signaling | 1.92E00 | 1.69E-02 | ALDH4A1,CYP3A7,MAF,UGT2B15,PRKCA |
| **Supplementary File 3C, con’t.** | | | | |
| **Gene set** | **Ingenuity Canonical Pathways** | **-log (p-value)** | **Ratio** | **Molecules** |
|  | Cholecystokinin/Gastrin-mediated Signaling | 1.88E00 | 2.83E-02 | PLCB4,RHOU,PRKCA |
|  | Leukocyte Extravasation Signaling | 1.86E00 | 2.01E-02 | CXCR4,MMP13,ACTG2,PRKCA |
|  | Role of Tissue Factor in Cancer | 1.79E00 | 2.63E-02 | F2RL1,MMP13,PRKCA |
|  |  |  |  |  |
| **WDR77** | Protein Kinase A Signaling | 2.52E00 | 2.1E-02 | AKAP12,MYLK,GNB4,PLCB4,NFKBIA,PDE4A,PPP1CB |
|  | NRF2-mediated Oxidative Stress Response | 2.32E00 | 2.62E-02 | GSR,GSTM3,ABCC1,MAF,DNAJB9 |
|  | CCR3 Signaling in Eosinophils | 2.28E00 | 3.17E-02 | MYLK,GNB4,PLCB4,PPP1CB |
|  | PI3K Signaling in B Lymphocytes | 2.07E00 | 2.8E-02 | PLCB4,ATF3,NFKBIA,MALT1 |
|  | Cardiac β-adrenergic Signaling | 2.06E00 | 2.6E-02 | AKAP12,GNB4,PDE4A,PPP1CB |
|  | Cellular Effects of Sildenafil (Viagra) | 2.02E00 | 2.58E-02 | MYLK,PLCB4,PDE4A,PPP1CB |
|  | Aryl Hydrocarbon Receptor Signaling | 2E00 | 2.52E-02 | MYC,ALDH4A1,GSTM3,MCM7 |
|  | LPS/IL-1 Mediated Inhibition of RXR Function | 1.98E00 | 2.12E-02 | ALDH4A1,IL1RN,CYP3A7,GSTM3,ABCG1 |
|  | Cell Cycle Control of Chromosomal Replication | 1.93E00 | 6.45E-02 | CDC6 (includes EG:23834),MCM7 |
|  | G Protein Signaling Mediated by Tubby | 1.76E00 | 4.88E-02 | GNB4,PLCB4 |
|  |  |  |  |  |
| **EP300** | NRF2-mediated Oxidative Stress Response | 5.68 | 0.0681 | MAP2K4,PIK3R1,CREBBP,DNAJC3,DNAJB9,GSR,GSTT2/GSTT2B, ABCC1, ACTG2,GCLM,ABCC4 (includes EG:10257),PRKD1,PRKCA |
|  | Production of Nitric Oxide and Reactive Oxygen Species in Macrophages | 3.57 | 0.0476 | MAP2K4,PPP2CB,ORM1/ORM2,NFKBIA,PIK3R1,CREBBP,RHOU, PPP1CB, PRKD1,PRKCA |
|  | Growth Hormone Signaling | 3.35 | 0.08 | IGF1,PIK3R1,SOCS2,RPS6KA3,PRKD1,PRKCA |
|  | Mouse Embryonic Stem Cell Pluripotency | 3.34 | 0.0707 | LIFR,MYC,ID2,BMPR1A,PIK3R1,CREBBP,ID3 (includes EG:15903) |
|  | Prolactin Signaling | 3.19 | 0.075 | MYC,PIK3R1,CREBBP,SOCS2,PRKD1,PRKCA |
|  | TNFR2 Signaling | 3.12 | 0.118 | MAP2K4,NFKBIA,TNFAIP3,NAIP |
|  | Xenobiotic Metabolism Signaling | 3.07 | 0.0407 | MAP2K4,GSTT2/GSTT2B,ALDH4A1,PPP2CB,ALDH1A3,CYP3A7, PIK3R1, REBBP,UGT2B15,FMO5,PRKD1,PRKCA |
|  | Protein Kinase A Signaling | 2.7 | 0.0359 | AKAP12,MYLK,ADD3,NFKBIA,PDE7A,KDELR2,CREBBP,KDELR3, PDE4A, PPP1CB,PRKD1,PRKCA |
|  | Glutamate Metabolism | 2.63 | 0.0519 | GSR,ALDH4A1,GCLM,CPS1 |
|  | Nicotinate and Nicotinamide Metabolism | 2.44 | 0.0435 | MAP2K4,CDK8,SGK1,MAPK6,ENPP5,MAK |
|  |  |  |  |  |
| **PKN1** | TNFR2 Signaling | 1.64E00 | 2.94E-02 | TNFAIP3 |
|  | TNFR1 Signaling | 1.43E00 | 1.89E-02 | TNFAIP3 |
| **Supplementary File 3C, con’t.** | | | | |
| **Gene set** | **Ingenuity Canonical Pathways** | **-log (p-value)** | **Ratio** | **Molecules** |
|  | CD40 Signaling | 1.31E00 | 1.43E-02 | TNFAIP3 |
| **PKN1** | PXR/RXR Activation | 1.28E00 | 1.14E-02 | CYP3A7 |
|  | Linoleic Acid Metabolism | 1.22E00 | 9.17E-03 | CYP3A7 |
|  | VDR/RXR Activation | 1.22E00 | 1.23E-02 | KLF4 |
|  | Prostate Cancer Signaling | 1.21E00 | 1.03E-02 | KLK3 |
|  | Virus Entry via Endocytic Pathways | 1.16E00 | 1E-02 | CXADR |
|  | HGF Signaling | 1.12E00 | 9.52E-03 | ELF3 |
|  | Telomerase Signaling | 1.12E00 | 9.8E-03 | ELF3 |
|  |  |  |  |  |
| **NCOA1** | ILK Signaling | 2.53E00 | 2.08E-02 | MYC,FN1,RHOU,ACTG2 |
|  | Glioblastoma Multiforme Signaling | 1.9E00 | 1.79E-02 | MYC,PLCB4,RHOU |
|  | Inositol Phosphate Metabolism | 1.87E00 | 1.52E-02 | PLCB4,SGK1,MAPK6 |
|  | Glioma Invasiveness Signaling | 1.84E00 | 3.33E-02 | ITGAV,RHOU |
|  | Role of Macrophages, Fibroblasts and Endothelial Cells in Rheumatoid Arthritis | 1.79E00 | 1.2E-02 | MYC,PLCB4,FN1,MMP13 |
|  | Endothelin-1 Signaling | 1.78E00 | 1.62E-02 | MYC,PLCB4,MAPK6 |
|  | ERK5 Signaling | 1.77E00 | 3.12E-02 | MYC,SGK1 |
|  | Leukocyte Extravasation Signaling | 1.63E00 | 1.51E-02 | CLDN12,MMP13,ACTG2 |
|  | Caveolar-mediated Endocytosis Signaling | 1.62E00 | 2.35E-02 | ITGAV,ACTG2 |
|  | Integrin Signaling | 1.58E00 | 1.43E-02 | ITGAV,RHOU,ACTG2 |
|  |  |  |  |  |
| **NCOA2** | Chemokine Signaling | 3.04E00 | 4.11E-02 | CXCR4,PPP1CB,PRKCA |
|  | NRF2-mediated Oxidative Stress Response | 2.83E00 | 2.09E-02 | ABCC1,MAF,ABCC4 (includes EG:10257),PRKCA |
|  | Factors Promoting Cardiogenesis in Vertebrates | 2.75E00 | 3.16E-02 | BMPR1B,CDC6 (includes EG:23834),PRKCA |
|  | Cell Cycle Control of Chromosomal Replication | 2.6E00 | 6.45E-02 | CDC6 (includes EG:23834),MCM7 |
|  | Cholecystokinin/Gastrin-mediated Signaling | 2.57E00 | 2.83E-02 | IL1RN,RHOU,PRKCA |
|  | Hepatic Cholestasis | 2.18E00 | 1.71E-02 | IL1RN,ABCC1,PRKCA |
|  | CXCR4 Signaling | 2.07E00 | 1.79E-02 | CXCR4,RHOU,PRKCA |
|  | Production of Nitric Oxide and Reactive Oxygen Species in Macrophages | 1.89E00 | 1.43E-02 | RHOU,PPP1CB,PRKCA |
|  | Growth Hormone Signaling | 1.82E00 | 2.67E-02 | SOCS2,PRKCA |
| **Supplementary File 3C, con’t.** | | | | |
| **Gene set** | **Ingenuity Canonical Pathways** | **-log (p-value)** | **Ratio** | **Molecules** |
| **NCOA2** | Thrombin Signaling | 1.81E00 | 1.46E-02 | RHOU,PPP1CB,PRKCA |
| **NCOA3** | Atherosclerosis Signaling | 1.83E00 | 1.53E-02 | ORM1/ORM2,MMP13 |
|  | Fatty Acid Metabolism | 1.81E00 | 1.09E-02 | ALDH2,CYP3A7 |
|  | Tryptophan Metabolism | 1.75E00 | 8.51E-03 | ALDH2,CYP3A7 |
|  | Acute Phase Response Signaling | 1.55E00 | 1.13E-02 | ORM1/ORM2,SOCS2 |
|  | Cardiomyocyte Differentiation via BMP Receptors | 1.55E00 | 5E-02 | BMPR1B |
|  | Ascorbate and Aldarate Metabolism | 1.49E00 | 1.23E-02 | ALDH2 |
|  | Role of Osteoblasts, Osteoclasts and Chondrocytes in Rheumatoid Arthritis | 1.34E00 | 8.37E-03 | BMPR1B,MMP13 |
|  | O-Glycan Biosynthesis | 1.29E00 | 2.13E-02 | GALNT3 |
|  | IL-9 Signaling | 1.28E00 | 2.5E-02 | SOCS2 |
|  | Oncostatin M Signaling | 1.28E00 | 2.86E-02 | MMP13 |
|  |  |  |  |  |
| **STAT3** | Propanoate Metabolism | 3.49E00 | 3.31E-02 | ALDH2,ACSL3,DHCR24,ACAD8 |
|  | Fatty Acid Metabolism | 3.1E00 | 2.73E-02 | ALDH2,ACSL3,CYP1A2,CYP3A7,ACAD8 |
|  | Atherosclerosis Signaling | 2.26E00 | 3.05E-02 | ORM1/ORM2,CXCR4,IL1RN,MMP13 |
|  | Leukocyte Extravasation Signaling | 2.25E00 | 2.51E-02 | CLDN8,CXCR4,MMP13,ACTG2,PRKCA |
|  | Thrombin Signaling | 2.25E00 | 2.43E-02 | MYLK,GNB4,RHOU,GATA2,PRKCA |
|  | Starch and Sucrose Metabolism | 2.12E00 | 1.81E-02 | SI,UGT2B15,ENPP5 |
|  | Tryptophan Metabolism | 2.11E00 | 1.7E-02 | ALDH2,CYP1A2,DHCR24,CYP3A7 |
|  | Linoleic Acid Metabolism | 1.94E00 | 2.75E-02 | CYP1A2,CYP3A7,FADS1 |
|  | Signaling by Rho Family GTPases | 1.91E00 | 1.98E-02 | MYLK,GNB4,WASF3,RHOU,ACTG2 |
|  | CXCR4 Signaling | 1.88E00 | 2.38E-02 | GNB4,CXCR4,RHOU,PRKCA |
|  |  |  |  |  |
| **HTATIP2** | Cardiomyocyte Differentiation via BMP Receptors | 4E00 | 1.5E-01 | BMPR1B,BMPR1A,SMAD6 |
|  | Chemokine Signaling | 3.37E00 | 5.48E-02 | CXCR4,PPP1CB,CCL5,PRKCA |
|  | VDR/RXR Activation | 3.16E00 | 4.94E-02 | NCOA2,HES1 (includes EG:15205),CCL5,PRKCA |
|  | Regulation of Actin-based Motility by Rho | 3.04E00 | 4.4E-02 | MYLK,PAK2,PPP1CB,ACTG2 |
|  | NRF2-mediated Oxidative Stress Response | 2.65E00 | 2.62E-02 | ABCC1,MAF,ACTG2,ABCC4 (includes EG:10257),PRKCA |
|  | Role of Tissue Factor in Cancer | 2.62E00 | 3.51E-02 | F2RL1,RPS6KA3,MMP13,PRKCA |
|  | CCR3 Signaling in Eosinophils | 2.55E00 | 3.17E-02 | MYLK,PAK2,PPP1CB,PRKCA |
| **Supplementary File 3C, con’t.** | | | | |
| **Gene set** | **Ingenuity Canonical Pathways** | **-log (p-value)** | **Ratio** | **Molecules** |
| **HTATIP2** | Atherosclerosis Signaling | 2.5E00 | 3.05E-02 | ORM1/ORM2,CXCR4,IL1RN,MMP13 |
|  | Growth Hormone Signaling | 2.29E00 | 4E-02 | SOCS2,RPS6KA3,PRKCA |
|  | Role of Osteoblasts, Osteoclasts and Chondrocytes in Rheumatoid Arthritis | 2.28E00 | 2.09E-02 | BMPR1B,BMPR1A,IL1RN,SMAD6,MMP13 |
|  |  |  |  |  |
| **KAT5** | VDR/RXR Activation | 3.34E00 | 6.17E-02 | RUNX2,HES1 (includes EG:15205),CCL5,KLF4,PRKCA |
|  | Inositol Phosphate Metabolism | 2.75E00 | 3.03E-02 | PLCB4,INPP4B,CDK8,SYNJ1,SGK1,MAK |
|  | LPS/IL-1 Mediated Inhibition of RXR Function | 2.69E00 | 2.97E-02 | GSTT2/GSTT2B,ACSL3,IL1RN,ALDH1A3,CYP3A7,FMO5,ABCC4  (includes EG:10257) |
|  | Xenobiotic Metabolism Signaling | 2.64E00 | 2.71E-02 | GSTT2/GSTT2B,CYP1A2,ALDH1A3,CYP3A7,MAF,UGT2B15,FMO5,PRKCA |
|  | Chemokine Signaling | 2.6E00 | 5.48E-02 | PLCB4,CXCR4,CCL5,PRKCA |
|  | Metabolism of Xenobiotics by Cytochrome P450 | 2.6E00 | 2.55E-02 | GSTT2/GSTT2B,CYP1A2,ALDH1A3,CYP3A7,UGT2B15 |
|  | Nicotinate and Nicotinamide Metabolism | 2E00 | 2.9E-02 | CDK8,SGK1,ENPP5,MAK |
|  | Pantothenate and CoA Biosynthesis | 1.98E00 | 3.28E-02 | DPYSL2,ENPP5 |
|  | NRF2-mediated Oxidative Stress Response | 1.79E00 | 2.62E-02 | GSTT2/GSTT2B,MAF,DNAJB9,ABCC4 (includes EG:10257),PRKCA |
|  | Fatty Acid Metabolism | 1.75E00 | 2.19E-02 | ACSL3,CYP1A2,ALDH1A3,CYP3A7 |

Top 10 pathways most significantly associated with each gene set are shown.

| **Supplementary File 3D.** Ingenuity Pathway Analysis of IRF1- and STAT3-dependent androgen-responsive gene signature | | | | | | | |
| --- | --- | --- | --- | --- | --- | --- | --- |
| **Category** | **Functions Annotation** | **p-Value** | **Predicted Activation State** | **Regulation z-score** | **Genes** | **Number of genes** |  |
| Cell Death and Survival | apoptosis | 2.38E-11 | Decreased | -2.272 | ABCA1,ALDH1A3,ANG,ANGPT2,AR,ATF3,ATG3,BARD1,BHLHE40,BIRC3,BUB1,CCNA2,CDC20,CDC45,CDK1,CDK8,CDKN3,CENPF,CNN2,CRYAB,CTBP1,CXCL10,DHCR24,EAF2,ERN1,F2RL1,FAM134B,FIGNL1,FKBP5,GNMT,GNRH1,GPER1,GRB10,HLA-DMA,HMMR,HMOX1,HPGD,ID2,ID3,IL1RN,IL32,ITGAV,KCNMA1,KLF6,KLK3,KRT8,LAPTM4B,LDLR,LMNB1,MALT1,MCM2,MELK,MICAL1,MT2A,MTMR9,MYB,NAMPT,NDRG1,NFKB2,NFKBIA,NPC1,NUSAP1,ODC1,PBK,PEG3,PHLDA2,PIK3IP1,PMEPA1,PRKCH,PRKD1,PTCRA,PTGER4,PTTG1,RAB32,RASD1,RCAN1,RLN2,RRAS,SALL2,SAT1,SELENBP1,SEPP1,SERPINA3,SERPINI1,SGK1,SLC2A3,SLC39A10,SNAI2,SNRK,SOCS2,SPDEF,SPOCK1,STK3,TFF3,TK1,TNFAIP3,TNFAIP8,TOP2A,TOX3,TP53INP1,TPM1,TPX2,UBD,VEGFA,ZBTB16 | 105 |  |
| Cancer, Organismal Injury and Abnormalities | tumorigenesis of tissue | 4.21E-11 | Decreased | -2.145 | ABCA1,ABCC4,ABHD3,ACOT9,ACSL3,ACTA2,ACTG2,ADAM7,ADRA2A,AFF3,AGR2,AHNAK,AIDA,ALDH1A3,ALDH4A1,ANGPT2,ANXA9,AR,ASAP2,ASF1B,ASRGL1,ATG3,ATP1B1,AUTS2,AZGP1,BARD1,BCAP29,BCHE,BHLHE40,BICD2,BIRC3,BUB1,CADPS2,CAMK2N1,CBLL1,CBWD3/CBWD6,CCDC14,CCL8,CCNA2,CCNB2,CD68,CDC20,CDC45,CDCA3,CDCA5,CDH26,CDK1,CDK8,CDKN2B,CDKN3,CECR6,CEL,CENPF,CENPN,CKAP2L,CNN2,CNTNAP2,COL5A2,COLEC12,CORO2A,CRYAB,CSGALNACT1,CSMD1,CTBP1,CXCL10,CYP2U1,CYTH1,DDC,DHCR24,DIO1,DLGAP5,DNALI1,DPYSL2,DUSP5,EAF2,EFHC2,ELL2,ELOVL5,ERN1,ERRFI1,F2RL1,F5,FADS2,FAM103A1,FAM105A,FAM134B,FAM174B,FAM83D,FDFT1,FHDC1,FIGNL1,FKBP5,FMO4,FOXC1,FOXD4,FOXD4L1,FZD5,GATA2,GBP1,GLRX2,GNMT,GNRH1,GRB10,GSTT2/GSTT2B,HERC5,HES6,HMGB2,HMGCS1,HMMR,HMOX1,HOXC13,HPGD,ID2,ID3,IL10RB,IL1RN,IL32,INPP4B,INSIG1,IRX3,ITGAV,KCNMA1,KCNU1,KDELR3,KIFC1,KLF6,KLK2,KLK3,KLK4,KRT19,KRT6B,KRT6C,KRT8,LAMC1,LAPTM4B,LDLR,LIFR,LIN7B,LMNB1,LONRF1,LPAR3,LPIN1,LRRC31,LRRN1,LTN1,LXN,MAF,MALL,MALT1,MANEA,MAST4,MBOAT2,MCM2,MELK,MICAL1,MIPEP,MMP16,MPHOSPH9,MPZL1,MRPS18A,MT2A,MTMR9,MYB,MYBPC1,MYT1,NAMPT,NCAPD3,NCAPG,NDRG1,NETO1,NFIX,NFKBIA,NIPSNAP3A,NKX3.1,NMU,NOSTRIN,NPC1,NUP93,NUSAP1,ODC1,OPRK1,ORM1,ORM2,PAK1IP1,PBK,PCED1B,PDIA5,PEG3,PEX10,PGC,PGM3,PHLDA2,PKIB,PLA2G2A,PLEKHB2,PLPP1,PMEPA1,PNKD,PPFIBP2,PRC1,PRKCH,PRKD1,PTCRA,PTGER4,PTPN21,PTPRM,PTTG1,RAB32,RASD1,RER1,RHOU,RLN2,RRAS,S100P,SALL2,SASH1,SAT1,SELENBP1,SEMA6A,SEPP1,SERPINA3,SERPINI1,SGK1,SIPA1L2,SLC16A3,SLC16A6,SLC2A3,SLC39A10,SLC44A1,SLC45A3,SLITRK3,SMS,SNAI2,SNRK,SOCS2,SORD,SPOCK1,ST6GALNAC1,ST7,STEAP4,STK3,STK39,STXBP5L,SUSD6,TACSTD2,TFF3,TGM3,TK1,TM4SF1,TMEM144,TMEM38B,TMEM79,TMPRSS2,TMSB15A,TNFAIP3,TOP2A,TOX3,TP53INP1,TPM1,TPX2,TRIM48,TRPM8,TUBA3C/TUBA3D,TUBA3E,TULP4,UAP1,UBD,UBE2C,UGT2B11,UGT2B15,UGT2B28,VEGFA,VGF,WIPI1,ZBTB16,ZBTB43,ZNF350,ZNF462,ZNF812 | 279 |  |
| Organismal Survival | organismal death | 6.13E-11 | Decreased | -2.057 | ABCA1,ABCC4,ACKR3,ACTA2,ADGRG6,ADRA2A,AGR2,ALDH1A3,ANGPT2,AR,ATF3,ATG3,ATP1B1,BARD1,BCHE,BIRC3,BUB1,CADPS2,CCNA2,CCNB2,CD68,CDC20,CDC45,CDK1,CDKN2B,CDKN3,COL5A2,CRYAB,CTBP1,CXCL10,DHRS3,EAF2,ERN1,ERO1A,ERRFI1,F2RL1,F5,FADS2,FDFT1,FZD5,GATA2,HLA.DMA,HMOX1,HOXC13,HPGD,ID2,ID3,IL1RN,INSIG1,ITGAV,KCNMA1,KLF6,KRT19,KRT6B,KRT8,LAMC1,LAPTM4B,LDLR,LIFR,LIN7B,LMNB1,MAF,MCM2,MMP16,MYB,MYT1,NDRG1,NFIX,NFKB2,NFKBIA,NPC1,NUSAP1,PGM3,PLA2G2A,PRKCH,PRKD1,PTGER4,PTTG1,RAB3B,RCAN1,SALL2,SELENBP1,SEPP1,SLC2A3,SNAI2,SPDEF,ST7,STK3,SUSD6,TK1,TMEM38B,TNFAIP3,TOP2A,TP53INP1,UBD,VEGFA,VGF | 97 |  |
| Cancer, Gastrointestinal Disease, Organismal Injury and Abnormalities | digestive system cancer | 1.95E-10 | Decreased | -2.559 | ABCA1,ABCC4,ABHD3,ACKR3,ACOT9,ACTA2,ACTG2,ADAM7,ADRA2A,AFF3,AGR2,AHNAK,AIDA,ALDH1A3,ALDH4A1,ANG,ANGPT2,ANXA9,AR,ASAP2,ASF1B,ASRGL1,ATF3,AUTS2,AZGP1,BARD1,BCAP29,BCHE,BHLHE40,BICD2,BIRC3,BUB1,CADPS2,CAMK2N1,CBLL1,CBWD3/CBWD6,CCDC14,CCL8,CCNA2,CCNB2,CD68,CDC20,CDC45,CDCA3,CDCA5,CDH26,CDK1,CDK8,CDKN2B,CDKN3,CECR6,CEL,CENPF,CENPN,CKAP2L,CNTNAP2,COL5A2,COLEC12,CORO2A,CRYAB,CSGALNACT1,CSMD1,CTBP1,CXCL10,CYP2U1,CYTH1,DDC,DHCR24,DIO1,DLGAP5,DNALI1,DPYSL2,DUSP5,EAF2,EFHC2,ELL2,ELOVL5,EMP1,ERN1,ERRFI1,F2RL1,F5,FADS2,FAM83D,FDFT1,FHDC1,FIGNL1,FKBP5,FZD5,GATA2,GBP1,GLRX2,GNMT,GRB10,HERC5,HES6,HMGB2,HMGCS1,HMMR,HMOX1,HOXC13,HSD17B11,ID2,ID3,IL1RN,IL32,INPP4B,INSIG1,IRX3,ITGAV,KCNMA1,KCNU1,KDELR3,KIFC1,KLF6,KLK2,KLK3,KLK4,KRT19,KRT6B,KRT6C,KRT8,LAMC1,LAPTM4B,LDLR,LIFR,LIN7B,LONRF1,LPAR3,LRRC31,LRRN1,LTN1,MAF,MALL,MALT1,MANEA,MAST4,MBOAT2,MCM2,MELK,MICAL1,MIPEP,MMP16,MPHOSPH9,MPZL1,MRPS18A,MT2A,MTMR9,MYB,MYBPC1,MYT1,NAMPT,NCAPD3,NCAPG,NDRG1,NETO1,NFIX,NFKBIA,NKX3.1,NOSTRIN,NPC1,NUP93,NUSAP1,ODC1,OPRK1,ORM1,ORM2,PBK,PCED1B,PDIA5,PEG3,PEX10,PGC,PGM3,PHLDA2,PIK3IP1,PLA2G2A,PLEKHB2,PMEPA1,PNKD,PPFIBP2,PRC1,PRKCH,PRKD1,PTCRA,PTGER4,PTPN21,PTPRM,PTTG1,RAB32,RASD1,RER1,RLN2,S100P,SALL2,SASH1,SAT1,SELENBP1,SEMA6A,SEPP1,SERPINA3,SERPINI1,SGK1,SIPA1L2,SLC16A3,SLC16A6,SLC2A3,SLC39A10,SLC45A3,SLITRK3,SMS,SNAI2,SNRK,SOCS2,SPOCK1,ST6GALNAC1,STEAP4,STK3,STK39,STXBP5L,SUSD6,TACSTD2,TFF3,TGM3,TK1,TM4SF1,TMEM144,TMEM38B,TMEM79,TMPRSS2,TMSB15A,TNFAIP3,TOP2A,TOX3,TP53INP1,TPM1,TPX2,TRIM48,TRPM8,TUBA3C/TUBA3D,TUBA3E,TULP4,UAP1,UBD,UBE2C,UGT2B15,UGT2B28,VEGFA,VGF,WIPI1,ZBTB16,ZBTB43,ZNF350,ZNF462 | 254 |  |
| Cancer, Organismal Injury and Abnormalities | neoplasia of epithelial tissue | 5.99E-10 | Decreased | -2.008 | ABCA1,ABCC4,ABHD3,ACOT9,ACSL3,ACTA2,ACTG2,ADAM7,ADRA2A,AFF3,AGR2,AHNAK,AIDA,ALDH1A3,ALDH4A1,ANGPT2,ANXA9,AR,ASAP2,ASF1B,ASRGL1,ATG3,ATP1B1,AUTS2,AZGP1,BARD1,BCAP29,BCHE,BHLHE40,BICD2,BUB1,CADPS2,CAMK2N1,CBLL1,CBWD3/CBWD6,CCDC14,CCL8,CCNA2,CCNB2,CD68,CDC20,CDC45,CDCA3,CDCA5,CDH26,CDK1,CDK8,CDKN2B,CDKN3,CECR6,CEL,CENPF,CENPN,CKAP2L,CNN2,CNTNAP2,COL5A2,COLEC12,CORO2A,CRYAB,CSGALNACT1,CSMD1,CTBP1,CXCL10,CYP2U1,CYTH1,DDC,DHCR24,DIO1,DLGAP5,DNALI1,DPYSL2,DUSP5,EAF2,EFHC2,ELL2,ELOVL5,ERN1,ERRFI1,F2RL1,F5,FADS2,FAM103A1,FAM105A,FAM134B,FAM174B,FAM83D,FDFT1,FHDC1,FIGNL1,FKBP5,FMO4,FOXC1,FOXD4,FOXD4L1,FZD5,GATA2,GBP1,GLRX2,GNMT,GNRH1,GRB10,GSTT2/GSTT2B,HERC5,HES6,HMGB2,HMGCS1,HMMR,HMOX1,HOXC13,HPGD,ID2,ID3,IL1RN,IL32,INPP4B,INSIG1,IRX3,ITGAV,KCNMA1,KCNU1,KDELR3,KIFC1,KLF6,KLK2,KLK3,KLK4,KRT19,KRT6B,KRT6C,KRT8,LAMC1,LAPTM4B,LDLR,LIFR,LIN7B,LMNB1,LONRF1,LPAR3,LPIN1,LRRC31,LRRN1,LTN1,LXN,MAF,MALL,MALT1,MANEA,MAST4,MBOAT2,MCM2,MELK,MICAL1,MIPEP,MMP16,MPHOSPH9,MPZL1,MRPS18A,MT2A,MTMR9,MYB,MYBPC1,MYT1,NAMPT,NCAPD3,NCAPG,NETO1,NFIX,NFKBIA,NKX3.1,NMU,NOSTRIN,NPC1,NUP93,NUSAP1,ODC1,OPRK1,ORM1,ORM2,PAK1IP1,PBK,PCED1B,PDIA5,PEG3,PEX10,PGC,PGM3,PHLDA2,PKIB,PLA2G2A,PLEKHB2,PLPP1,PMEPA1,PNKD,PPFIBP2,PRC1,PRKCH,PRKD1,PTCRA,PTGER4,PTPN21,PTPRM,PTTG1,RAB32,RASD1,RER1,RHOU,RLN2,RRAS,S100P,SALL2,SASH1,SAT1,SELENBP1,SEMA6A,SEPP1,SERPINA3,SERPINI1,SGK1,SIPA1L2,SLC16A3,SLC16A6,SLC2A3,SLC39A10,SLC44A1,SLC45A3,SLITRK3,SMS,SNAI2,SNRK,SOCS2,SORD,SPOCK1,ST6GALNAC1,STEAP4,STK3,STK39,STXBP5L,SUSD6,TACSTD2,TFF3,TGM3,TK1,TM4SF1,TMEM144,TMEM38B,TMEM79,TMPRSS2,TMSB15A,TNFAIP3,TOP2A,TOX3,TP53INP1,TPM1,TPX2,TRIM48,TRPM8,TUBA3C/TUBA3D,TUBA3E,TULP4,UAP1,UBD,UBE2C,UGT2B11,UGT2B15,UGT2B28,VEGFA,VGF,WIPI1,ZBTB16,ZBTB43,ZNF350,ZNF462,ZNF812 | 274 |  |
| Cell Death and Survival | apoptosis of tumor cell lines | 6.35E-09 | Decreased | -2.628 | ANGPT2,AR,ATF3,BARD1,BHLHE40,BIRC3,BUB1,CDC20,CDK1,CDK8,CNN2,CRYAB,CTBP1,DHCR24,EAF2,ERN1,FKBP5,GNRH1,GRB10,HMMR,HMOX1,ID3,IL32,ITGAV,KLF6,KLK3,LMNB1,MALT1,MELK,MT2A,MTMR9,MYB,NDRG1,NFKB2,NFKBIA,ODC1,PBK,PMEPA1,PRKD1,PTTG1,RAB32,RASD1,RCAN1,SALL2,SAT1,SERPINA3,SGK1,SNAI2,SPOCK1,STK3,TFF3,TNFAIP3,TNFAIP8,TOP2A,TPX2,VEGFA,ZBTB16 | 57 |  |
| Organismal Development | size of body | 3.09E-07 | Increased | 2.050 | ABCA1,ADGRG6,AGR2,AHNAK,AR,CADPS2,CCNB2,CNTNAP2,COL5A2,CSGALNACT1,CTBP1,DHRS3,FADS2,GPER1,GRB10,HLA-DMA,HMOX1,ID2,IL1RN,INSIG1,KCNMA1,KLF6,KRT6B,KRT8,LAMC1,MAF,MMP16,NDRG1,NFIX,NFKB2,NFKBIA,PEG3,PRKCH,PTTG1,RAB3B,RCAN1,SNAI2,SOCS2,TNFAIP3,VEGFA,VGF | 41 |  |
| Digestive System Development and Function, Hepatic System Development and Function, Organ Morphology | mass of liver | 1.30E-06 | Decreased | -2.111 | CEL,FADS2,FDFT1,GNMT,GPER1,KLF6,LDLR,ODC1,PLA2G2A,SAT1,SELENBP1,STEAP4 | 12 |  |
| Cell Death and Survival | cell death of connective tissue cells | 1.32E-06 | Decreased | -2.847 | ABCC4,AR,ATF3,ATG3,BIRC3,BUB1,CDC20,CDK1,CTBP1,DHCR24,GRB10,HMOX1,ID2,ITGAV,KLF6,KRT8,MELK,MICAL1,NFKB2,NFKBIA,NPC1,PEG3,S100P,SNAI2,STK3,TNFAIP3,TNFAIP8,TOP2A,TP53INP1,UBD,VEGFA | 31 |  |
| Immunological Disease | hypersensitive reaction | 3.26E-06 | Increased | 2.138 | ABCA1,ADRA2A,AHNAK,ANGPT2,ATP1B1,CCNA2,CXCL10,DPYSL2,F2RL1,FADS2,HLA.DMA,IL1RN,KLK4,KRT6C,MALT1,NFKBIA,OPRK1,PGM3,PTGER4,SOCS2,TGM3,TNFAIP3,TPM1,VEGFA | 24 |  |
| Gastrointestinal Disease, Hepatic System Disease, Organismal Injury and Abnormalities | liver lesion | 2.20E-05 | Decreased | -2.052 | ABCA1,ABCC4,ABHD3,ACTA2,ACTG2,ADRA2A,AFF3,AHNAK,ALDH1A3,ALDH4A1,ANG,ANGPT2,ANXA9,AR,ASAP2,ASF1B,ASRGL1,AUTS2,BCAP29,BCHE,BHLHE40,BICD2,BIRC3,BUB1,CADPS2,CAMK2N1,CCL8,CCNA2,CCNB2,CDC20,CDC45,CDCA3,CDH26,CDK8,CDKN2B,CDKN3,CENPF,CNTNAP2,COL5A2,CORO2A,CSMD1,CXCL10,CYTH1,DDC,DHCR24,EAF2,EFHC2,ELL2,ELOVL5,ERRFI1,F2RL1,F5,FAM83D,FHDC1,GNMT,GRB10,HES6,HMGCS1,HMMR,HMOX1,HPGD,ID3,IL10RB,IL1RN,INPP4B,INSIG1,ITGAV,KCNMA1,KIFC1,KRT8,LAMC1,LAPTM4B,LDLR,LIFR,LPIN1,LRRC31,LTN1,MALT1,MAST4,MMP16,MPHOSPH9,MRPS18A,MT2A,MTMR9,NCAPD3,NCAPG,NDRG1,NETO1,NFIX,NFKBIA,NPC1,NUSAP1,ODC1,OPRK1,PEG3,PIK3IP1,PMEPA1,PRC1,PRKCH,PRKD1,PTPN21,PTTG1,RER1,SALL2,SEMA6A,SEPP1,SGK1,SIPA1L2,SLC16A6,SLC2A3,SLC39A10,SLC45A3,SLITRK3,SNAI2,SNRK,STK3,STXBP5L,TACSTD2,TGM3,TK1,TMEM38B,TMEM79,TMPRSS2,TNFAIP3,TOP2A,TOX3,TPX2,TUBA3C/TUBA3D,TULP4,UAP1,UBE2C,UGT2B15,VEGFA,ZBTB16,ZBTB43,ZNF462 | 136 |  |
| Hematological System Development and Function, Tissue Morphology | quantity of leukocytes | 7.67E-05 | Increased | 2.177 | ABCA1,ACKR3,AGR2,ATF3,BHLHE40,BIRC3,BUB1,CCNB2,CDKN2B,CNN2,CXCL10,F2RL1,GATA2,GNMT,GNRH1,HLA.DMA,HMOX1,ID2,ID3,IL10RB,IL1RN,LDLR,MAF,MALT1,MYB,NFKB2,NFKBIA,NPC1,PRKCH,PTCRA,PTTG1,SLC39A10,SNAI2,STEAP4,TNFAIP3,VEGFA,ZBTB16 | 37 |  |
| Cancer, Organismal Injury and Abnormalities | neoplasia of cells | 7.96E-05 | Increased | 2.318 | ABCA1,ABCC4,AFF3,AGR2,AHNAK,AIDA,ALDH1A3,ANG,ANGPT2,AR,ATF3,ATP1B1,BICD2,CADPS2,CBWD3/CBWD6,CCL8,CDC45,CDCA5,CEL,CNN2,CNTNAP2,CSMD1,CXCL10,DDC,DUSP5,EFHC2,ERN1,F5,FAM105A,FAM134B,FHDC1,FMO4,FOXC1,FOXD4,FOXD4L1,GRB10,GSTT2/GSTT2B,HMOX1,IL1RN,IL32,INPP4B,ITGAV,KCNMA1,KCNU1,KLF6,KLK2,KRT6B,KRT6C,LAPTM4B,LDLR,LIFR,LTN1,MANEA,MAST4,MMP16,MRPS18A,MYB,MYBPC1,MYT1,NDRG1,NFIX,NFKBIA,NMU,NUSAP1,ODC1,ORM1,PAK1IP1,PEG3,PGC,PGM3,PLEKHB2,PLPP1,PNKD,PRKD1,PTGER4,PTPN21,PTPRM,PTTG1,RHOU,SASH1,SEMA6A,SERPINA3,SERPINI1,SLC16A6,SLC2A3,SLC45A3,SLITRK3,SNAI2,SNRK,SOCS2,SORD,STEAP4,STXBP5L,TACSTD2,TGM3,TM4SF1,TMEM144,TMEM38B,TNFAIP3,TOP2A,TPM1,TPX2,TUBA3C/TUBA3D,TUBA3E,TULP4,UBD,UGT2B11,UGT2B28,VEGFA,VGF,ZBTB16,ZNF350,ZNF462,ZNF812 | 114 |  |
| Digestive System Development and Function, Hepatic System Development and Function, Organ Development | response of liver | 1.24E-04 | Decreased | -2.008 | ACTA2,BIRC3,CCL8,CXCL10,GNMT,HMOX1,IL10RB,IL1RN,KRT8,LDLR,LPIN1,NPC1,OPRK1,TK1,TNFAIP3,ZBTB16 | 16 |  |
| Hematological System Development and Function, Tissue Morphology | quantity of blood cells | 2.05E-04 | Increased | 2.533 | ABCA1,ACKR3,AGR2,ATF3,BHLHE40,BIRC3,BUB1,CCNB2,CDKN2B,CNN2,CXCL10,F2RL1,GATA2,GNMT,GNRH1,HLA.DMA,HMOX1,ID2,ID3,IL10RB,IL1RN,LDLR,MAF,MALT1,MYB,NFKB2,NFKBIA,NMU,NPC1,PRKCH,PTCRA,PTTG1,RCAN1,SLC39A10,SNAI2,STEAP4,TNFAIP3,VEGFA,ZBTB16 | 39 |  |
| Cancer, Organismal Injury and Abnormalities | cancer of cells | 2.60E-04 | Increased | 2.003 | ABCA1,ABCC4,AFF3,AGR2,AHNAK,AIDA,ANGPT2,AR,ATF3,ATP1B1,BICD2,CADPS2,CBWD3/CBWD6,CCL8,CDC45,CDCA5,CEL,CNN2,CNTNAP2,CSMD1,CXCL10,DDC,DUSP5,EFHC2,ERN1,F5,FAM105A,FAM134B,FHDC1,FMO4,FOXC1,FOXD4,FOXD4L1,GRB10,GSTT2/GSTT2B,HMOX1,IL1RN,IL32,INPP4B,ITGAV,KCNMA1,KCNU1,KLK2,KRT6B,KRT6C,LDLR,LIFR,LTN1,MANEA,MAST4,MMP16,MRPS18A,MYB,MYBPC1,MYT1,NDRG1,NFIX,NFKBIA,NMU,NUSAP1,ODC1,ORM1,PAK1IP1,PEG3,PGC,PGM3,PLEKHB2,PNKD,PRKD1,PTGER4,PTPN21,PTPRM,PTTG1,RHOU,SASH1,SEMA6A,SERPINA3,SERPINI1,SLC16A6,SLC2A3,SLC45A3,SLITRK3,SNAI2,SNRK,SOCS2,SORD,STEAP4,STXBP5L,TACSTD2,TGM3,TM4SF1,TMEM144,TMEM38B,TNFAIP3,TOP2A,TPM1,TPX2,TUBA3C/TUBA3D,TUBA3E,TULP4,UBD,UGT2B11,UGT2B28,VEGFA,VGF,ZBTB16,ZNF350,ZNF462,ZNF812 | 109 |  |
| Cell Death and Survival | cell death of fibroblast cell lines | 6.04E-04 | Decreased | -2.662 | ABCC4,ATF3,ATG3,BIRC3,CDC20,CDK1,GRB10,HMOX1,ID2,KLF6,KRT8,MELK,MICAL1,NFKB2,NFKBIA,S100P,TNFAIP3,TOP2A,TP53INP1 | 19 |  |
| Cell Cycle | entry into interphase | 1.06E-03 | Decreased | -2.333 | BHLHE40,BUB1,CDC20,CDK1,CDKN2B,HMOX1,ID2,ID3,ZBTB16 | 9 |  |
| Cardiovascular System Development and Function, Organismal Development, Tissue Morphology | vasodilation of artery | 1.29E-03 | Decreased | -2.215 | CXCL10,GPER1,HMOX1,LDLR,RLN2,TMBIM1 | 6 |  |
| Cardiovascular System Development and Function, Hematological System Development and Function | systolic pressure | 1.46E-03 | Increased | 2.000 | ACTA2,ADRA2A,ANGPT2,HMOX1,LDLR,STK39,VEGFA,VGF | 8 |  |
| Cell Death and Survival, Gastrointestinal Disease, Hepatic System Disease, Organismal Injury and Abnormalities | apoptosis of liver cells | 1.47E-03 | Decreased | -2.157 | BIRC3,CXCL10,ITGAV,KRT8,LDLR,NFKBIA,NPC1,SEPP1,STK3 | 9 |  |

**Supplementary File 3E.** Ingenuity Pathway Analysis of p53- and WDR77-dependent androgen-responsive gene signature

| **Category** | **Functions Annotation** | **p-Value** | **Predicted Activation State** | **Regulation z-score** | **Genes** | **Number of genes** |
| --- | --- | --- | --- | --- | --- | --- |
| Cell Death and Survival | cell death | 4.21E-06 | Decreased | -2.274 | ABCC4,ACKR3,AGR2,ALDH1A3,ANG,ANGPT2,ATF3,BARD1,BCAP29,BCHE,BHLHE40,CDK8,CTBP1,CXCL10,DHCR24,EAF2,F2RL1,F5,FDFT1,FKBP5,GATA2,GDF15,GNMT,GNRH1,GPER1,GRB10,HLA.DMA,HMGCR,HPGD,IQGAP2,ITGAV,KCNMA1,KLK3,KRT18,KRT19,KRT6A,KRT8,LDLR,LRIG1,MAF,MALT1,MERTK,MICAL1,MPHOSPH9,MTFP1,MTMR9,NDRG1,NKX3.1,NPC1,NUP93,ODC1,PHLDA2,PLA2G2A,PMEPA1,PRKCH,PRKD1,PTGER4,RASD1,RLN2,S100A11,S100P,SAT1,SCD,SELENBP1,SEMA6A,SEPP1,SERPINI1,SGK1,SH3RF1,SLC2A3,SLC39A10,SNAI2,SNRK,SOCS2,SPDEF,STK3,TP53INP1,TSC22D1,TXNIP | 79 |
| Organismal Survival | organismal death | 7.57E-06 | Decreased | -2.967 | ABCC4,ACKR3,ACTA2,ADGRG6,AGR2,ALDH1A3,ANGPT2,ATF3,ATP1B1,BARD1,BCHE,CTBP1,CXCL10,DBI,DHRS3,EAF2,ERO1A,ERRFI1,F2RL1,F5,FADS2,FDFT1,FZD5,GATA2,GDF15,HLA.DMA,HMGCR,HOXC13,HPGD,INSIG1,IQGAP2,ITGAV,KCNMA1,KRT19,KRT8,LDLR,MAF,MERTK,MMP16,MYT1,NDRG1,NPC1,PGM3,PITX1,PLA2G2A,PRKCH,PRKD1,PTGER4,RAB3B,SELENBP1,SEPP1,SGK223,SLC2A3,SNAI2,SPDEF,ST7,STK3,TMEM38B,TP53INP1,TXNIP,VTA1 | 61 |
| Cell Death and Survival | Necrosis | 3.90E-05 | Decreased | -2.261 | ABCC4,ACKR3,AGR2,ANG,ANGPT2,ATF3,BARD1,BCHE,BHLHE40,CDK8,CTBP1,CXCL10,DHCR24,EAF2,F2RL1,F5,FDFT1,FKBP5,GATA2,GDF15,GNMT,GNRH1,GRB10,HLA.DMA,IQGAP2,ITGAV,KLK3,KRT18,KRT8,LDLR,LRIG1,MALT1,MERTK,MICAL1,MPHOSPH9,MTFP1,MTMR9,NDRG1,NPC1,NUP93,ODC1,PMEPA1,PRKCH,PRKD1,PTGER4,RASD1,S100A11,S100P,SAT1,SCD,SEMA6A,SEPP1,SERPINI1,SGK1,SH3RF1,SLC39A10,SNAI2,SNRK,STK3,TP53INP1,TSC22D1,TXNIP | 62 |
| Cell Death and Survival | apoptosis | 8.49E-05 | Decreased | -2.202 | ALDH1A3,ANG,ANGPT2,ATF3,BARD1,BHLHE40,CDK8,CTBP1,CXCL10,DHCR24,EAF2,F2RL1,FKBP5,GDF15,GNMT,GNRH1,GPER1,GRB10,HLA-DMA,HMGCR,HPGD,IQGAP2,ITGAV,KCNMA1,KLK3,KRT18,KRT8,LDLR,LRIG1,MALT1,MERTK,MICAL1,MTFP1,MTMR9,NDRG1,NPC1,ODC1,PHLDA2,PMEPA1,PRKCH,PRKD1,PTGER4,RASD1,RLN2,S100A11,SAT1,SCD,SELENBP1,SEPP1,SERPINI1,SGK1,SH3RF1,SLC2A3,SLC39A10,SNAI2,SNRK,SOCS2,SPDEF,STK3,TP53INP1,TSC22D1,TXNIP | 62 |
| Cell Death and Survival, Gastrointestinal Disease, Hepatic System Disease, Organismal Injury and Abnormalities | apoptosis of liver cells | 1.02E-04 | Decreased | -2.360 | CXCL10,IQGAP2,ITGAV,KRT8,LDLR,NPC1,SCD,SEPP1,STK3 | 9 |
| Cell Death and Survival, Gastrointestinal Disease, Hepatic System Disease, Organismal Injury and Abnormalities | cell death of liver cells | 1.42E-04 | Decreased | -2.360 | CXCL10,IQGAP2,ITGAV,KRT8,LDLR,NPC1,PRKD1,SCD,SEPP1,STK3 | 10 |
| Cell Death and Survival, Gastrointestinal Disease, Hepatic System Disease, Organismal Injury and Abnormalities | necrosis of liver | 1.96E-04 | Decreased | -2.543 | CXCL10,GNMT,IQGAP2,ITGAV,KRT8,LDLR,NPC1,PRKD1,SCD,SEPP1,STK3 | 11 |
| Inflammatory Response | inflammation of body cavity | 2.81E-04 | Decreased | -2.139 | ABCC4,ACTA2,AGR2,ANGPT2,ATF3,CXCL10,F2RL1,F5,GNMT,HMGCR,INSIG1,KRT8,LDLR,MERTK,NPC1,OPRK1,PLA2G2A,PRKD1,SAT1,SCD,SELENBP1,SOCS2,SPDEF,TP53INP1 | 24 |
| Immunological Disease | delayed hypersensitive reaction | 8.42E-04 | Increased | 2.219 | ANGPT2,ATP1B1,CXCL10,F2RL1,HLA-DMA,KRT6C,PTGER4 | 7 |
| Cell Death and Survival | apoptosis of tumor cell lines | 1.36E-03 | Decreased | -2.113 | ANGPT2,ATF3,BARD1,BHLHE40,CDK8,CTBP1,DHCR24,EAF2,FKBP5,GDF15,GNRH1,GRB10,ITGAV,KLK3,KRT18,MALT1,MERTK,MTFP1,MTMR9,NDRG1,ODC1,PMEPA1,PRKD1,RASD1,S100A11,SAT1,SCD,SGK1,SH3RF1,SNAI2,STK3 | 31 |
| Organismal Development | size of body | 2.35E-03 | Increased | 2.362 | ADGRG6,AGR2,AHNAK,CNTNAP2,CSGALNACT1,CTBP1,DBI,DHRS3,FADS2,GDF15,GPER1,GRB10,HLA.DMA,INSIG1,KCNMA1,KRT8,MAF,MMP16,NDRG1,PRKCH,RAB3B,SNAI2,SOCS2 | 23 |
| Dermatological Diseases and Conditions | loss of hair | 2.37E-03 | Decreased | -2.000 | DBI,HOXC13,KLK3,LDLR,LRIG1,SCD | 6 |
| Cancer, Organismal Injury and Abnormalities | neoplasia of cells | 8.84E-03 | Increased | 2.785 | AFF3,AGR2,AHNAK,AIDA,ALDH1A3,ANG,ANGPT2,ATF3,ATP1B1,BICD2,C1orf116,CATSPER2,CNTNAP2,CXCL10,DBI,DDC,F5,FHDC1,FOXD4,GRB10,ITGAV,KCNMA1,KLK2,KRT18,KRT6C,LTN1,MANEA,MAST4,MMP16,MRPS18A,NDRG1,ODC1,ORM1,PAK1IP1,PGC,PGM3,PITX1,PLPP1,PRKD1,PTGER4,PTPN21,PTPRM,RHOU,SASH1,SERPINI1,SLC2A3,SLC45A3,SLITRK3,SNAI2,SNRK,SOCS2,SORD,STXBP5L,TM4SF1,TMEM38B,TSKU,TUBA3C/TUBA3D,TUBA3E,TULP4,TXNIP,UGT2B11,UGT2B28,ZNF462,ZNF812 | 64 |
| Organismal Survival | survival of organism | 1.01E-02 | Increased | 2.356 | ABCC4,ANGPT2,BCHE,CXCL10,F2RL1,FZD5,IQGAP2,KRT18,KRT19,LDLR,LRRN1,NKX3-1,NPC1,ODC1,ORM1,ORM2,PLA2G2A,PTGER4 | 18 |
