# Supplementary material for: A comprehensive analysis of coregulator recruitment, androgen receptor function and gene expression in prostate cancer
Source: eLife. 2017 Aug 18;6:e28482. doi: 10.7554/eLife.28482 (PMC5608510; doi:10.7554/eLife.28482)
Supplement: Supplementary file 4. — (A) Real-time RT-PCR validation of siRNA-mediated coregulator knock-out efficiency LNCaP cells were transfected with On Target siRNA SmartPools directed against 22 coregulators, against AR, or with non-targeting On Target Plus control SmartPool. At 96 hr after transfection, cells were harvested and RNA was extracted for real-time RT-PCR analysis. Target gene mRNA levels were normalized to GAPDH expression and are expressed as relative expression, taking the value obtained from one of the control siRNA-transfected conditions as 1. Columns, means of values obtained from three independent biological replicates; bars, sem. Gray bars, control siRNA-transfected cells; Black bars, specific siRNA-transfected cells (B) Morphology of LNCaP cells following coregulator silencing LNCaP cells were transfected with individual On Target siRNA SmartPools directed against each of the 22 coregulators, against AR, or a non-targeting On Target Plus control SmartPool. At 96 hr after transfection, cells were imaged using Infinity Analyze software. (C) Effects of coregulator silencing on AR protein expression levels LNCaP cells were transfected with individual On Target siRNA SmartPools directed against each of the 22 coregulators or against AR, or with non-targeting On Target Plus control SmartPools (c). At 96 hr after transfection, cells were harvested and total protein extracts were subjected to western blot analysis using an antibody directed against AR. To control for potential loading differences, blots were stripped and reprobed with an antibody targeted at β-actin. [file elife-28482-supp4.pdf]

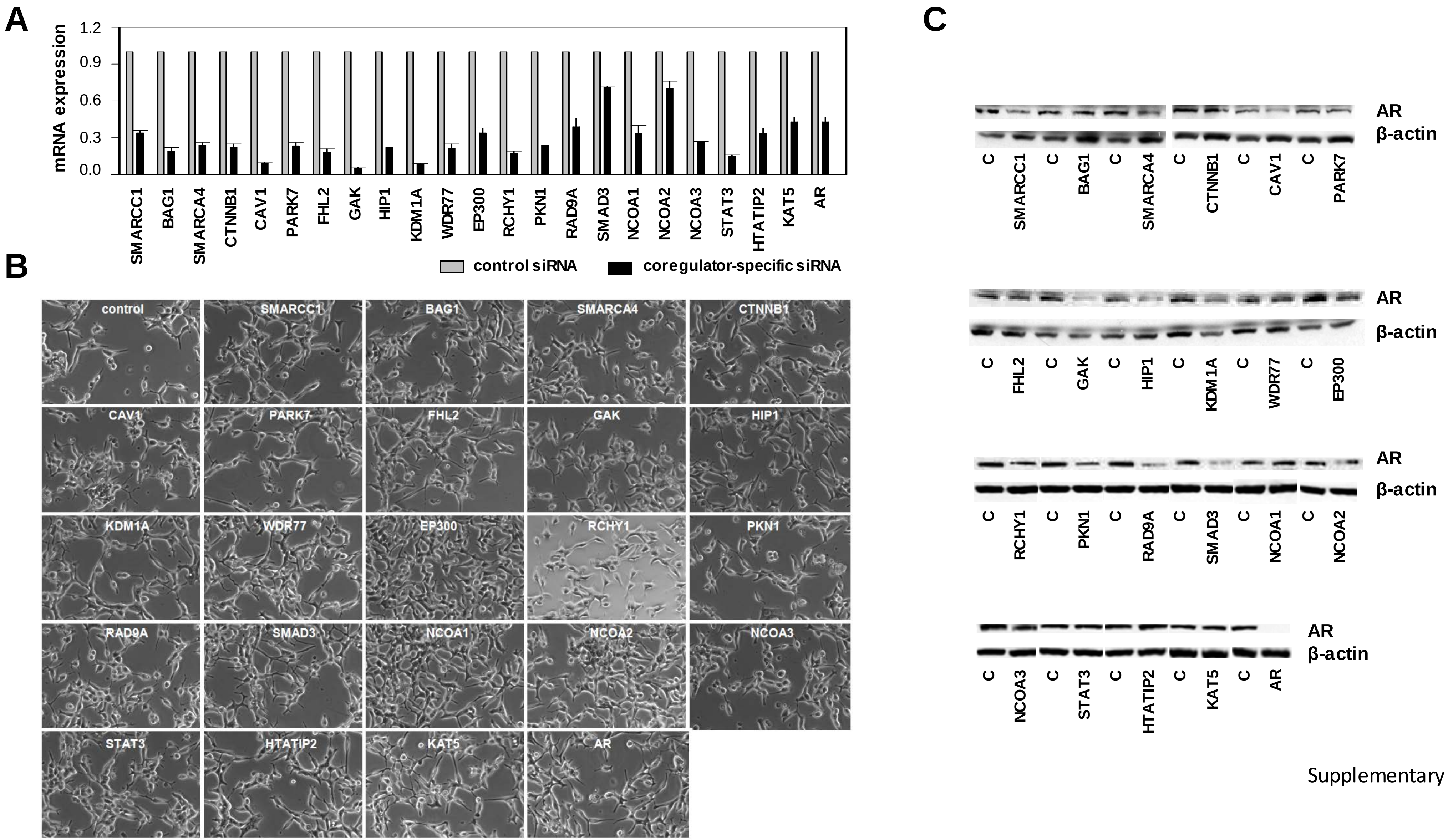

Supplementary File 4

Supplementary File 4. Impact of silencing of 22 clinically relevant coregulators on prostate cancer cells and AR expression

A. Real-time RT-PCR validation of siRNA-mediated coregulator knock-out efficiency

LNCaP cells were transfected with On Target siRNA SmartPools directed against 22 coregulators, against AR, or with non-targeting On Target Plus control SmartPool. At 96 hours after transfection, cells were harvested and RNA was extracted for real-time RT-PCR analysis. Target gene mRNA levels were normalized to GAPDH expression and are expressed as relative expression, taking the value obtained from one of the control siRNA-transfected conditions as 1. *Columns*, means of values obtained from three independent biological replicates; *bars*, sem. Gray bars, control siRNA-transfected cells; Black bars, specific siRNA-transfected cells

B. Morphology of LNCaP cells following coregulator silencing

LNCaP cells were transfected with individual On Target siRNA SmartPools directed against each of the 22 coregulators, against AR, or a non-targeting On Target Plus control SmartPool. At 96 hours after transfection, cells were imaged using Infinity Analyze software.

C. Effects of coregulator silencing on AR protein expression levels

LNCaP cells were transfected with individual On Target siRNA SmartPools directed against each of the 22 coregulators or against AR, or with non-targeting On Target Plus control SmartPools (c). At 96 hours after transfection, cells were harvested and total protein extracts were subjected to western blot analysis using an antibody directed against AR. To control for potential loading differences, blots were stripped and reprobed with an antibody targeted at β-actin.
