# Supplementary material for: A comprehensive analysis of coregulator recruitment, androgen receptor function and gene expression in prostate cancer
Source: eLife. 2017 Aug 18;6:e28482. doi: 10.7554/eLife.28482 (PMC5608510; doi:10.7554/eLife.28482)
Supplement: Supplementary file 5. — (A) Nomenclature used to describe the effect of loss of coregulator on androgen regulation of AR target gene expression co+, androgen regulation is more pronounced (+) after loss of coregulator expression and the direction of androgen regulation remains consistent (co); co-, androgen regulation is less pronounced (-) after loss of coregulator expression and the direction of androgen regulation remains consistent (co); op+, androgen regulation is more pronounced (+) after loss of coregulator expression and the direction of androgen regulation is opposite (op); op-, as androgen regulation is less pronounced (-) after loss of coregulator expression and direction of androgen regulation is opposite (op). Androgen-induced, gene which expression is increased in control siRNA (c)-transfected cells after androgen stimulation; androgen-repressed gene which expression is decreased in control siRNA (c)-transfected cells after androgen stimulation. Spec, specific for one coregulator (B) Overview of coregulators that affect androgen regulation of the genes encoding RAB27A and GNB4 (C) Experimental validation of recruitment of 6 representative coregulators to the genes encoding RAB27A and GNB4 ChIP studies verifying recruitment of 6 coregulators at AREs within the genes encoding RAB27A and GNB4. ChIP was done on LNCaP cells that had been treated with 5nM R1881 or vehicle for 16 hr. Coregulators analyzed via ChIP were chosen based on availability of suitable antibodies. (D) Time course of androgen-dependent recruitment of AR (left), NCOA3 (middle) and WDR77 (right) to AREs in the genes encoding RAB27A and GNB4 ChIP was done on LNCaP cells that had been treated with 5nM R1881 or vehicle for 1, 4, 16 or 48 hr. (E) Impact of knock-down of WDR77 or NCOA3 on kinetics of androgen-regulation of RAB27A and GNB4 LNCaP cells were transfected with On Target siRNA SmartPools directed against WDR77 or NCOA3, or with non-targeting On Target Plus control SmartPool (c). At 42 hr after transfection [file elife-28482-supp5.pdf]

A

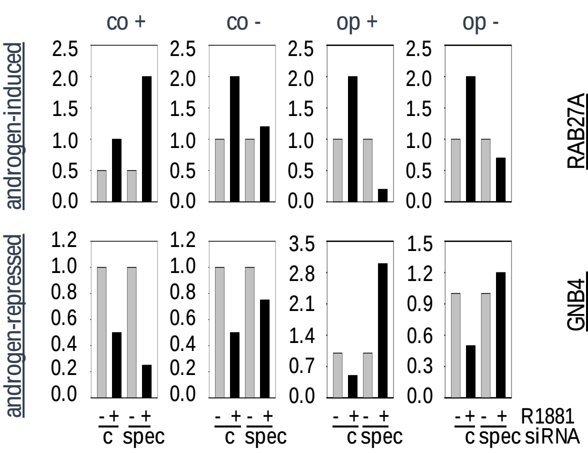

C

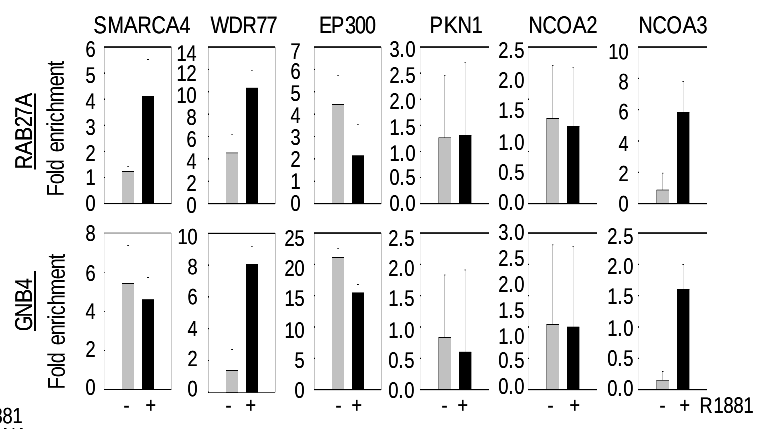

B

	SMARCC1	BAG1	SMARCA4	CTNNB1	CAV1	PARK7	FHL2	KDM1A	WDR77	EP300	RCHY1	PKN1	NCOA1	NCOA2	NCOA3	STAT3	HTATIP2	KAT5
RAB27A	co+	0	co+	co+	co+	co+	0	co+	co+	co-	0	0	co+	0	co+	co+	co+	0
GNB4	0	0	0	0	0	0	0	0	co+	0	0	0	0	0	co+	co-	co+	0

D

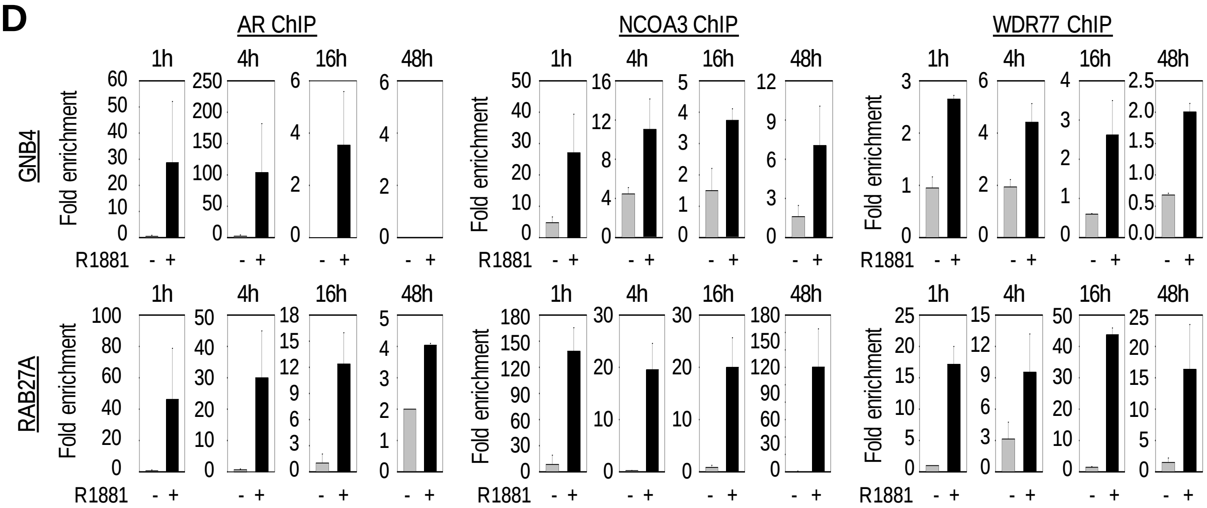

E

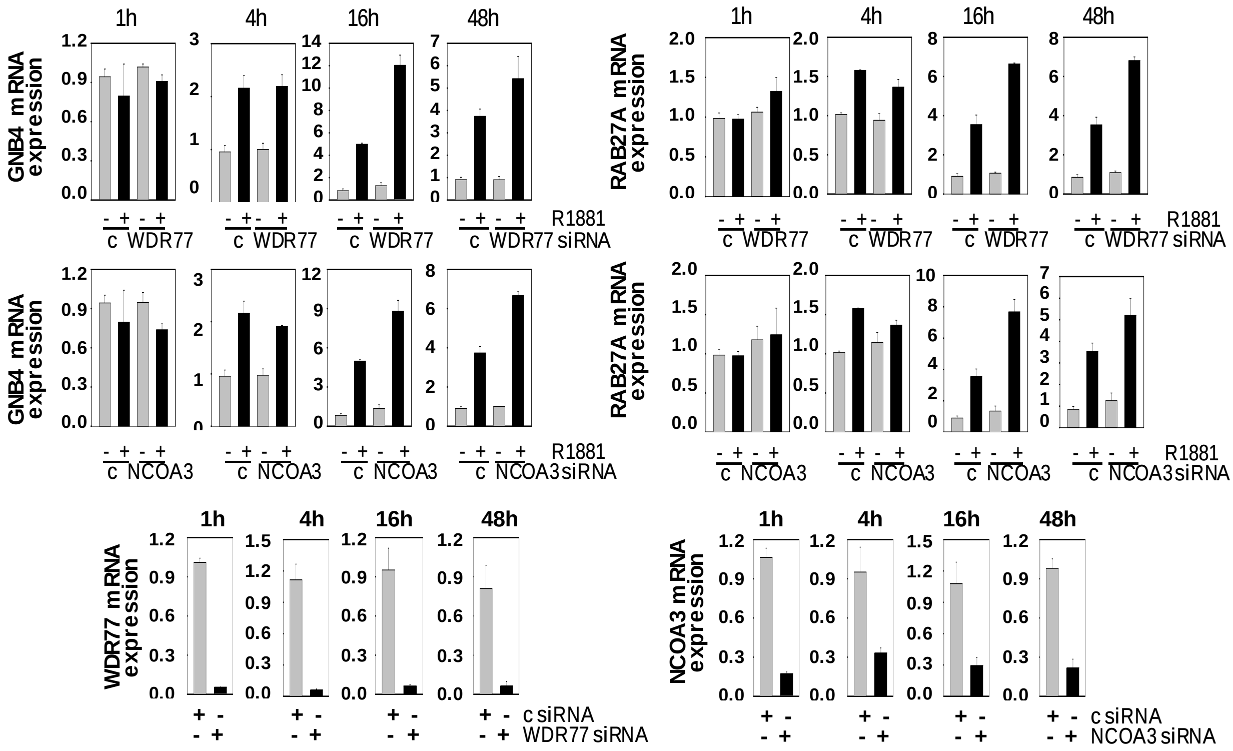

Supplementary File 5

Supplementary File 5. Impact of coregulator silencing on androgen regulation of AR target genes

A. Nomenclature used to describe the effect of loss of coregulator on androgen regulation of AR target gene expression

co+, androgen regulation is more pronounced (+) after loss of coregulator expression and the direction of androgen regulation remains consistent (co); co-, androgen regulation is less pronounced (-) after loss of coregulator expression and the direction of androgen regulation remains consistent (co); op+, androgen regulation is more pronounced (+) after loss of coregulator expression and the direction of androgen regulation is opposite (op); op-, as androgen regulation is less pronounced (-) after loss of coregulator expression and direction of androgen regulation is opposite (op). Androgen-induced, gene which expression is increased in control siRNA (c)-transfected cells after androgen stimulation; androgen-repressed gene which expression is decreased in control siRNA (c)-transfected cells after androgen stimulation. Spec, specific for one coregulator

B. Overview of coregulators that affect androgen regulation of the genes encoding RAB27A and GNB4

C. Experimental validation of recruitment of 6 representative coregulators to the genes encoding RAB27A and GNB4

ChIP studies verifying recruitment of 6 coregulators at AREs within the genes encoding RAB27A and GNB4. ChIP was done on LNCaP cells that had been treated with 5nM R1881 or vehicle for 16 hours. Coregulators analyzed via ChIP were chosen based on availability of suitable antibodies.

D. Time course of androgen-dependent recruitment of AR (left), NCOA3 (middle) and WDR77 (right) to AREs in the genes encoding RAB27A and GNB4 ChIP was done on LNCaP cells that had been treated with 5nM R1881 or vehicle for 1, 4, 16 or 48 hours.

E. Impact of knock-down of WDR77 or NCOA3 on kinetics of androgen-regulation of RAB27A and GNB4

LNCaP cells were transfected with On Target siRNA SmartPools directed against WDR77 or NCOA3, or with non-targeting On Target Plus control SmartPool (c). At 42 hours after transfection, cells were treated with 5nM R1881 (+) or vehicle (-). After 1, 4, 16 or 48 hours, cells were harvested and RNA was extracted for real-time RT-PCR analysis. Target gene mRNA levels were normalized to GAPDH expression and are expressed as relative expression, taking the value obtained from one of the control siRNA-transfected conditions for each siRNA group at each time point as 1. *Columns*, means of values obtained from three independent biological replicates; *bars*, sem. Gray bars, control siRNA-transfected cells; Black bars, specific siRNA-transfected cells. (Top left) Effects of WDR77 (top) and NCOA3 (bottom) silencing on GNB4 mRNA expression; (Top right) Effects of WDR77 (top) and NCOA3 (bottom) silencing on RAB27A mRNA expression: (Bottom panels) verification of WDR77 siRNA-mediated silencing (left), verification of NCOA3 siRNA-mediated silencing (right).
