# Supplementary material for: A comprehensive analysis of coregulator recruitment, androgen receptor function and gene expression in prostate cancer
Source: eLife. 2017 Aug 18;6:e28482. doi: 10.7554/eLife.28482 (PMC5608510; doi:10.7554/eLife.28482)
Supplement: Supplementary file 6. — (A) Co-immunoprecipitation assay shows interaction between IRF1 and STAT3 in LNCaP cells. (B) Overlap in genome-wide IRF1- and STAT3-dependent androgen-responsive gene expression. Results reflect the effect of 48 hr treatment of LNCaP cells with 5nM R1881. R1881 or vehicle treatment was administered 42 hr after siRNA transfection. Numbers, number of genes. (C) Overlap in IPA functional annotation between the androgen-responsive genes that depend on WDR77 and p53 (from Figure 3, panel F) and the androgen-responsive genes that depend on IRF1 and STAT3 (from panel B of this figure) (p<2.2.E-16). Genes subjected to IPA are those listed in the center of Venn diagrams shown in panel F of Figure 3 and panel B of this figure. Numbers, number of functional annotations. [file elife-28482-supp6.pdf]

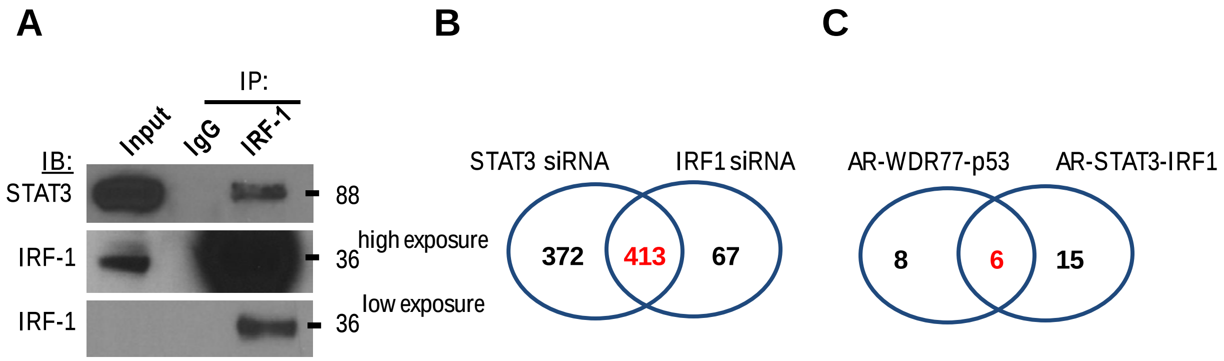

Supplementary File 6

Supplementary File 6. Isolation of a STAT3-dependent AR-IRF1 transcriptional code. (A) Co-immunoprecipitation assay shows interaction between IRF1 and STAT3 in LNCaP cells. (B) Overlap in genome-wide IRF1- and STAT3-dependent androgen-responsive gene expression. Results reflect the effect of 48h treatment of LNCaP cells with 5nM R1881. R1881 or vehicle treatment was administered 42h after siRNA transfection. Numbers, number of genes. (C) Overlap in IPA functional annotation between the androgen-responsive genes that depend on WDR77 and p53 (**from Figure 3, panel F**) and the androgen-responsive genes that depend on IRF1 and STAT3 (**from panel B of this figure**). Genes subjected to IPA are those listed in the center of Venn diagrams shown in panel F of Figure 3 and panel B of this figure. Numbers, number of functional annotations.
