# Supplementary material for: A comprehensive analysis of coregulator recruitment, androgen receptor function and gene expression in prostate cancer
Source: eLife. 2017 Aug 18;6:e28482. doi: 10.7554/eLife.28482 (PMC5608510; doi:10.7554/eLife.28482)
Supplement: Supplementary file 7. — n = number of CaP clinical specimens for which DNA sequencing or copy number variation data is available. Numbers in columns indicate the percentage of CaP cases in each study for which somatic mutations and/or copy number variations that affect corresponding coregulator have been reported. [file elife-28482-supp7.docx]

| **Supplementary File 7. cBio Portal analysis of copy number variation and somatic mutations that affect the 18 identified coregulators in patients prostate cancer specimens** | | | | | | | | |  |
| --- | --- | --- | --- | --- | --- | --- | --- | --- | --- |
| **Coregulator** | **Michigan Nature 2012 (n=61)** | **TCGA, Cell 2015**  **(n=333)** | **Broad/Cornell Cell 2013  (n=57)** | **TCGA Provisional (n=499)** | **PCF/SU2C Cell 2015 (n=150)** | **Broad/Cornell Nature Genetics 2012 (n=112)** | **MSKCC Cancer Cell 2010 (n=181)** | **MSKCC PNAS 2014 (n=103)** | **Nelson Lab 2016**  **(n=176)** |
| SMARCC1 | 4.9 | 1.8 | 1.8 | 1.8 | 0.7 | 0.0 | 0.0 | 0.0 | 2.0 |
| BAG1 | 1.6 | 0.9 | 0.0 | 1.2 | 0.7 | 0.0 | 1.0 | 0.0 | 0.7 |
| SMARCA4 | 0.0 | 0.0 | 0.0 | 0.2 | 1.3 | 0.0 | 0.0 | 0.0 | 2.0 |
| CTNNB1 | 4.9 | 2.7 | 3.6 | 2.7 | 4.7 | 1.8 | 1.9 | 0.0 | 10.1 |
| CAV1 | 1.6 | 1.8 | 3.6 | 1.7 | 1.3 | 0.0 | 1.0 | 0.0 | 0.7 |
| PARK7 | 1.6 | 1.2 | 0.0 | 1.2 | 0.0 | 0.0 | 0.0 | 0.0 | 2.7 |
| FHL2 | 1.6 | 1.5 | 0.0 | 1.5 | 0.7 | 0.0 | 1.0 | 0.0 | 0.7 |
| KDM1A | 0.0 | 1.5 | 1.8 | 1.5 | 2.0 | 0.9 | 0.0 | 0.0 | 0.7 |
| WDR77 | 0.0 | 1.5 | 3.6 | 1.5 | 0.7 | 0.0 | 1.0 | 0.0 | 0.0 |
| EP300 | 0.0 | 0.3 | 0.0 | 0.3 | 1.3 | 1.8 | 2.9 | 0.0 | 2.7 |
| RCHY1 | 4.9 | 0.6 | 0.0 | 0.6 | 2.0 | 0.0 | 0.0 | 0.0 | 0.0 |
| PKN1 | 0.0 | 0.9 | 0.0 | 0.9 | 1.3 | 0.0 | 0.0 | 0.0 | 2.7 |
| NCOA1 | 0.0 | 1.2 | 0.0 | 1.2 | 0.7 | 0.9 | 1.0 | 0.0 | 3.4 |
| NCOA2 | 24.6 | 5.7 | 3.6 | 5.7 | 16.7 | 1.8 | 8.7 | 1.9 | 28.9 |
| NCOA3 | 1.6 | 1.8 | 1.8 | 1.8 | 1.3 | 0.9 | 0.0 | 0.0 | 1.3 |
| STAT3 | 0.0 | 4.8 | 1.8 | 4.8 | 0.7 | 0.9 | 1.0 | 0.0 | 4.7 |
| HTATIP2 | 0.0 | 0.0 | 0.0 | 0.2 | 2.0 | 0.0 | 0.0 | 1.0 | 0.7 |
| KAT5 | 0.0 | 2.4 | 0.0 | 2.4 | 2.0 | 0.0 | 1.0 | 0.0 | 4.7 |

n = number of CaP clinical specimens for which DNA sequencing or copy number variation data is available. Numbers in columns indicate the percentage of CaP cases in each study for which somatic mutations and/or copy number variations that affect corresponding coregulator have been reported.
