# Supplementary material for: A comprehensive analysis of coregulator recruitment, androgen receptor function and gene expression in prostate cancer
Source: eLife. 2017 Aug 18;6:e28482. doi: 10.7554/eLife.28482 (PMC5608510; doi:10.7554/eLife.28482)
Supplement: Supplementaty file 8. [file elife-28482-fig8.pdf]

Supplemental File 8. TP53 binding motif identified from the promoters of WDR77- and p53- dependent androgen-responsive genes.

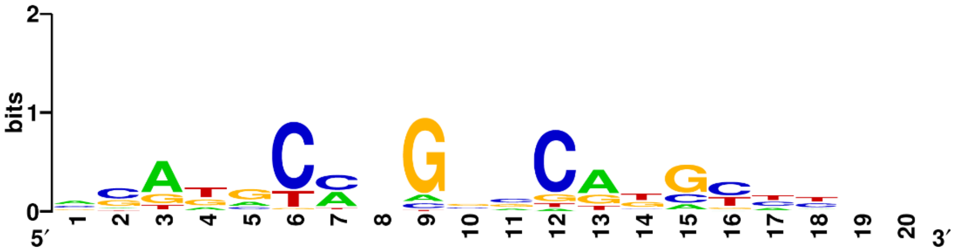

Supplemental File 8
