# Supplementary material for: A comprehensive analysis of coregulator recruitment, androgen receptor function and gene expression in prostate cancer
Source: eLife. 2017 Aug 18;6:e28482. doi: 10.7554/eLife.28482 (PMC5608510; doi:10.7554/eLife.28482)
Supplement: Transparent reporting form [file elife-28482-transrepform.docx]

***eLife’s* transparent reporting form**

We encourage authors to provide detailed information *within their submission* to facilitate the interpretation and replication of experiments. If you have any questions, please contact us: [editorial@elifesciences.org](mailto:editorial@elifesciences.org).

**Sample-size estimation**

- You should state whether an appropriate sample size was computed when the study was being designed
- You should state the statistical method of sample size computation and any required assumptions
- If no explicit power analysis was used, you should describe how you decided what sample (replicate) size (number) to use

Please outline where this information can be found within the submission (e.g., page numbers or figure legends), or explain why this information doesn’t apply to your submission:

Each microarray experiment was performed in triplicate (i.e., three times with independently isolated biological replicates). As the microarray study is exploratory by nature, no sample size calculation is performed. Nevertheless, to confirm the data obtained by microarray analysis, we have verified a substantial subset of the target gene changes by independent real time RT-PCR analysis (Material and Methods section, page 35).

**Replicates**

- You should report how often each experiment was performed
- You should include a definition of biological versus technical replication
- The data obtained should be provided and sufficient information should be provided to indicate the number of independent biological and/or technical replicates
- If you encountered any outliers, you should describe how these were handled
- Criteria for exclusion/inclusion of data should be clearly stated
- High-throughput sequence data should be uploaded before submission, with a private link for reviewers provided (these are available from both GEO and ArrayExpress)

Please outline where this information can be found within the submission (e.g., page numbers or figure legends), or explain why this information doesn’t apply to your submission:

Each experiment was done at least twice (Materials and Methods section article file, page 39). Biological triplicates were analyzed for microarray and q-RT-PCR studies (legends to figures 4 and 6, legends to Figure 1 – Source data 1, Figure 1 – Supplementary Figure 1, supplementary files 2 , 4, and 5, reference 83 article file, descriptions in Methods and Material section). For cell viability and cell cycle analysis assays 3 or 5 biological replicates were analyzed, respectively (legends to figures 4 and 6, reference 83 in methods section of article file). The term biological replicates mean that the same assay was performed on multiple (3 to 5, see above) independent wells or dishes of cells. Inclusion/exclusion criteria to define AR target genes are described on pages 5 and 6 of the results section, pages 27-29 of the Material and Methods section. The oligoarray data have been deposited in GEO under accession numbers GSE66722, GSE66977 and GSE81780. This information is provided in the Material and Methods section, page 29-30.

**Statistical reporting**

- Statistical analysis methods should be described and justified
- Raw data should be presented in figures whenever informative to do so (typically when N per group is less than 10)
- For each experiment, you should identify the statistical tests used, exact values of N, definitions of center, methods of multiple test correction, and dispersion and precision measures (e.g., mean, median, SD, SEM, confidence intervals; and, for the major substantive results, a measure of effect size (e.g., Pearson's r, Cohen's d)
- Report exact p-values wherever possible alongside the summary statistics and 95% confidence intervals. These should be reported for all key questions and not only when the p-value is less than 0.05.

“Oligoarray gene expression analyses” section in Material and Methods section page 26-30;

“Ingenuity Pathway analysis” section in Material and Methods section page 30-31;

“Statistical considerations” section in Material and Methods section page 35

Exact p-values are reported in Figure 1 – Source Data 2, Figure 6 – Source Data 1, Supplementary Files 1 (AR target gene oligoarray analysis), 3 (Ingenuity Pathway Analysis) and Figure 2 – Supplementary Figure 2 (Cistrome analysis), and throughout the text of the results section.

Please outline where this information can be found within the submission (e.g., page numbers or figure legends), or explain why this information doesn’t apply to your submission:

(For large datasets, or papers with a very large number of statistical tests, you may upload a single table file with tests, Ns, etc., with reference to page numbers in the manuscript.)

**Additional data files (“source data”)**

- We encourage you to upload relevant additional data files, such as numerical data that are represented as a graph in a figure, or as a summary table
- Where provided, these should be in the most useful format, and they can be uploaded as “Source data” files linked to a main figure or table
- Include model definition files including the full list of parameters used
- Include code used for data analysis (e.g., R, MatLab)
- Avoid stating that data files are “available upon request”

Please indicate the figures or tables for which source data files have been provided:

Figure 1

Figure 2

Figure 5

Figure 6
